# Time‐Multiplexed Organic Electrochemical Transistor for Saliva‐Based Rapid Detection of Viral Proteins

**DOI:** 10.1002/advs.202519839

**Published:** 2026-05-19

**Authors:** Tianrui Chang, Yuxiang Ren, Shofarul Wustoni, Atheer Alqatari, Adel Hama, Yazhou Wang, Long Chen, Jessica Parrado Agudelo, Luca Salvigni, Keying Guo, Ashraf Dada, Stefan T. Arold, Raik Grünberg, Sahika Inal

**Affiliations:** ^1^ Organic Bioelectronics Laboratory Biomedical Sciences Division King Abdullah University of Science and Technology (KAUST) Thuwal Saudi Arabia; ^2^ KAUST Center of Excellence for Smart Health (KCSH) Biomedical Sciences Division KAUST Thuwal Saudi Arabia; ^3^ Imaging and Characterization Core Laboratories KAUST Thuwal Saudi Arabia; ^4^ King Faisal Specialist Hospital & Research Centre – Jeddah Branch | KFSHRC Department of Pathology and Laboratory Medicine Jeddah Saudi Arabia; ^5^ College of Medicine Al Faisal University Riyadh Saudi Arabia

**Keywords:** nanobody, OECT, organic bioelectronics, organic electrochemical transistor, protein sensor, virus

## Abstract

Timely and accurate diagnosis of respiratory infections is complicated by the symptomatic overlap between highly transmissible pathogens such as respiratory syncytial virus (RSV), influenza A (IAV), and influenza B (IBV), highlighting the need for rapid and reliable diagnostics. Here, we report a time‐multiplexed nanobody‐functionalized organic electrochemical transistor (OECT) biosensor for the sensitive detection of RSV, IAV, and IBV directly from unprocessed saliva samples. The platform integrates arrays of nanobody‐functionalized gate electrodes that are exposed in parallel to a single sample and sequentially addressed through a shared transistor channel. We characterize nine previously reported nanobodies and immobilize the best‐performing ones using an oriented *SpyDirect* self‐assembly strategy. The nanobody‐functionalized gate electrodes are paired with a high‐performance p‐type organic mixed ionic‐electronic conductor channel, enabling efficient signal transduction. The sensor achieves a limit of detection of ca. 1 fm and identifies viral targets within 15 min, without sample preprocessing. In a clinical validation using 17 samples, the platform demonstrated 100% specificity, 86.7% sensitivity, and 88.2% overall accuracy. By combining highly specific nanobodies, a high‐gain transducer, customized surface pre‐treatment, and a time‐multiplexed multi‐gate architecture, this approach provides a scalable and user‐friendly platform for point‐of‐care virus detection.

## Graphical abstract

A time‐multiplexed nanobody‐functionalized organic electrochemical transistor enables rapid and sensitive detection of three respiratory viral proteins from saliva. Sequentially addressed multi‐gate electrodes on a shared channel achieve around 1 fm detection within 15 min without sample preprocessing. Clinical validation demonstrates high specificity, highlighting a scalable, user‐friendly platform for multiplexed and rapid point‐of‐care protein diagnostics.

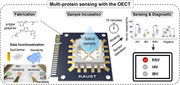

## 1 Introduction

Influenza A and B viruses (IAV and IBV) and respiratory syncytial virus (RSV) are among the most common causes of acute respiratory infections, presenting with similar symptoms in patients, such as fever, cough, sore throat, runny nose, and fatigue [1, 2]. While many individuals recover without medical intervention, these infections pose serious risks to infants, young children, the elderly, and immunocompromised individuals [3, 4, 5, 6, 7]. According to the World Health Organization (WHO) and recent clinical studies, seasonal influenza alone causes an estimated 3–5 million cases of severe illness and up to 650 000 respiratory virus‐related deaths globally each year [8, 9, 10]. True incidence may be even higher, as some cases remain undiagnosed. Early and accurate identification of the causative virus is crucial to initiate timely, targeted treatment and prevent complications or long‐term sequelae, particularly in vulnerable populations.

The polymerase chain reaction (PCR) is the gold standard diagnostic method for viral diagnostics, offering high specificity and selectivity by detecting viral genomic markers at very low concentrations. However, PCR requires complex sample preparation, labor‐intensive protocols, extended detection times, and a dedicated laboratory setting, making it costly and impractical for rapid, point‐of‐care deployment. Alternatively, antigen‐based lateral flow assays offer simplicity and speed but at the expense of sensitivity and reliability [11, 12]. For example, rapid influenza antigen tests, commonly based on lateral‐flow formats, exhibit a pooled sensitivity of ∼62%, with performance strongly dependent on viral load [13]. This highlights a critical need for alternative diagnostic tools that are rapid, accurate, sensitive to low target concentrations, and operate directly with minimally processed samples.

Organic electrochemical transistors (OECTs) have recently emerged as powerful platforms for biosensing due to their intrinsic high amplification, high signal‐to‐noise ratio (SNR), low power demand, and compatibility with aqueous environments [14, 15, 16]. Functionalization of OECT gate electrodes with aptamers [17], antibodies [18], and nanobodies [19, 20, 21] has enabled sensitive detection of diverse protein biomarkers such as TGF‐β_1_, HER2, Amyloid‐β, and SARS‐CoV‐2 with a limit of detection (LOD) in the attomole to femtomole range. The OECT transduces biochemical binding events at the gate electrode into amplified current changes at the semiconducting channel [22, 23]. The high current response allows for miniaturization to support high‐density arrays, positioning OECTs as ideal candidates for portable, multiplexed biosensors. Despite significant progress, OECT‐based biosensing has largely focused on single‐analyte detection. Yet, in clinical practice, multiplexed detection is essential for differential diagnosis, efficient triage, and comprehensive disease profiling with improved prediction accuracy [24, 25]. Multiplexing can be implemented either in parallel (simultaneous acquisition) or in a time‐division (sequential) manner, both of which reduce testing time and resource consumption. Achieving reliable electronic readout of multiple targets in the same media from a single platform, whether sequentially or simultaneously, is challenging due to issues such as cross‐reactivity between recognition elements, electrical crosstalk between closely spaced sensors, and variability in target abundance, binding kinetics, and biorecognition unit‐target interactions. In particular, the wide variation in dynamic range and sensitivity requirements across biomarkers complicates the design of a single platform capable of reliably detecting all targets with high fidelity [26, 27]. These challenges hinder robust multiplexed electronic biosensing and require coordinated strategies in device design, surface chemistry, and biorecognition unit engineering.

Here, we present a nanobody‐functionalized OECT sensor array for the time‐multiplexed detection of RSV, IAV, and IBV directly from patient saliva. The platform integrates multiple nanobody‐functionalized gate electrodes exposed in parallel to a single sample and sequentially addressed through a shared transistor channel. Using the site‐specific and oriented *SpyDirect* self‐assembly strategy [20], we immobilized four high‐affinity nanobodies, selected from a panel of nine, onto gold (Au) gate electrodes and paired them with a high‐performance *p*‐type organic mixed ionic–electronic conductor‐based channel. The platform detected viral proteins with high specificity and minimal cross‐reactivity across ca. 400 am–1 nm concentrations, achieving an LOD of ∼1 fm in buffer and saliva. In clinical validation, OECT responses correlated with RT‐qPCR cycle threshold values, demonstrating promising performance with patient samples. This work establishes a scalable, sensitive, and rapid diagnostic platform for respiratory viruses and highlights time‐multiplexed OECT architectures’ potential for point‐of‐care deployment and in multi‐analyte sensing applications.

## 2 Results and Discussion

### 2.1 System Configuration and OECT Characteristics

**FIGURE 1:**
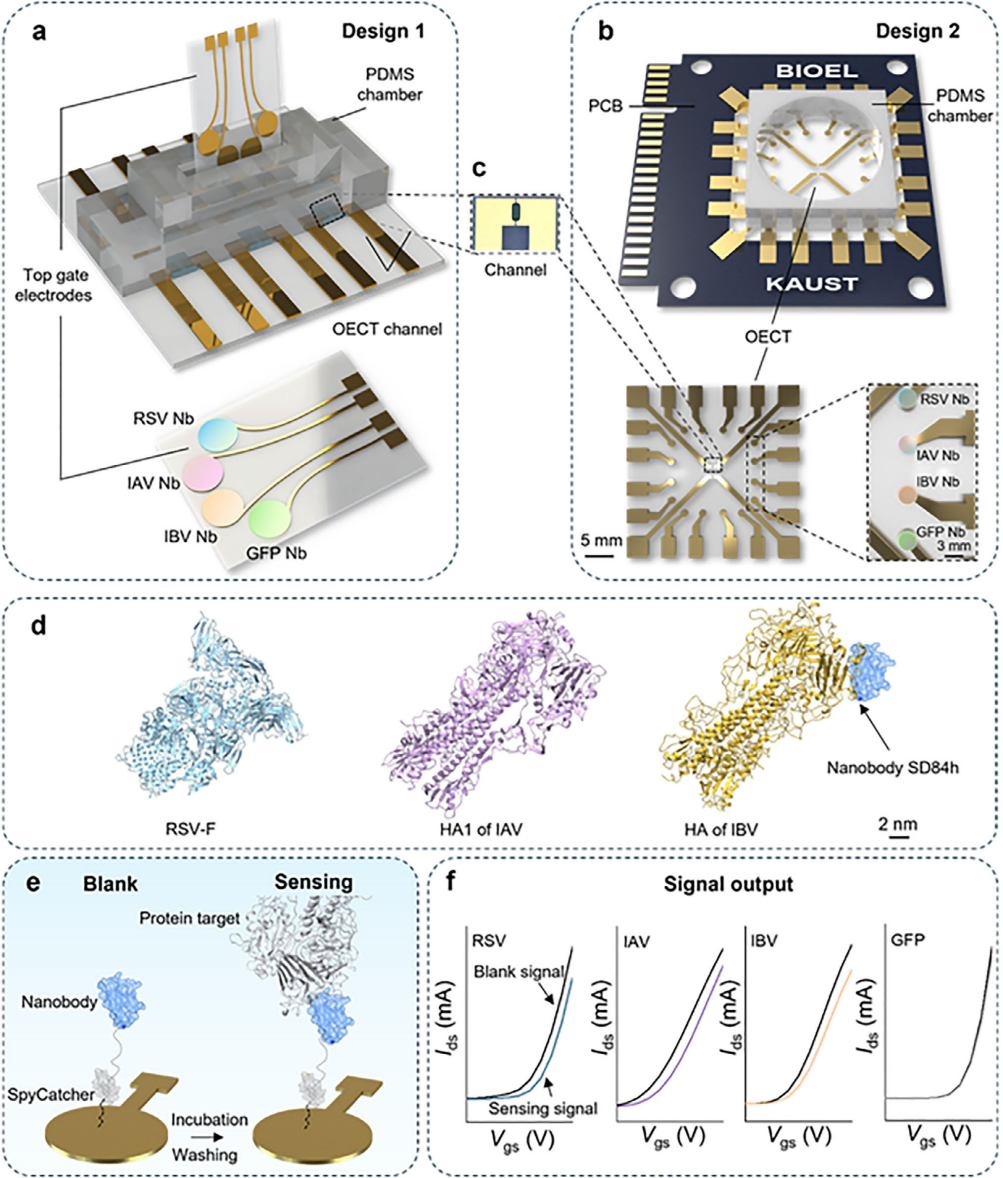
Schematic of the multi‐gate electrode integrated OECT biosensor. (a) Top‐gate configuration with disposable gate and reusable channel compartments fabricated on separate substrates. (b) Lateral‐gate configuration with all components patterned on a single substrate, integrated with a PCB and PDMS chamber. 4 gate electrodes on a single substrate or on the same unit in the lateral‐gate configuration were individually functionalized with RSV nanobody (RSV Nb), IAV nanobody (IAV Nb), IBV nanobody (IBV Nb), and GFP nanobody (GFP Nb). (c) A microscope image of the patterned polymeric mixed conductor film in the channel. Channel width was 100 µm, and length was 10 µm. (d) Structures of the target proteins: fusion protein of RSV (RSV, PDB 8T7A), the HA1 of H1N1 (IAV, PDB 1RU7), and the HA of IBV (IBV, PDB 6CNV), including the binding between IBV nanobody and IBV target protein. (e) Schematic of the gate electrode surface during incubation with a viral antigen‐containing sample. Nontarget binding may occur before washing. The figure components are not to scale. (f) Representative OECT biosensor readout: sequential gating by four individually functionalized electrodes (IAV Nb, IBV Nb, RSV Nb, GFP Nb). Positive signals produce distinct channel current modulation, while the GFP control shows a negligible response, establishing the baseline for positive/negative determination.

We designed and evaluated the performance of two OECT configurations for time‐multiplexed biosensing. In the first design, the gate electrodes were fabricated on a substrate separate from the channel and introduced from above into the electrolyte covering the channel (top‐gate configuration, Figure 1a). This configuration allows for the reuse of the channel compartment while enabling single‐use, disposable gate electrodes. In the second design, we patterned all gate electrodes and the channel onto a single substrate, minimizing the overall device footprint and streamlining integration (lateral‐gate configuration, Figure 1b). Both designs were used throughout this study, as indicated in figure captions, with consistent gate‐to‐channel dimensions across platforms to ensure comparable performance.

In the top‐gate configuration, four gate electrodes were patterned on a single glass substrate (Figure 1a). For the integrated lateral‐gate configuration, we designed a sensing matrix composed of 16 Au gate electrodes and two OECT channels, which were interfaced with a custom‐designed printed circuit board (PCB) and embedded in an electrolyte chamber fabricated from polydimethylsiloxane (PDMS) (Figure 1b). The PCB connected to a read‐out module for data acquisition, while the PDMS chamber confined sample, wash, and electrolyte solutions to the sensing area. The 16 gate electrodes were divided into four sensing units. Within each unit, four gate electrodes were individually biofunctionalized with nanobodies against IAV, IBV, RSV, or green fluorescent protein (GFP) (Figure 1b), the latter serving as the negative control. The same biofunctionalization protocol was applied across both platforms.

The transistor channels (Figure 1c) were formed from a p‐type mixed conductor, p(g_3_C_2_T2‐T) (chemical structure in Figure S1a), spin‐coated onto glass substrates and patterned following standard photolithography and Parylene‐C peel‐off techniques (see Methods for details). These *p*‐type enhancement‐mode OECTs produced a substantial source‐drain current (*I*_ds_) that increased in response to a small negative voltage applied between the gate and source (*V*_gs_) (Figure S1b). They are ideally suited for biosensing because of their low operational voltage of −0.12 V, an excellent switching behavior with a subthreshold slope of 77 mV/dec (close to the thermal limit), and the ability to deliver mA‐level currents even in dilute electrolytes. To enhance sensitivity, we selected to operate the OECTs in dilute PBS (0.001×), which increases the Debye length and thereby improves the sensitivity of transistor‐based potentiometric biosensors [28]. Devices retained ∼95% of their initial current after 1000 operational cycles (Figure S1c,d), confirming high stability under repeated use.

Figure 1d shows the structures of the three target proteins and the binding site between the IBV target protein and IBV nanobody [29]. Following biofunctionalization, gate electrodes were first used to record baseline (blank) signals, then incubated for 10 min in samples potentially containing viral proteins (Figure 1e). After incubation, electrodes were thoroughly rinsed to remove nonspecifically bound molecules. Sensing signals were then recorded, and the detection outcome was determined by calculating the normalized response (NR), defined as the relative change between the blank and post‐incubation (sensing) signals, and comparing this value to that of the GFP‐functionalized negative control (Figure 1f).

### 2.2 Selection and Characterization of Biorecognition Units

Our literature and patent search identified a total of nine nanobodies targeting four viral proteins (Table S1). These are the RSV fusion protein (RSV‐F), the hemagglutinin 1 (HA1) or the nucleoprotein (NP) of the H1N1 strain of IAV (A/California/06/2009), and the HA of IBV (B/Brisbane/60/2008) (Figure 1d) [29, 30, 31, 32, 33]. We designed expression constructs encoding each nanobody fused flexibly to the SpyCatcher domain [20] and expressed them in the *Escherichia coli* (*E. coli*) cytoplasm. The proteins were purified using nickel affinity followed by size‐exclusion chromatography (SEC) and analyzed by SDS‐PAGE and SEC coupled with multi‐angle light scattering (SEC‐MALS) to determine purity, molecular weight, oligomerization state, and monodispersity (Figures S2–S5). Two nanobodies failed expression or purification. Affinity measurements against their respective targets were performed for the remaining proteins using microscale thermophoresis (MST) or intrinsic fluorescence analysis. All nanobodies bound their target with dissociation constants (K_D_) ranging from ∼20 to ∼1000 nm. The three nanobody constructs with the highest binding affinities (Table 1) were selected for integration into the OECT sensor platform.

**TABLE 1:** Selection of nanobodies and analysis of their target binding performance.

Target	Nanobody	Nanobody‐ SpyCatcher fusion^a^ ^)^	K_D_ (nm)	SNR^b^ ^)^	Ref.
RSV‐F	Nb017	sb0330p (Nb017‐[GS]_5_‐SpyC‐His_8_)	236.0 ± 121.5	23.1	[31]
HA of H1N1	R2b‐D9	sb0336p (R2bD9‐[GS]_5_‐SpyC‐His_8_)	19.2 ± 1.1	5.4	[32]
HA of IBV	SD84h	sb0338p (SD84h‐[GS]_5_‐SpyC‐His_8_)	21.2 ± 2.5	2.2	[29]

^a^ construct name and identification number

^b^ SNR: MST signal‐to‐noise ratio.

### 2.3 Biofunctionalization and Characterization of Gate Electrodes

**FIGURE 2:**
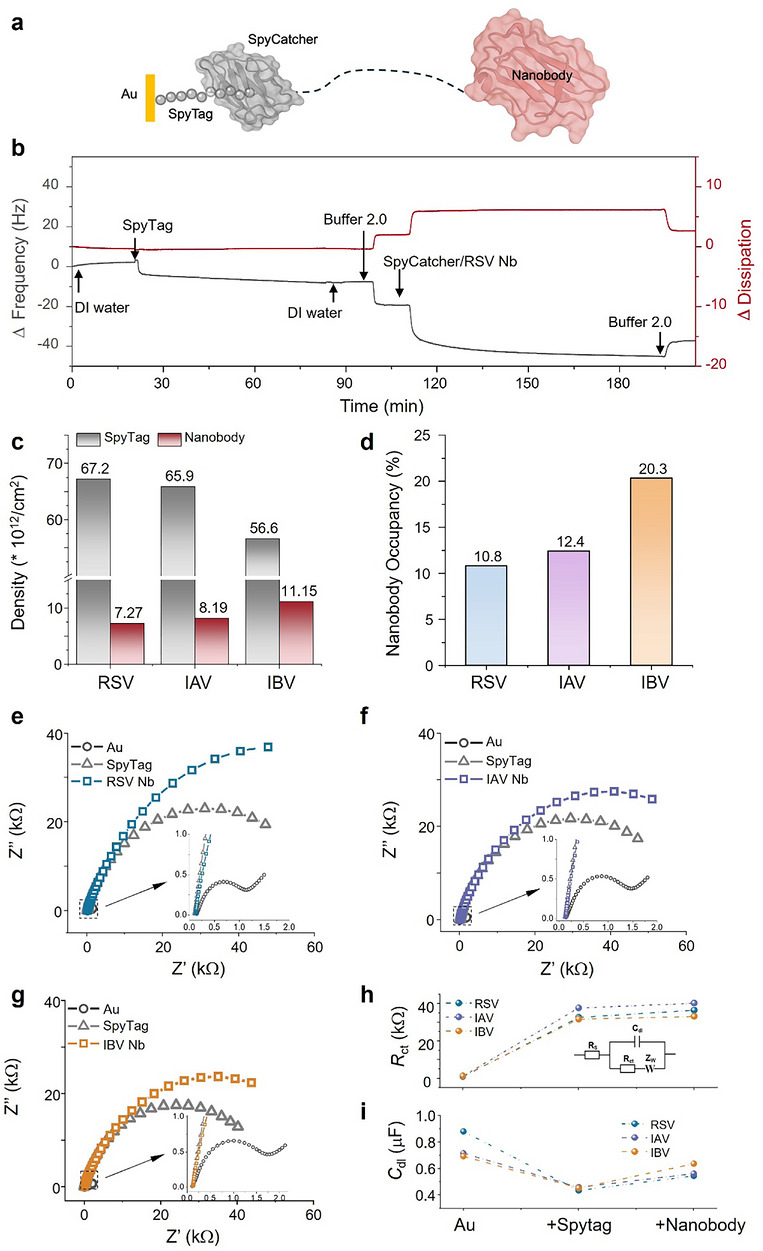
Characterization of electrode biofunctionalization. (a) Schematic of the Au electrode functionalized with nanobodies via the SpyTag/SpyCatcher system. A cysteine‐terminated SpyTag peptide is first immobilized on the Au surface through Au–S bonding, followed by covalent attachment of the SpyCatcher‐nanobody fusion protein. (b) Representative QCM‐D measurement showing the stepwise biofunctionalization for the RSV Nb. The black trace indicates frequency change (Δ*f*), and the red trace represents energy dissipation (Δ*D*). (c) Surface density of biomolecular layers on the Au electrodes following each functionalization step. (d) Estimated occupancy of SpyTag peptides by nanobodies calculated from (c). (e–g) Nyquist plots of Au electrodes before and after the immobilization of (e) RSV Nb, (f) IAV Nb, and (g) IBV Nb. Measurements were performed using 10 mm [Fe (CN)_6_]^3−/4−^ as redox couple. (h) Charge transfer resistance (*R*_ct_) and (i) capacitance (*C*_dl_) values from EIS fits using a Randles equivalent circuit (inset to h).

Figure 2a illustrates the biorecognition construct assembled on the Au electrode surface. A SpyTag peptide with a cysteine terminal was first anchored on the Au surface through gold–sulfur (Au–S) interactions [20]. This was followed by covalent coupling of the SpyCatcher‐fused nanobody through the SpyTag/SpyCatcher reaction. This strategy orients the nanobody toward the electrolyte interface while preserving its native conformation and avoiding harsh chemical modifications. To minimize nontarget adsorption during sensing, bovine serum albumin (BSA) was introduced to passivate remaining unoccupied sites on the electrode.

We monitored the immobilization process and quantified molecular packing density using QCM‐D, which allows for label‐free tracking of mass accumulation and viscoelastic changes on Au‐coated surfaces. Figure 2b shows a representative QCM‐D trace for the immobilization of the RSV nanobody; corresponding data for IAV and IBV nanobodies are shown in Figure S6 and summarized in Table S2. Upon introduction of the SpyTag peptide, the resonance frequency decreased by 9.62 Hz, corresponding to a surface density of around 67.2 × 10^12^ molecules/cm^2^. Subsequent addition of the RSV nanobody resulted in a further frequency decrease along with a modest increase in dissipation, suggesting nanobody immobilization at a density of 7.3 × 10^12^ molecules/cm^2^. Comparable trends were observed for the IAV and IBV nanobodies (Figure 2c). The calculated site occupancy, defined as the fraction of SpyTag peptides coupled to nanobodies, was 10.8% (RSV Nb), 12.4% (IAV Nb), and 20.3% (IBV Nb) (Figure 2d). These values are consistent with the larger size of the nanobody‐SpyCatcher construct (∼28 kDa) compared to the SpyTag peptide (∼1.8 kDa), which introduces steric hindrance and leads to occlusion of multiple SpyTag sites per nanobody. Considering each nanobody as an approximate sphere with a radius of 2 nm, the average area of the nanobody layer is 0.89 ± 0.18 cm^2^. Given that the QCM sensor has an area of 0.785 cm^2^, this corresponds to 113.1% ± 22.93% coverage, indicating a densely packed, nanobody‐functionalized surface.

The sequential assembly of peptide and protein layers also affected the electrochemical properties of the electrodes. Electrochemical impedance spectroscopy (EIS) data (Figure 2e–g) were fitted using a Randles equivalent circuit to extract the charge transfer resistance (*R*_ct_) (Figure 2h) and the double‐layer capacitance (*C*_dl_) at the electrode/electrolyte interface (Figure 2g). After SpyTag immobilization, the *R*_ct_ increased substantially (for example, from 1.0 to 32.6 kΩ for the RSV Nb electrode), accompanied by a decrease in *C*_dl_ (from 0.88 to 0.45 µF), indicating the formation of a charge‐blocking peptide layer. Following nanobody coupling, the Nyquist plots showed further expansion of the semicircle, with *R*_ct_ values increasing to 36.5 kΩ (RSV Nb), 40.2 kΩ (IAV Nb), and 33.1 kΩ (IBV Nb), confirming additional impedance from the protein layer. While the *C*_dl_ decreased after SpyTag immobilization, a slight increase was observed following nanobody coupling, although it remained lower than that of the bare Au electrodes. Additional evidence for successful surface biofunctionalization was obtained by XPS. High‐resolution C 1s spectra exhibited increased signal intensity and the appearance of new peaks associated with carbon‐oxygen bonds (C─O and COOR groups) following biomolecule assembly (Figure S7a). The emergence of an N1s signal (Figure S7b) further confirmed the presence of nitrogen‐containing proteinaceous material on the Au electrodes.

### 2.4 Protein Binding on Nanobody‐Functionalized Electrodes

**FIGURE 3:**
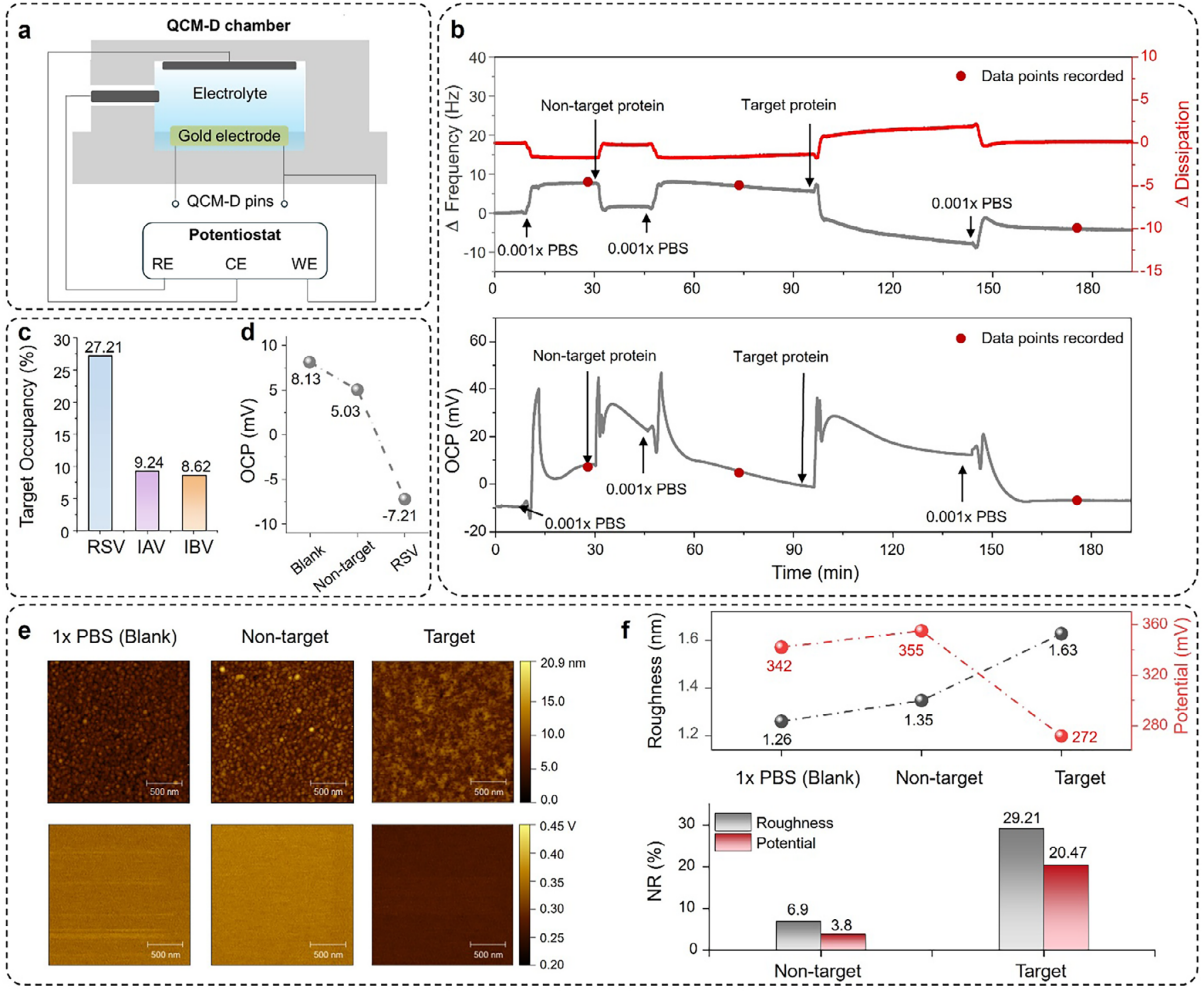
Monitoring the target protein binding on nanobody‐functionalized electrodes. (a) Schematic of the experimental setup integrating electrochemical quartz crystal microbalance with dissipation monitoring (eQCM‐D) and real‐time open‐circuit potential (OCP) measurements. (b) QCM‐D traces of an RSV nanobody‐functionalized electrode sequentially exposed to 1×PBS (blank), 100 nm IAV and IBV proteins (nontargets), and 100 nm RSV protein (target), with the simultaneously recorded OCP data reflecting surface potential changes. Red markers represent the data points used for extracting the frequency and OCP values, which were subsequently used for the calculations presented in (c) and (d). (c) Estimated occupancy of nanobody binding sites by target proteins. (d) OCP trend extracted. (e) Kelvin probe force microscopy (KPFM) images showing surface morphology (top) and surface potential (bottom) of the RSV nanobody‐functionalized electrode before protein exposure (left), after nontarget incubation with IAV/IBV proteins (middle), and following RSV binding (right). (f) Quantification of RMS surface roughness and potential (top) across the three stages shown in **e**, with normalized changes (bottom).

We next assessed the affinity and specificity of the nanobody‐immobilized electrodes toward their respective target protein using a combination of in situ and *ex situ* techniques. To track changes in the electrode's surface potential during binding [34], we coupled a potentiostat to the electrochemical quartz crystal microbalance with dissipation monitoring (QCM‐D) setup and recorded real‐time open‐circuit potential (OCP) values simultaneously with the mass change data (Figure 3a). OCP data is important to monitor as the OECTs are expected to operate as potentiometric sensors.

Figure 3b (top) shows representative results from an RSV Nb‐functionalized electrode. After establishing a baseline by 1× PBS incubation and 0.001× PBS rinsing, we introduced a mixture of nontarget proteins (100 nm IAV and IBV, incubation for 15 min in 1× PBS) into the chamber, causing a slight frequency decrease of 0.65 Hz, indicative of minimal nonspecific adsorption. Subsequent introduction of 100 nm of RSV protein (incubation for 45 min in 1× PBS) resulted in a significant frequency decrease, corresponding to a target‐nanobody complex density of 1.97 × 10^12^ molecules/cm^2^ (Table S2). Similar results were obtained for IAV and IBV Nb sensors (Figure S8a,c). Across all nanobody‐functionalized electrodes, target binding occupancies ranged from approximately 9% to 27%, with RSV Nb showing the highest occupancy (27.21%), followed by IBV Nb (9.24%) and IAV Nb (8.62%) (Figure 3c). These are very high values considering that the four times larger size of target proteins (57.8–66.2 kDa) relative to the nanobodies (15 kDa) (recall Figure 1d) should lead to steric occlusion between targets. Interestingly, these values did not correlate with Nb coupling densities (highest for IBV Nb) or with binding affinities (lowest for RSV Nb) but instead suggest that RSV‐F can be captured at higher densities than the two closely related HA target proteins.

For the RSV Nb‐functionalized electrode, incubation with nontarget proteins (IAV and IBV) led to a 3.10 mV drop in OCP, while the addition of RSV protein caused a pronounced shift toward more negative values (ΔOCP = −12.23 mV) (Figure 3b bottom and d), indicating that specific binding events modulate the electrode's electrochemical potential. Similar results were obtained for IAV and IBV Nb sensors (Figure S8b,d), confirming that the target binding induced a potential drop across all three sensing modalities. Note that target binding was performed under physiological conditions to preserve native target–nanobody interactions and avoid, hypothetically, dilution of the patient sample. In contrast, quantification of the bound analyte and the corresponding OCP measurements were carried out in dilute PBS to increase the Debye length beyond the estimated thickness of the biorecognition layer (∼18 nm), enabling electrical signal transduction.

We used Kelvin probe force microscopy (KPFM) as a complementary technique to validate these findings and simultaneously monitor morphological alterations on the sensor surface induced by target binding. As shown in Figure 3e,f, the RSV Nb‐functionalized electrode displayed a modest increase in root‐mean‐square (RMS) roughness and a slight decrease in surface potential following exposure to nontarget proteins (normalized changes of 6.9% and 3.8%, respectively). Upon incubation with RSV protein, these changes became markedly more pronounced, with a 29.2% increase in RMS roughness and a 20.5% decrease in surface potential. These effects are attributed to the accumulation of the RSV protein at the electrode interface.

Collectively, in situ eQCM‐D and ex situ KPFM analyses demonstrated that target protein binding systematically reduced the surface potential of nanobody‐functionalized electrodes. To evaluate this response across a range of concentrations, we monitored the OCP shifts of RSV, IAV, and IBV Nb‐modified electrodes upon incubation with increasing concentrations of the corresponding target protein (1 am to 1 nm). The OCP decreased systematically with increasing target concentrations (Figure S9a–c), while incubation with PBS alone produced no measurable change or drift (Figure S9d–f), confirming the specificity and stability of the potentiometric signal. These results establish that nanobody‐functionalized electrodes detect target protein binding through robust, label‐free potentiometric shifts, supporting their use as selective biosensors within the OECT platform.

### 2.5 Sensing Proteins with the Nanobody‐Functionalized OECTs

In this study, we used GFP Nb‐functionalized electrodes as the negative control to verify the specificity of target binding (recall Figure 1). However, despite the absence of specific interactions, exposure of these electrodes to other proteins caused a pronounced increase in their OCP, suggesting that nonspecific molecular interactions affected the electrode surface potential even though the nontarget molecules did not stay on the surface permanently and washed away after the rinsing step (Figures S10, S11). To address this issue and stabilize the OCP of the negative control electrode, we developed a pre‐treatment strategy, which involved the overnight incubation of all electrodes with a low concentration of protein‐based blockers. We identified lysozyme as an effective blocker to eliminate the effect of the three investigated (nontarget) proteins (RSV, IAV, and IBV) on the OCP of the control electrodes and integrated it into the surface functionalization protocol (see Figures S12–S17; Tables S3, S4, and Note S1). Storage overnight at 4°C in lysozyme caused an average *R*_ct_ increase of 5.7%, 9.1%, and 6.3% for RSV, IAV, and IBV sensors, respectively (Figure S18b,d,f) and a *C*_dl_ decrease of 4.1%, 6.2%, and 4.9%, respectively (Figure S19). These changes are significantly smaller than those observed during peptide‐to‐nanobody functionalization, indicating minimal signal drift and minor nonspecific adsorption during storage with lysozyme. QCM‐D results in Figure S13 showed small but non‐zero shifts, consistent with the EIS observations. Upon incubating the electrodes with 100 nm of their corresponding targets, we recorded an average *R*_ct_ increase of 16.2%, 22.2%, and 27.3% for RSV, IAV, and IBV sensors, respectively (Figure S18b,d,f), confirming successful and specific target binding. The *C*_dl_ of RSV and IAV sensors decreased slightly by 3.49% and 4.40%, respectively, while IBV sensors showed almost no change in their capacitance (Figure S19).

**FIGURE 4:**
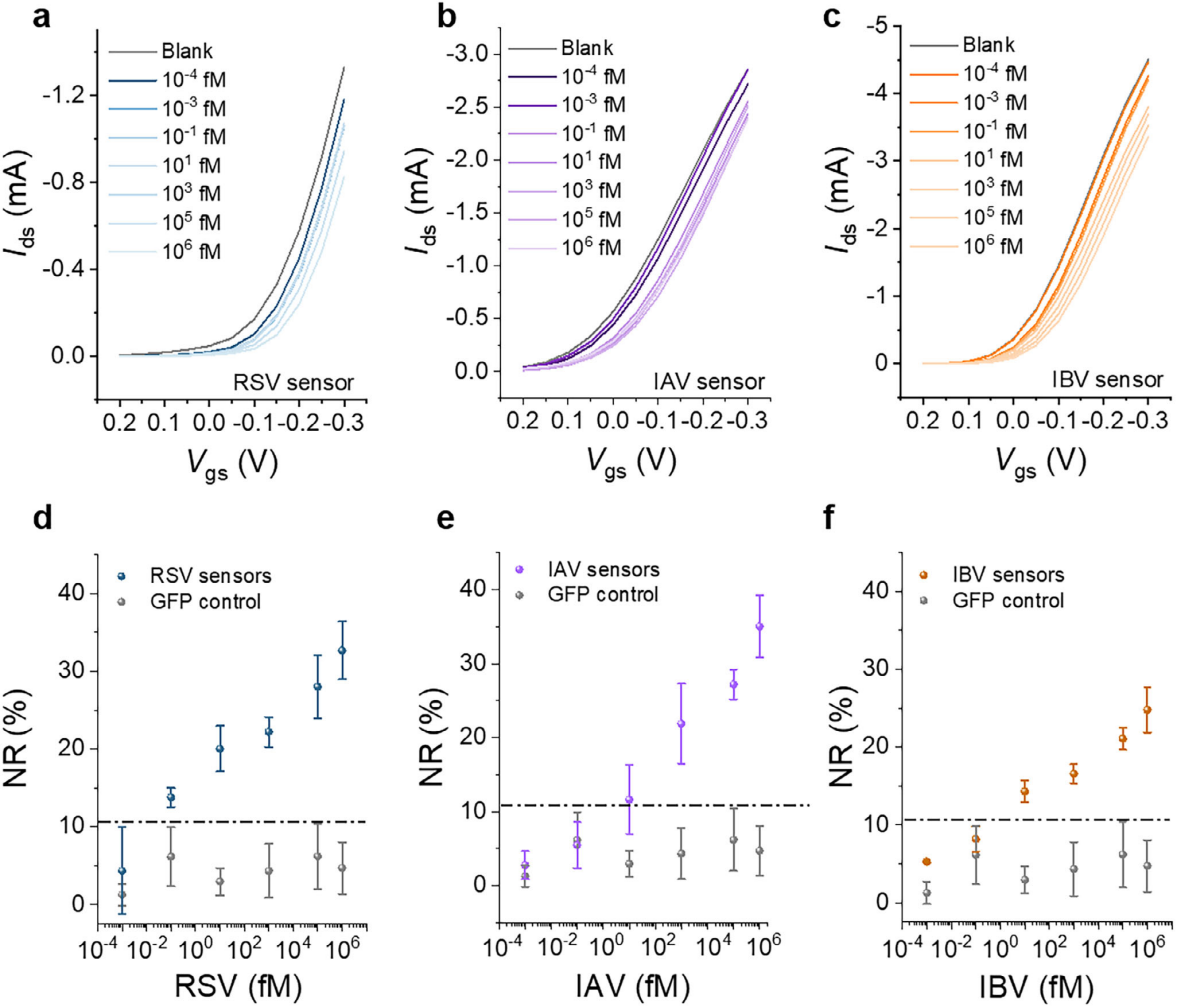
OECT sensor performance. Transfer curves of OECTs gated with electrodes specific to (a) RSV, (b) IAV, and (c) IBV. The protein concentration changed from 0.1 am to 1 nm. (d–f) Corresponding normalized response (NR) values of each electrode, as well as the negative control electrode, were exposed to the same target solution. NR values were extracted at *V*_ds_ = −0.2 V and *V*_gs_ = −0.3 V, where the response was maximized. The error bars show the standard deviation (SD) from three gate electrodes measured on one channel. The measurement electrolyte in all experiments was 0.001× PBS, and device configuration 1 was used.

These lysozyme‐pretreated gate electrodes were rinsed, incubated with 1× PBS for 15 min, washed with 0.001× PBS, and connected to the OECT channel for baseline signal acquisition (black curves in Figure 4a–c). Sensors and control electrodes were then exposed to test solutions containing target proteins (RSV‐F, HA1 of IAV, or HA of IBV) in 1× PBS at concentrations ranging from 0.1 am to 1 nm. After a 15 min incubation at each concentration, the electrodes were washed and connected to the OECT (0.001× PBS as the electrolyte) for signal recording. Upon specific target binding, each sensor exhibited a decrease in channel current and a shift in threshold voltage (*V*_th_) toward more negative values. With increasing target concentrations, the extent of the shift and current decrease increased. GFP Nb functionalized electrodes demonstrated no significant changes in their transfer curves, confirming that the observed changes in the sensing electrodes were due to specific nanobody‐target protein interactions. As the gate electrode's potential becomes more negative upon target protein binding (see the OCP and KPFM measurements of individual gate electrodes in Figure 3; Figure S9), the potentials of source and drain contacts also move to lower negative potentials to maintain the same magnitude of V_GS_, bringing the channel to a less doped state [35].

To quantify sensing performance, we calculated the NR (NR = (*I*_ds_‐*I*_0_)/*I*_0_, where *I*_0_ is the blank current, and *I*_ds_ is the post‐incubation current). The NR increased linearly with the logarithm of protein concentration for all targets (R^2^ = 0.957 for RSV, 0.972 for IAV, and 0.993 for IBV sensors; Figure 4d–f), with an estimated LOD of (261.6 ± 32.5) aM for RSV, (1.73 ± 1.45) fm for IAV, and (5.20 ± 1.17) fm for IBV. All three sensors exhibited low LODs in the femtomolar range and broad dynamic ranges across 5–6 orders of magnitude. To assess reproducibility near the LOD, a second set of electrodes (three electrodes across two arrays) was tested. All six sensors reliably detected the targets at these low concentrations (4 fm for IAV and IBV and 400 am for RSV), with a coefficient of variation (CV) below 30% (Figure S20). Notably, the IAV sensor, despite using the nanobody with the strongest solution‐phase binding affinity, did not significantly outperform others in terms of LOD or calibration curve slope (i.e., sensitivity). This again suggests that the OECT signal transduction is not governed by binding affinity alone. In fact, the marginally lowest LOD was achieved by the RSV sensor, which had shown the lowest immobilization density but the highest target binding occupancy in the previous QCM‐D measurements.

To assess cross‐reactivity, each sensor was exposed to 1 nm of a nontarget protein (IAV protein for the RSV sensor, RSV protein for the IAV sensor, and RSV protein for the IBV sensor), followed by 100 pm of its cognate target. As shown in Figure S21, all sensors exhibited minimal responses to nontarget proteins, while producing pronounced responses to their specific targets at only 100 pm concentrations, demonstrating both high specificity and minimal cross‐reactivity. The sensor reliability was further assessed through randomized tests. Rather than systematically increasing target concentrations, several sensors were randomly selected for incubation with a single concentration of target proteins ranging from 1 am to 1 nm, using an array of four sensors per test. All sensors reliably detected protein concentrations above 1 fm, confirming accurate and reproducible sensing (Figure S22). We also evaluated device reusability, storage stability at 4°C over 7, 14, 21, and 28 days, and at room temperature over 7, 14, and 21 days, and inter‐chip variability over 7, 14, and 21 days (Figure S23 and Note S2). RSV sensors stored at 4°C maintained stable performance for up to 14 days, with only 4–7% signal reduction and acceptable inter‐chip variation (CV = 18.8% at 4 pm). In contrast, room‐temperature storage led to faster degradation (> 10% signal loss by day 7) and increased variability. In both cases, sensing performance deteriorated after 21 days (Figure S23). EIS measurements show a corresponding decrease in *R*_ct_ over time (Figure S23j), indicating partial loss of the nanobody layer or conformational changes therein.

### 2.6 Multi‐virus Sensing in Biofluids and Clinical Samples

**FIGURE 5:**
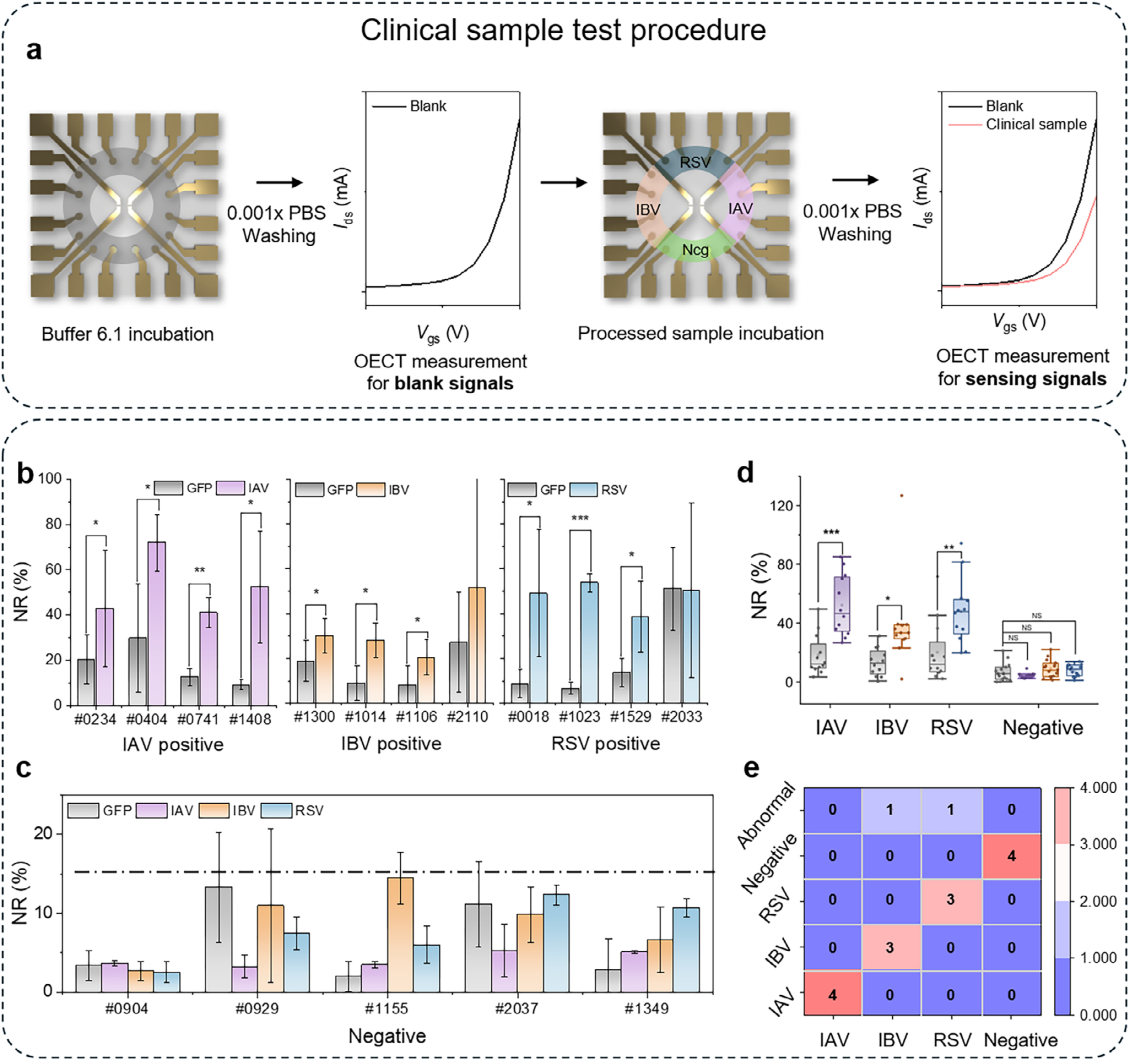
Sensor performance evaluation with clinical samples. (a) Workflow for clinical sample testing: (i) Incubation of gate electrodes with Buffer 6.1 to establish blank signals, (ii) recording of blank signals, (iii) division of electrodes into four sensing units, each exposed to a different clinical sample, (iv) recording of sensing signals. Device configuration 2 was used. (b) NR values calculated from the transfer curves of IAV, IBV, and RSV sensors following exposure to clinical samples. (c) NR values for all virus‐specific sensing electrodes and GFP‐functionalized control electrodes measured in response to negative samples. (d) Box plot summarizing NR values across all clinical sample categories, highlighting significant differentiation between virus‐positive and virus‐negative responses and virus‐positive responses of sensor electrodes versus the control. (e) Heat map summarizing classification accuracy. The *x*‐axis denotes the clinical diagnosis of each sample (RSV, IAV, IBV, or negative), while the *y*‐axis represents the corresponding output of the OECT sensor array, including a row indicating abnormal responses.

After validating sensor performance in buffer solutions, we evaluated the biosensor performance in complex biological media by spiking target proteins into human saliva. As shown in Figure S24a–c, nanobody‐functionalized electrodes targeting RSV, IAV, and IBV produced clear OCP changes in response to 1 fm, 1 pm, and 1 nm of their respective proteins, whereas GFP control sensors showed no consistent trend. The mean OCP shifts (Figure S24d–f) were statistically significant across concentrations (p < 0.05, two‐sided t‐test), demonstrating target‐specific detection even in raw saliva. Next, we acquired 17 human saliva samples, including four each from RSV, IAV, and IBV‐infected patients and five from healthy individuals. Those samples were diluted with Buffer 6.1 (see Section 4) for clinical sample pre‐treatment, as used in our earlier work [21]. The testing protocol, outlined in Figure 5a, involved four steps after taking out the functionalized electrodes from the storage buffer: (i) rinsing and incubating the electrodes with blank buffer (Buffer 6.1), (ii) rinsing and establishing blank signals (in 0.001× PBS electrolyte), (iii) dividing the array into four sensing units, each assigned to a different clinical sample, and incubating the electrodes with clinical samples, and (iv) rinsing and recording the sensing signals. Transfer curves for all clinical samples are provided in Figures S25–S36. The NR values, calculated at *V*_ds_ = −0.3 V and *V*_gs_ = −0.4 V, revealed clear discrimination between virus‐positive and virus‐negative samples (Figure 5b,c). Among the 12 positive samples, 10 were correctly identified, including 3/4 RSV, 4/4 IAV, and 3/4 IBV cases. All five negative samples produced low responses, statistically indistinguishable from those of GFP controls (p > 0.05, Figure 5c, Figures S37–S41), confirming the robustness of the GFP‐functionalized electrode as a negative control and the sensor's no false positive responses. Figure 5d summarizes all sensor responses, highlighting significant differences of the sensors for virus‐positive samples compared to the negative control, with no significant responses observed for infection‐free samples. Figure 5e shows a heat map summarizing the clinical results, including classifications for the four sample types (RSV, IAV, IBV, and negative) and a dedicated row indicating abnormal signal patterns. Two samples, one RSV and one IBV, caused atypical OECT responses (Figures S28 and S36). Additionally, occasionally elevated signals were observed from GFP control electrodes (Figure S30a–c), and variability in output across electrodes was noted for certain samples (Figure S29d–f). These anomalies could arise from variability in sensor functionalization due to manual fabrication processes or from biological heterogeneity among clinical specimens. Furthermore, the use of the Buffer for clinical sample pre‐treatment, while effective in the prior study [21], may require re‐optimization for consistent performance in real‐world diagnostics.

The relationship between the OECT response and the corresponding cycle threshold (CT) values obtained from RT‐qPCR is shown in Figure S42. An inverse correlation was observed between the OECT response and CT, with lower CT values yielding stronger sensor responses and higher CT values producing weaker signals. This behavior is consistent with the fundamental principle of RT‐qPCR, where CT is inversely related to the logarithm of target concentration. Although the dependence is not strictly linear, the monotonic trend demonstrates that the OECT response reflects variations in target concentration across clinically relevant ranges. The observed signal dispersion is attributed to the inherent variability of clinical samples (e.g., matrix composition, ionic strength, and pH), as well as the detection of both free and virus‐associated proteins by the OECT. These results demonstrate that the OECT captures clinically relevant trends in viral load and supports its use as a rapid, label‐free, low‐cost diagnostic tool, complementary to RT‐qPCR. Importantly, the platform flagged all uncertain cases for follow‐up PCR analysis, recorded no false negatives, and maintained high detection fidelity under real‐world conditions. The OECT sensor array demonstrated 100% specificity, approximately 88% sensitivity (15 out of 17 samples correctly identified), and a substantial reduction in diagnostic turnaround time compared to conventional assays.

## 3 Conclusions

We developed an organic electrochemical transistor (OECT) platform functionalized with high‐affinity nanobodies for the rapid, label‐free detection of three clinically relevant respiratory viruses: RSV, IAV, and IBV. The sensor integrates a modular gate electrode design with a robust SpyTag/SpyCatcher conjugation strategy, enabling stable and oriented nanobody immobilization at high surface densities (∼9.5 × 10^11^ molecules/cm^2^). Stepwise functionalization was validated using QCM‐D, electrochemical impedance spectroscopy, and XPS, confirming successful nanobody assembly and target binding. Real‐time OCP and ex situ KPFM measurements revealed that protein binding events induced consistent and measurable changes in the surface potential of the electrodes. These changes were transduced by the OECT into amplified electrical signals when the electrodes were used as the gate electrode that controlled the doping state of the polymeric channel. The OECT sensors were operated in a time‐multiplexed manner, with the channel current recorded sequentially for each gate electrode before and after target protein exposure, and the response was compared to that of a negative control electrode exposed to the same sample. The sensors achieved low femtomolar‐level limits of detection and demonstrated a broad dynamic range (≥ 5 orders of magnitude), with strong specificity and minimal cross‐reactivity, independent of the differences in dissociation constants or nanobody coupling densities. Clinical validation using 17 unprocessed human saliva samples yielded a sensitivity of 86.7%, 100% specificity, and 88.2% overall accuracy, all with minimal sample preparation and within a 15 min assay time. Importantly, performance was maintained in complex biological matrices, underscoring the platform's robustness and applicability for point‐of‐care diagnostics. This work highlights the power of combining precise and high‐density biofunctionalization with the organic electrochemical transistor as a high‐gain transducer, as well as the importance of identification of a surface pre‐treatment protocol for a reliable control electrode and multimodal interface characterization to understand sensor operation and improve its performance. Future improvements will focus on scaling the fabrication process, optimizing the surface potential stabilization strategies, and automation of the biofunctionalization to minimize device–device variations, operating sensors and negative controls simultaneously to reduce the overall time from sample incubation to results, and integrating machine learning for automated data interpretation. Together, these advances position our OECT‐based nanobody sensor as a promising tool for rapid, sensitive, and multiplexed virus or protein detection in clinical and resource‐limited settings.

## 4 Materials and Methods

### 4.1 Materials

Sodium chloride, sodium azide, Tween‐20, glycerol, HEPES, BSA, and PBS (pH 7.4) were purchased from Sigma–Aldrich and used as received. All aqueous solutions were prepared with DI water (Millipore Milli‐Q). p(g_3_C_2_T2‐T) was synthesized according to a procedure reported previously [36]. 10× Tris‐Buffered Saline was purchased from Thermo Scientific. Protein purification materials: Agar, LB broth, 2xYT Broth, Kanamycin, Glucose, Isopropyl β‐D‐1‐thiogalactopyranoside (IPTG), BugBuster (Novagen), cOmplete Protease Inhibitor mix (Sigma), Benzonase (Novagen), Egg‐Lysozyme (Fluka), Tris (2 carboxyethyl)phosphine (TCEP), Tris (hydroxymethyl) aminomethane hydrochloride (Tris‐HCl), Imidazole, Glycerol, Dithiothreitol (DTT), Ethylenediaminetetraacetic acid (EDTA), D‐Desthiobiotin, 10K Amicon ultra spin concentrators (Millipore). Purification columns and SPR materials were purchased from GE. The human RSV glycoprotein (F0) (RSV), the recombinant HA1 subunit of the IAV virus (A/California/06/2009(H1N1)) (IAV), and the recombinant hemagglutinin of Influenza B (B/Brisbane/60/2008) (IBV) were purchased from Sino Biological. They were dissolved to a standard concentration of 0.25 mg/mL as per the manufacturer's instructions, then aliquoted, snap‐frozen in liquid nitrogen, and stored at −80°C. C‐terminally cysteine‐linked SpyTag peptide was synthesized by GenScript Biotech (Singapore), received lyophilized, dissolved in PBS, and stored at −20°C.

### 4.2 Expression, Purification and Characterization of Nanobodies

Nanobod‐SpyCatcher fusion proteins were encoded and gene synthesized (Twist Bioscience) into our pJEx411c plasmid backbone, transformed into *E. coli* BL21 (DE3) and starter cultures grown over night from single colonies. 1 l production cultures were inoculated 1:100 in 2×YT medium with 50 mg/l kanamycin and 1% glucose and grown at 37 °C and 250 r.p.m. to OD_600_ ~0.8, then induced with 0.5 mM β‐d‐1‐thiogalactopyranoside and incubated with shaking for 18 h at 25 °C. Cells were harvested by centrifugation for 10 min at 6000 × g and 4 °C, washed once with cold PBS and stored at ‐20 °C. Pellets were thawed on ice and resuspended in lysis buffer (25 mm Tris‐HCl pH7.4, 500 mm NaCl, 10 mm imidazole, 10% glycerol, SigmaFast protease inhibitor, 25 U/ml Benzonase HC (Millipore), and 2 mm DTT) and homogenized with a Cell Disruptor at 20 kPsi (Constant Systems). Lysates were clarified by centrifugation at 85 000 × g for 30 min, the supernatant was filtered through Miracloth tissue (Millipore) and subjected to affinity chromatography on an ÄKTA FPLC (GE Healthcare) using HisTrap HP columns (GE Healthcare) equilibrated into binding buffer (25 mm Tris‐HCl pH7.4, 500 mm NaCl, 10 mm imidazole, 10% glycerol, 2 mm DTT), and elution was performed with a four imidazole steps up to 0.5 m. Fractions were pooled and concentrated using 10K Amicon Ultra (Millipore), followed by gel filtration on a Superdex75 16/600 column (GE Healthcare) into SEC buffer (20 mM HEPES pH7.5, 300 mm NaCl, 10% glycerol and 50 µm EDTA). After spin concentration, aliquots were snap‐frozen in liquid nitrogen and stored at −80 °C. Protein purity and quality were monitored by sodium dodecylsulfate‐polyacrylamide gel electrophoresis (SDS‐PAGE), as well as size‐exclusion chromatography coupled to multi‐angle light scattering (SEC‐MALS) on a DAWN HELEOS II Multi‐Angle static Light Scattering (MALS) detector and OptiLab T‐rEx Refractive Index detector (Wyatt). Protein concentrations were determined on a NanoDrop spectrophotometer by absorbance at 280 nm using sequence‐specific extinction coefficients (https://web.expasy.org/protparam/).

### 4.3 Western Blot

The protein samples were separated on SDS‐PAGE. Meanwhile, PVDF membrane was pre‐wetted in methanol for 30 s, briefly rinsed in deionized water, and then equilibrated in the transfer buffer for 5 min. The proteins were transferred from the gel to the membrane by semi‐dry transfer using the Invitrogen Power Blotter. After protein transfer, the membrane was washed in deionized water 4 times for 5 min each with agitation to remove all transfer buffer. Later, the membrane was incubated with blocking buffer for 60 min at room temperature with agitation. Then, the membrane was incubated with the primary antibody (anti‐His or anti‐Strep) overnight at 4°C. For His‐tagged proteins, the primary antibody was conjugated with horseradish peroxidase (HRP) and thus imaged directly., For Strep‐tagged proteins, the mouse primary antibody was detected withanti‐mouse IgG conjugated with HRP. After incubation in the secondary antibody solution for 1 h with agitation at room temperature, the membrane was washed 3 times with agitation for 5 min each in wash buffer to remove any unbound secondary antibodies. At last, the membrane was incubated with HRP chemiluminescent substrate (Bio‐Rad) for 1 min before being imaged on the ChemiDoc MP Imaging System (Bio‐Rad).

### 4.4 Microscale Thermophoresis (MST)

The RED‐NHS second‐generation dyewas used to label nanobodies according to Monolith Protein Labeling Kit instructions (NanoTemper). Dye‐nanobody‐conjugates were desalted into binding buffer (20 mm HEPES pH 7.4, 150 mm NaCl, 0.05% Tween‐20, 0.02% NaN_3_) using a HiTrap 5 mL desalting column (GE Healthcare). The labeled nanobodies were then concentrated and aliquotes flash‐frozen in liquid nitrogen for storage. On the day of binding analysis, labeled nanobodies were diluted into buffer 2.0 to 5 or 10 nm working concentrations, that yielded between 3000–15000 counts in the Monolith N.T115 PicoRed channel. The LED Power was adjusted to ensure a maximum fluorescence between 300 ‐ 15000 counts. A twofold dilution series of unlabeled target protein was was mixed 1:1 with nanobody solution, incubated for 1 h in the dark and measured in standard capillaries using medium MST power and the data analyzed with MO. Affinity Analysis (x86) based on MST traces and/or initial fluorescence.

### 4.5 OECT and Gate Electrode Fabrication

OECTs were fabricated photolithographically using the perylene‐C (PaC) peel‐off method; detailed fabrication steps can be found in previous work [37]. OECT devices with channel dimensions of width (W) of 10 µm and length (L) of 100 µm were fabricated. p(g_3_C_2_T2‐T) was spin‐cast (800 rpm, 45 s) from a chloroform solution (5 mg/mL) on the substrates to yield a film thickness of about 70 nm in the channel. The second PaC layer was peeled off to pattern the semiconductor and the interconnects. The gate electrodes were fabricated on a glass substrate. A Cr layer with a thickness of 10 nm as an adhesion promoter and a gold layer with a thickness of 100 nm were sputtered on the substrate. First, the Cr layer was sputtered with a 400 W DC power and 25 sccm Ar flow for 15 s, then the Au layer was sputtered with a 400 W DC power and 25 sccm Ar flow for 116 s. After the sputtering, the gate electrode wafer was deposited with a single Parylene‐C layer and then covered with AZ‐10XT photoresist and exposed and developed. The wafer was further etched with Cl_2_ for 10 min to expose the electrode area (2.4 mm diameter circle electrode). The electrodes were cleaned with the KOH and H_2_O_2_ solution (50 mm KOH and 25% H_2_O_2_) for 10 min and then thoroughly rinsed with DI water.

### 4.6 Biofunctionalization of the Gate Electrodes

The gate electrodes were incubated with 0.1 mg/mL cysteine‐terminated SpyTag peptide dissolved in DI water for 1 h. The electrodes were then thoroughly rinsed with DI water. 20 µm RSV, IAV, IBV, or GFP Nb‐SpyCatcher fusion protein solution dissolved in “buffer 2.0” (20 mm HEPES pH 7.4, 150 mm NaCl, 0.02% w/v NaN_3_, 0.05% v/v Tween‐20, 0.1% w/v BSA) was dropped on top of the peptide‐functionalized electrode and left for 1 h. After rinsing with buffer 2.0 and 1× PBS, the nanobody‐functionalized gate electrodes were stored overnight in a 10 nm Lysozyme solution in 1× PBS at 4°C before use.

### 4.7 Electrochemical Measurements

The electrochemical characteristics of gold electrodes were investigated before and after the surface modifications with different reagents by electrochemical impedance spectroscopy (EIS). All electrochemical measurements using gate electrodes addressed as working electrodes were performed using a conventional three‐electrode setup and a potentiostat (Autolab PGstat128N, MetroOhm). A platinum wire and Ag/AgCl electrodes were employed as the counter and reference electrodes, respectively. Measurements were carried out in 5 mL of 1× PBS solution (pH 7.4) containing 10 mm [Fe(CN)_6_]^3−/4−^. Impedance spectra were recorded at a DC voltage of 0 V versus OCP and an AC modulation of 10 mV over a frequency range of 0.1–100000 Hz. The open circuit potential (OCP) measurements were done by connecting the electrodes to the working electrode site. A leakless Ag/AgCl electrode was connected to the reference electrode and the counter electrode site.

### 4.8 X‐Ray Photoelectron Spectroscopy (XPS)

XPS measurements were conducted using an AMICUS/ESCA system with a monochromatic Al Kα source (photon energy: 1468.6 eV). The X‐ray source was operated at 10 kV and 10 mA, corresponding to a power output of 100 W. During the measurements, the analysis chamber was maintained under high vacuum conditions (∼10^−^^7^ Pa). Spectral calibration was performed with reference to the C 1s peak at 284.8 eV.

### 4.9 Kelvin Probe Force Microscopy (KPFM)

KPFM measurements were conducted using a Dimension Icon SPM (Bruker) with a SCM‐PIT‐V2 tip (Bruker). The surface potential, as determined by the contact potential difference (*V*_CPD_), reflects the difference in work function between the sample and the KPFM tip. The equation below offers insights into the surface energy band structure of the surfaces [38]:

$$\;V_{\mathrm{CPD}}=\frac{\phi_{\mathrm{tip}}-\phi_{\mathrm{sample}}}{-e}$$

where $\phi_{tip}$ and $\phi_{sample}$ are the work functions of the tip and sample, and e is the electronic charge.

The sensor was first incubated with 1× PBS and then rinsed with 0.001× PBS and dried and tested by the KPFM. Then, the same sensor was incubated with 10 nm nontarget protein (in 1× PBS) and rinsed with 0.001× PBS and dried, and tested by the KPFM. Finally, the sensor was incubated with 10 nm target protein (in 1× PBS) and rinsed with 0.001× PBS and dried, and tested by the KPFM.

### 4.10 Quartz Crystal Microbalance with Dissipation (QCM‐D) Monitoring

QCM‐D measurements were conducted using a Q‐sense analyzer (QE401, Biolin Scientific). The piezoelectrically active gold sensors were used to mimic the gold gate electrode surface. First, the QCM‐D signals, i.e., the change in frequency (∆*f*) and dissipation (∆*D*), were stabilized in DI water. Then, the peptide solution (0.1 mg/mL SpyTag peptide in water) was injected into the chamber at a 100 µL/min flow rate controlled by a peristaltic pump. When the sensor was fully covered with the solution, the pump was stopped for an hour, and then the sensor surface was rinsed with DI water for 5 min. The same procedure was followed to treat the surface with SpyCatcher‐linked nanobody solution (20 µm in the binding buffer). All QCM‐D data presented in this work belong to the seventh overtone. To quantify the mass accumulating on the sensor (∆*m*), we used the Sauerbrey equation: [39]

$$\Delta m=C\frac{\Delta f}{n}$$

where *n* is the overtone number selected for the calculations and *C* = −17.7 is a constant calculated based on the resonant frequency, active area, density, and shear modulus of the quartz crystal sensor. The mass of the molecules bound on the sensor surface was then estimated using their molecular weight, and the density was calculated considering the sensor surface area (0.7854 cm^2^). Mass (and OCP) values were recorded at designated time points to ensure consistent timing following each step, as shown in the associated figures. For the calculation of the SpyTag/peptide density and nanobody occupancy, we first converted the mass per cm^2^ ($\frac{\Delta m}{A}$, where *A* is 0.7854 cm^2^) into the number of immobilized SpyTag/peptide molecules and nanobodies by considering the molecular weight (*M*_w_) of the peptide molecules (∼1.8 kDa) and nanobody (∼ 28 kDa) and Avogadro's constant (*N*_A_, 6.02 × 10^23^).

The resulting surface density (molecules·cm^−2^) was calculated using the following equation:

$$Density=\frac{\frac{\Delta m}{A}}{M_{w}}\;\times N_{A}$$

And the final occupancy was calculated with the following equation:

$$Occupancy\;\left(\%\right)=\frac{Density_{nanobody}}{Density_{SpyTag}}$$

The eQCM‐D module was used to characterize the nanobody‐target binding events. By connecting the working electrode (QCM‐D sensor), reference, and counter electrode (Figure 3a), OCP signals were monitored in real time during the measurement. After the nanobody biofunctionalization above was finished, 1× PBS was pumped into the chamber as the blank. After the signal was stabilized, 0.001× PBS was pumped into the chamber as the washing buffer to mimic the environment in real OECT sensing. The signal after 0.001× PBS rinsing was considered the blank signal. Then, a nonspecific target solution (in 1× PBS) was pumped into the chamber, followed by 0.001× PBS rinsing, to get the corresponding nonspecific signal. Finally, a specific target solution (in 1× PBS) was pumped into the chamber, followed by 0.001× PBS washing, to get the specific binding signal.

### 4.11 OECT Characterization and Sensor Operation

Electrical characterization of the transistor was carried out with a Keithley source meter, which was used to apply the operating voltages while the source electrode functioned as the common ground in both circuits. All the measurements were conducted under ambient conditions. A PDMS well was glued on top of the channels and filled with 3 mL of 0.001× PBS as electrolyte. The steady‐state measurements of the OECTs were performed by acquiring drain current (*I*_ds_) vs. drain voltage (*V*_ds_) at gate voltages (*V*_gs_) varying between 0.2 V and −0.3 V (step: 0.05 V). *V*_ds_ were swept from 0 to −0.3 V. The sensors were taken out from the storage buffer and first washed with 1× PBS to remove the residue from the storage buffer. Then the sensors were incubated with 1× PBS as the blank. After 15 min incubation, the sensors were washed with 0.001× PBS, which is the same as the OECT electrolyte (to avoid the contamination of the electrolyte). In all our sensor measurements (with electrodes or OECTs), target binding during incubation occurred in physiological media, while measurements were done in dilute electrolyte. Then the sensors were immersed in the PDMS chamber mentioned above, and the corresponding transfer curves were recorded as blank (*I*_0_). The same sensors were then incubated at room temperature for 15 min (pipetting in and out every 5 min) with the protein target solution with nominal concentrations ranging from low am to 1 nm. For each sensor, the incubation with the protein target started from the most dilute one. After incubation, the sensors were washed thoroughly with 0.001× PBS to remove unbound proteins and then immersed in the electrolyte to acquire *I*_ds_ at a given concentration. The normalized response (NR) was used to determine a calibration curve according to the following equation:

$$NR=\left|\frac{I_{ds}-I_{0}}{I_{0}}\right|$$

where *I*_ds_ is the current response of the sensor to an analyte solution that the sensor was exposed to. The limit of detection (LOD) was calculated as the concentration leading to a response that equals the average of the noise level plus three times the noise standard deviation: [40]

$$LOD=\frac{\left(\frac{\Delta I}{I_{0}}+3\sigma \right)-a}{b}$$

where $\frac{\Delta I}{I_{0}}$ is the average response of the GFP negative control sensor, *σ* is the relative standard deviation, and a and b are the intercept and slope of the calibration curve, respectively. The level of noise $\left(\frac{\Delta I}{I_{0}}+3\sigma \right)$ is thus the relative current variation of negative control sensors. The independent samples *t*‐tests were conducted to compare the means of normalization responses between the sensors (RSV, IAV, and IBV sensors) andcontrols (GFP). A two‐tailed *t*‐test with a significance level of *p* = 0.05 was performed. All analyses were carried out using Excel. Inter‐chip coefficient of variation (CV) was calculated with the equation:

$$CV=\frac{\sigma }{\mu }$$

where μ is the average NR of the sensor; σ is the corresponding relative standard deviation.

### 4.12 Saliva Collection

Saliva was collected under controlled laboratory conditions under approved protocols (21IBEC040 and 21IBEC043) sanctioned by the Institutional Biosafety and Bioethics Committee (IBEC) at King Abdullah University of Science and Technology (KAUST). The samples were filtered through a 0.22 µm filter to remove any particulate matter and subsequently diluted with a conditioning buffer (buffer 6.1; 50 mm Tris, 250 mm NaCl, 1% Nonidet P‐40, 0.02% NaN_3_, 0.5% BSA, and 4× complete) at a 1:3 volume‐to‐volume ratio. The diluted saliva was then mixed with a solution containing target proteins (e.g., we added 10 µL of 1 nm target stock solution to 990 µL saliva solution to get 1 mL 10 pm target solution).

### 4.13 Clinical Sample Preparation and Testing

Clinical samples were obtained from human participants under approved protocols (IRB 2021–71, 21IBEC040, and 21IBEC043) sanctioned by the Institutional Review Board of the King Faisal Specialist Hospital and Research Center (KFSHRC) and the Institutional Biosafety and Bioethics Committee (IBEC) at King Abdullah University of Science and Technology (KAUST). Informed consent was obtained from all participants before sample collection. Nasopharyngeal swabs from both virus‐positive and healthy individuals were stored in universal transport medium (UTM) at 4°C and analyzed within 1–2 days of collection. Before testing, clinical samples were thawed for one hour and subsequently mixed with conditioning buffer 6.1 in a 1:3 ratio (500 µL clinical sample with 1500 µL buffer).

## Author Contributions

T. C. performed all electrochemistry, surface characterization, and QCM‐D measurements and analysis, and OECT fabrication and measurements, and wrote the manuscript. Y. R. designed and purified nanobody and target protein constructs, performed SEC‐MALS and MST experiments. A.A. purified and characterized additional nanobody constructs, both under the supervision of R.G. and S.T.A who revised the manuscript. S. W. and L.C. did the XPS and KPFM characterization. Y. W. synthesized the OECT channel material. A. H. and J. P. A. helped with the design and fabrication of the devices. L. S. helped with the OCP measurements and introduced the concept of electrode pretreatment. K. G. helped with the OECT characterization and manuscript writing. A. D. collected clinical samples and performed the qPCR. S. I. coordinated the project, designed the experiments, and wrote and revised the manuscript.

## Conflicts of Interest

The authors declare no conflicts of interest.

## Supporting information




**Supporting File**: advs75621‐sup‐0001‐SuppMat.docx.

## Data Availability

The data that support the findings of this study are available from the corresponding author upon reasonable request.;
