# Supplementary material for: Time‐Multiplexed Organic Electrochemical Transistor for Saliva‐Based Rapid Detection of Viral Proteins
Source: Adv Sci (Weinh). 2026 May 19:e19839. Online ahead of print. doi: 10.1002/advs.202519839 (PMC13336016; doi:10.1002/advs.202519839)
Supplement: Supplementary file 1 — Supporting File: advs75621‐sup‐0001‐SuppMat.docx. [file ADVS-9999-e19839-s001.docx]

Supplementary Information for

**Time-multiplexed Organic Electrochemical Transistor Biosensors for Saliva-Based Rapid Detection of Viral Proteins**

Tianrui Chang^1^, Yuxiang Ren^2^, Shofarul Wustoni^1^, Atheer Alqatari^2^, Adel Hama^1^, Yazhou Wang^1^, Long Chen^3^, Jessica Parrado Agudelo^1^, Luca Salvigni^1^, Keying Guo^1^, Ashraf Dada^4,5^, Stefan T. Arold^2,*^, Raik Grünberg^2,*^, and Sahika Inal^1,*^

^1^ Organic Bioelectronics Laboratory, Biomedical Sciences Division, King Abdullah University of Science and Technology (KAUST), Thuwal 23955-6900, Saudi Arabia.

^2^ Structural Biology and Engineering, Biomedical Sciences Division, KAUST, Thuwal 23955-6900, Saudi Arabia.

^3^ Imaging and Characterization Core Laboratories, KAUST, Thuwal, 23955-6900, Saudi Arabia.

^4^ King Faisal Specialist Hospital & Research Centre - Jeddah Branch | KFSHRC · Department of Pathology and Laboratory Medicine, Jeddah 23433, Saudi Arabia.

^5^ College of Medicine, Al Faisal University, Riyadh 11533, Saudi Arabia.

[sahika.inal@kaust.edu.sa](mailto:sahika.inal@kaust.edu.sa); [raik.gruenberg@kaust.edu.sa](mailto:raik.gruenberg@kaust.edu.sa); stefan.arold@kaust.edu.sa

**Content**

**Figures**

**Figure S1. OECT characterization.**

**Figure S2. Characterization of** **the nanobody fusion protein targeting RSV.**

**Figure S3. Characterization of the nanobody fusion protein targeting NP of H1N1.**

**Figure S4. Characterization of the nanobody fusion protein targeting HA of H1N1.**

**Figure S5. Characterization of the nanobody fusion protein targeting HA of IBV.**

**Figure S6. QCM-D measurement for the characterization of nanobody immobilization.**

**Figure S7. High-resolution XPS spectra.**

**Figure S8. QCM-D and OCP measurements for the characterization of target protein binding.**

**Figure S9. OCP responses of sensors to increasing target concentrations.**

**Figure S10. QCM-D characterization of the GFP nanobody functionalized electrode.**

**Figure S11. OCP response of GFP electrodes to non-target proteins.**

**Figure S12. Monitoring GFP nanobody functionalization of 4 gold electrodes.**

**Figure S13. The effect of pre-treatment with different blockers on the nanobody-immobilized electrodes investigated using QCM-D.**

**Figure S14. The specificity of the GFP nanobody functionalized electrode before and after the treatment.**

**Figure S15. Summary of the pre-treatment experiment.**

**Figure S16. OCP response of GFP nanobody functionalized electrodes to increasing concentrations of non-target proteins.**

**Figure S17. OCP response of IBV sensors to the target after pre-treatment.**

**Figure S18. Nyquist plots and the change in the charge transfer resistance (*R*_ct_) of nanobody-functionalized electrodes during surface functionalization and after target binding.**

**Figure S19.** **Capacitance (*C*_dl_) values extracted from the stepwise EIS characterization of surface functionalization and target binding.**

**Figure S20. Near-LOD characterization of sensors.**

**Figure S21. Cross-reactivity tests of RSV, IAV, and IBV sensors.**

**Figure S22. Random single-point tests for the three sensors.**

**Figure S23. Long-term stability of RSV sensors under different storage conditions.**

**Figure S24. Spike-protein tests of RSV, IAV, and IBV sensors.**

**Figures S25-S28. Transfer curves obtained during RSV clinical sample analysis.**

**Figures S29-S32. Transfer curves obtained during IAV clinical sample analysis.**

**Figures S33-S36. Transfer curves obtained during IBV clinical sample analysis.**

**Figures S37-S41. Transfer curves obtained during virus-free clinical sample analysis.**

**Figure S42. Correlation between OECT sensor response and RT-qPCR CT values.**

**Tables**

**Table S1. Summary of binding analysis between nanobody conjugates and their target proteins.**

**Table S2. △*f* and △D monitored during SpyTag peptide binding, SpyCatcher/nanobody binding and target binding.**

**Table S3. △*f* monitored during SpyTag peptide and SpyCatcher/nanobody functionalization and incubation with blocking molecules.**

**Table S4. △*f* and △OCP of the GFP nanobody functionalized electrodes when exposed to target (GFP) and a non-target protein (IBV).**

**
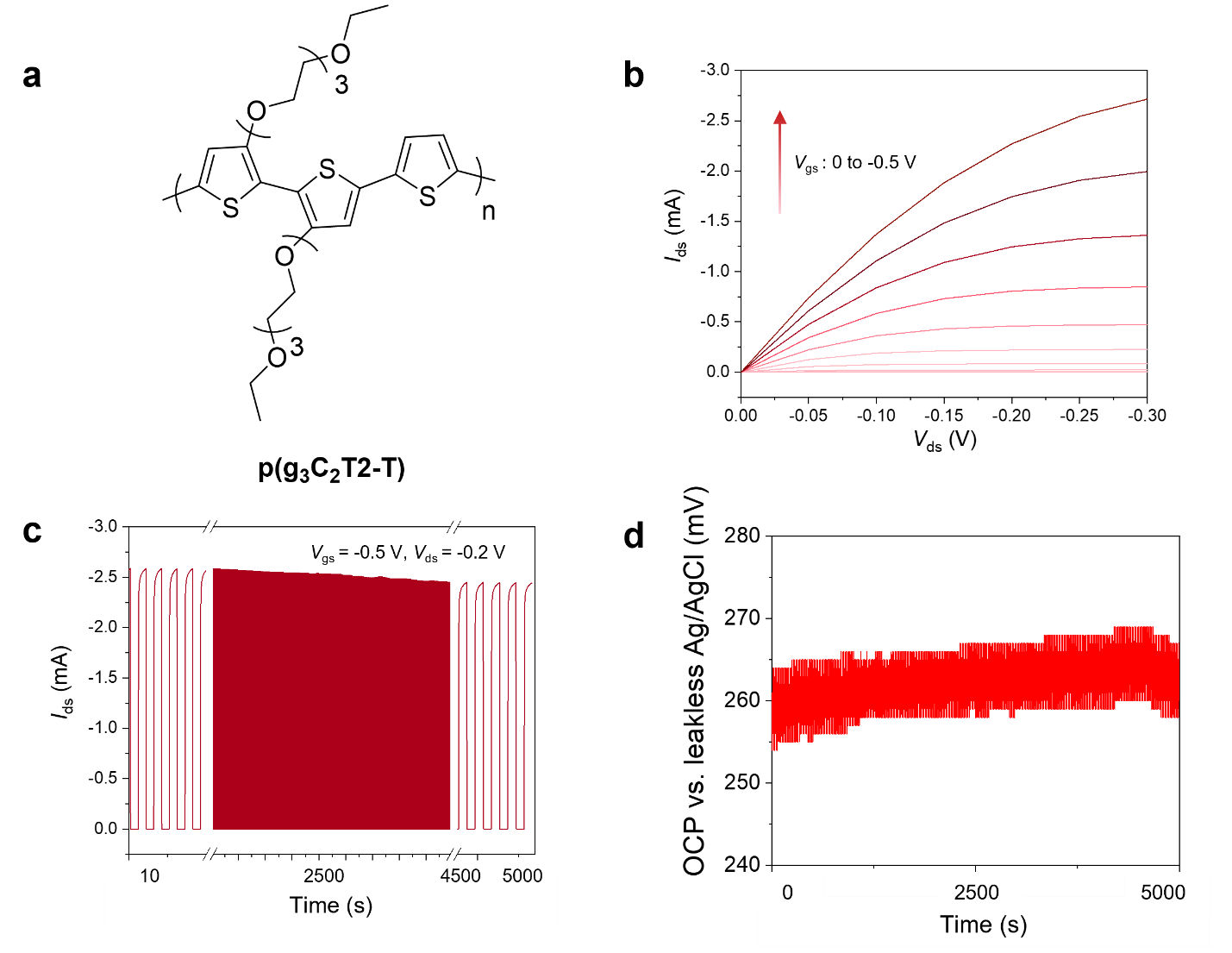
**

**Figure S1. OECT characterization.**  **(a)** The chemical structure of the organic mixed conductor, p(g_3_C_2_T2-T). **(b)** Output curve of the OECT gated with an Au coil in 0.001× PBS. The scan rate for *V*_ds_ and *V*_gs_ was 50 mV/s. **(c)** The OECT channel current (*I*_ds_) monitored during consecutive voltage pulses applied at the gate electrode. *V*_gs_ was toggled between -0.5 V and 0 V with a pulse interval of 2 s over 1000 cycles for 1 hour. The low-voltage operation (*V*_ds_, *V*_gs_ < 0.5 V) suppresses possible parasitic reactions under ambient conditions, enhancing operational stability. **(d)** The open circuit potential (OCP) of the Au coil gate electrode vs. a leakless Ag/AgCl electrode monitored during the OECT measurements shown in **c**. The Au coil has a large capacitance, thus maintaining a stable OCP during device operation. Since it does not need a high concentration of Cl^-^ ions like Ag/AgCl, it is an ideal gate electrode for these measurements.


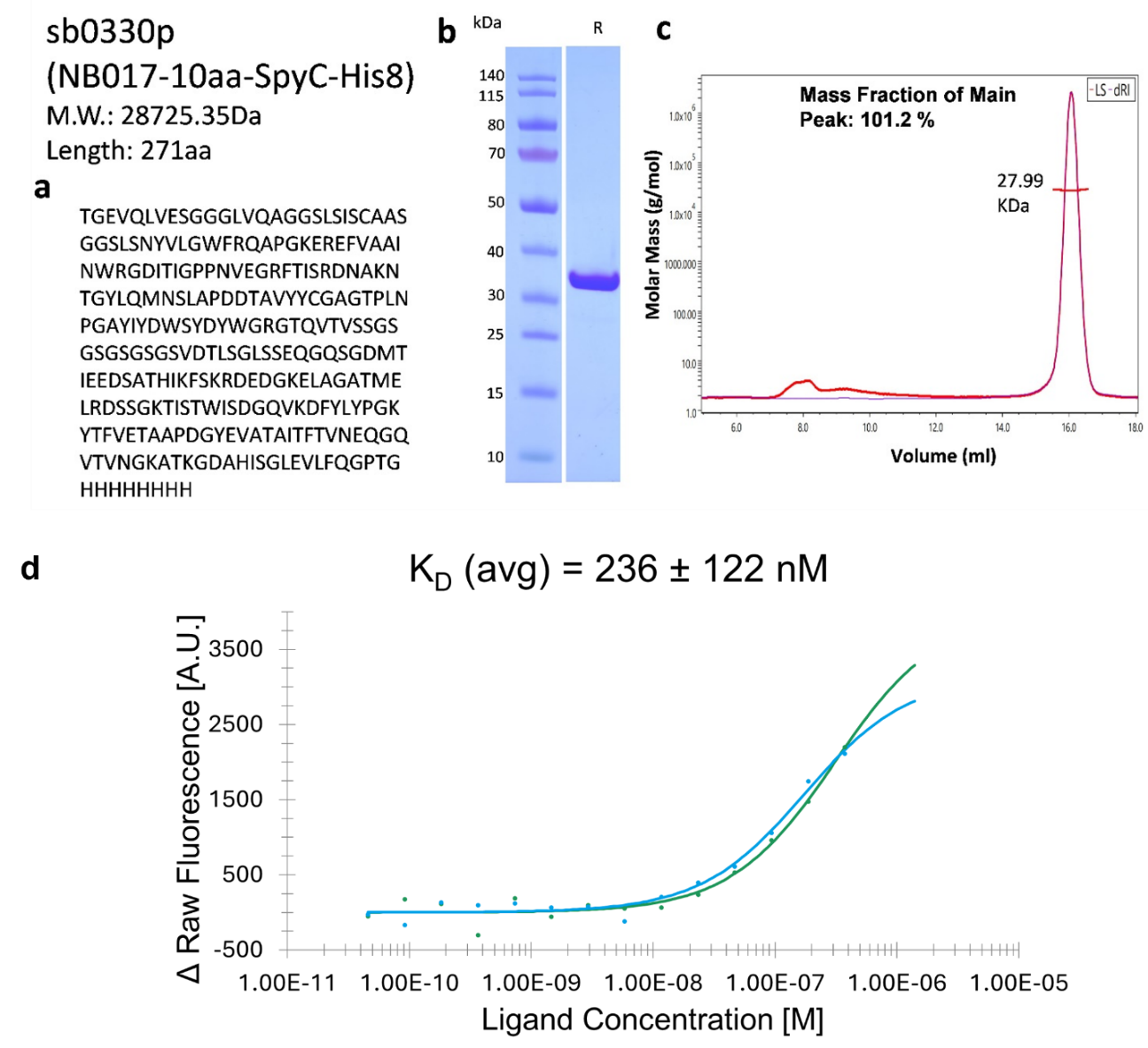


**Figure S2. Characterization of the nanobody targeting RSV fusion protein**. **(a)** Sequence of the nanobody-SpyCatcher fusion with flexible linker and His_8_ tag. **(b)** SDS-PAGE gel under reducing conditions. **(c)** SEC-MALS analysis: the light-scattering (LS) signal in red reports on particle size, the differential refractive index (dRI) signal in purple reports on protein concentration. The measured molecular weight is indicated across the protein peak. Mass fraction calculation: the mass recovered in the protein peak was calculated based on the dRI concentration measurement (sequence-independent) and the apparent molecular weight (determined from LS and dRI) and compared to the mass injected, which was independently determined by 280 nm UV absorbance in conjunction with the sequence-derived extinction coefficient and sequence-derived molecular weight. Minor differences between concentrations determined by UV and dRI, as well as between theoretical and experimentally determined molecular weight and extinction coefficients, can lead to apparent recovery of more than 100% protein mass. As the LS signal is highly sensitive to larger particle size, the presence of an LS but absence of a dRI signal around the column’s exclusion volume (8 mL) indicates the presence of a very small number of molecular aggregates. **(d)** Intrinsic fluorescence binding analysis (IAF) against in-house prepared RSV fusion protein. Data from two replicate measurements were fitted with a sigmoid curve (1:1 binding model).


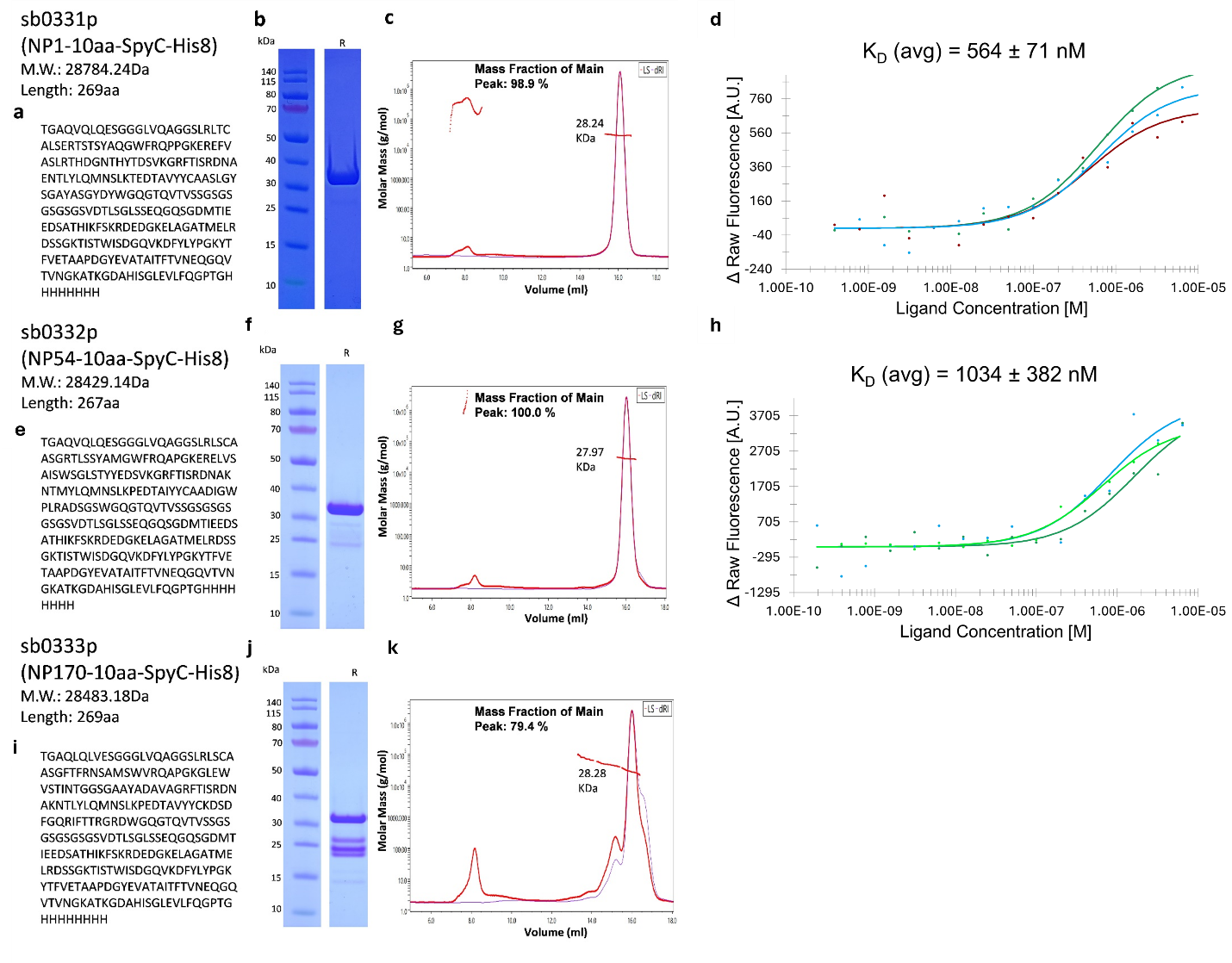


**Figure S3. Characterization of the nanobodies targeting NP of IAV H1N1. (a, e, i)** Sequence. **(b, f, j)** SDS-PAGE gel under reducing conditions. **(c, g, k)** SEC-MALS analysis. **(d, h)** Intrinsic fluorescence binding analysis of three replicates.


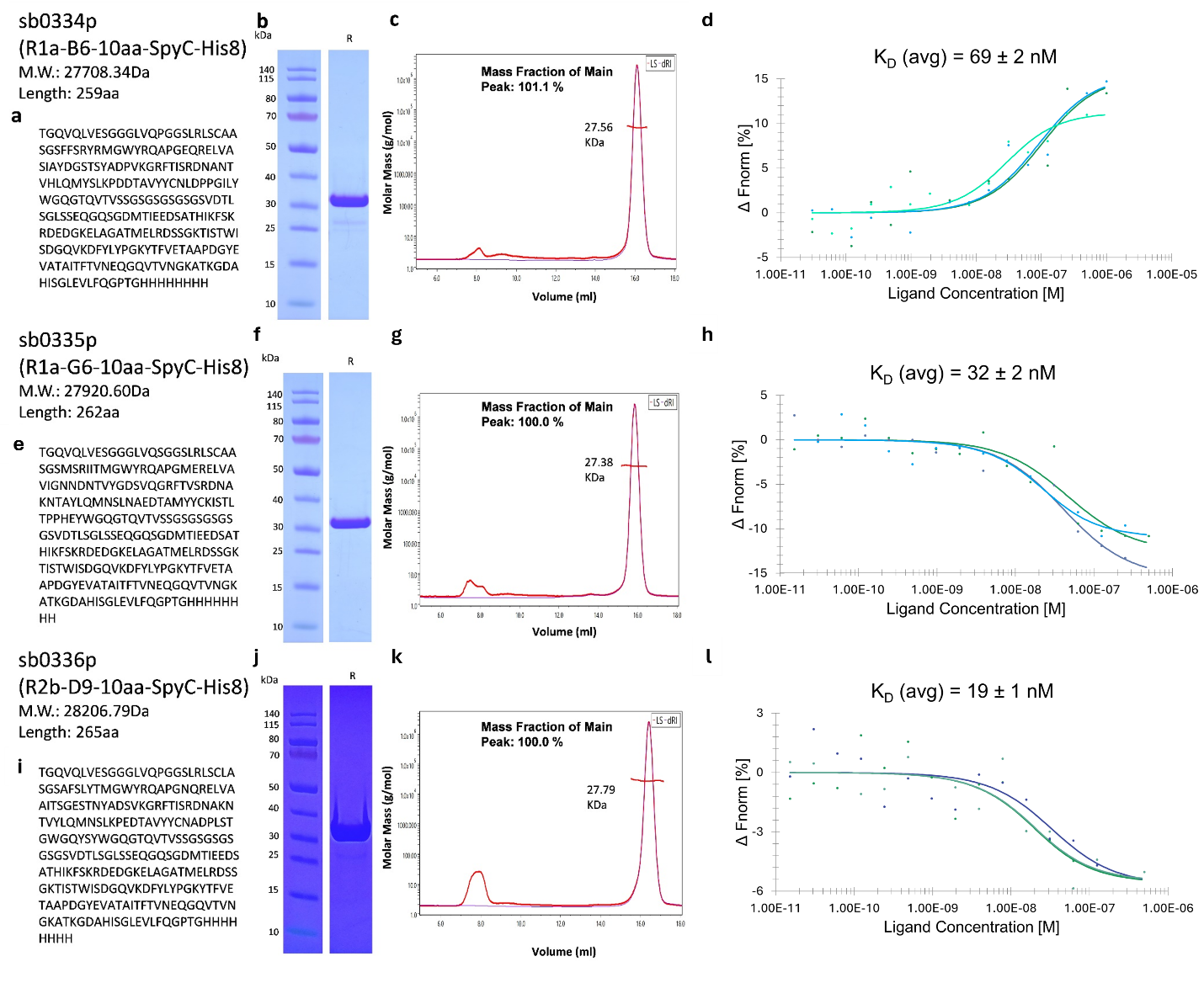


**Figure S4. Characterization of the nanobodies targeting HA1 of IAV H1N1.** **(a, e, i)** Sequence. **(b, f, j)** SDS-PAGE gel under reducing conditions. **(c, g, k)** SEC-MALS analysis. **(d, h, l)** MST binding analysis of two or three replicates.


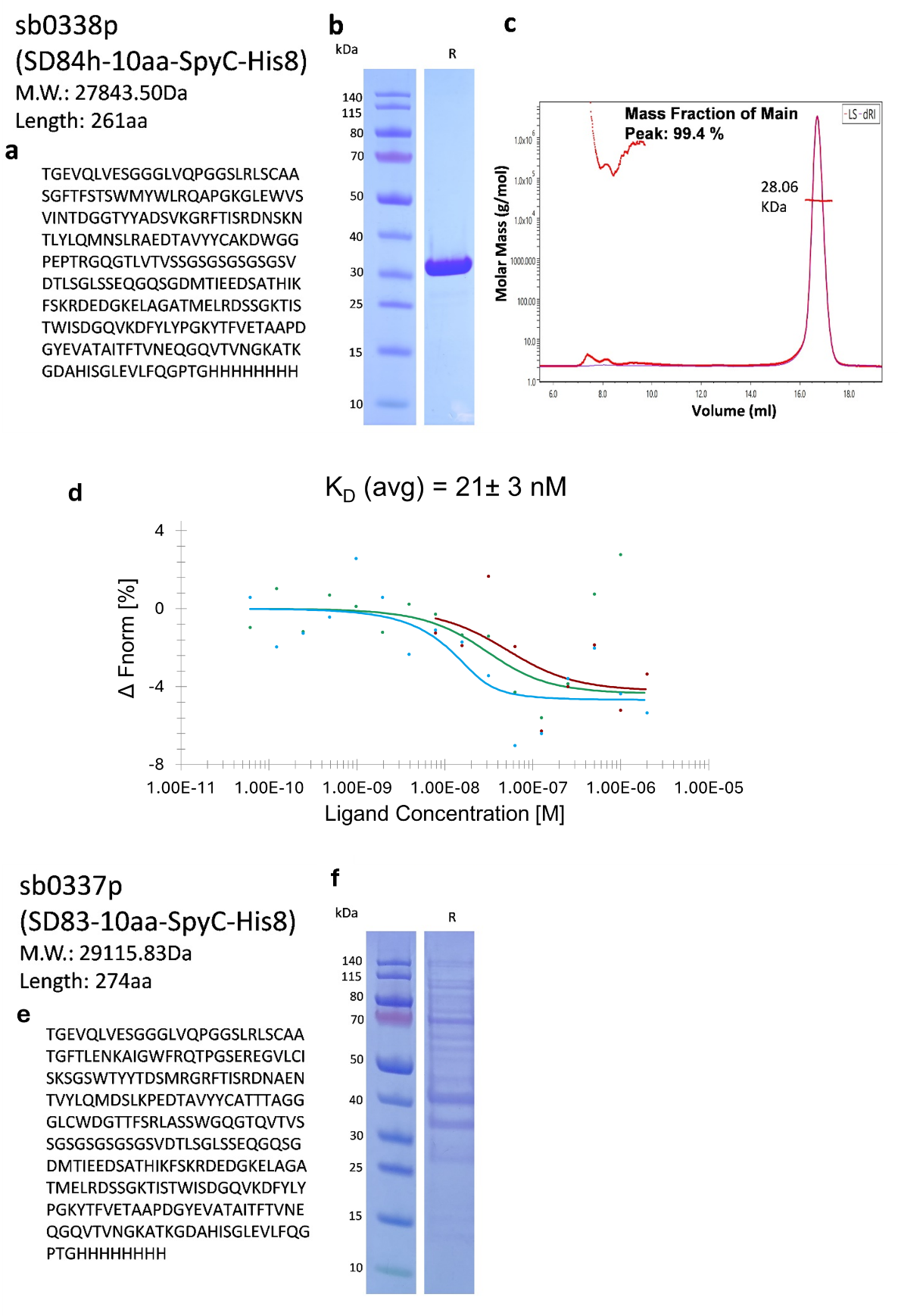


**Figure S5. Characterization of** **the** **nanobodies targeting HA of IBV. (a, e)** Sequence. **(b, f)** SDS-PAGE gel under reducing conditions. **(c)** SEC-MALS analysis. **(d)** MST binding analysis of three replicates is inconclusive owing to low signal over noise, but binding was confirmed using QCM-D experiments. Because of the poor purity of sb0337p, SEC-MALS and MST analysis were not performed.

**
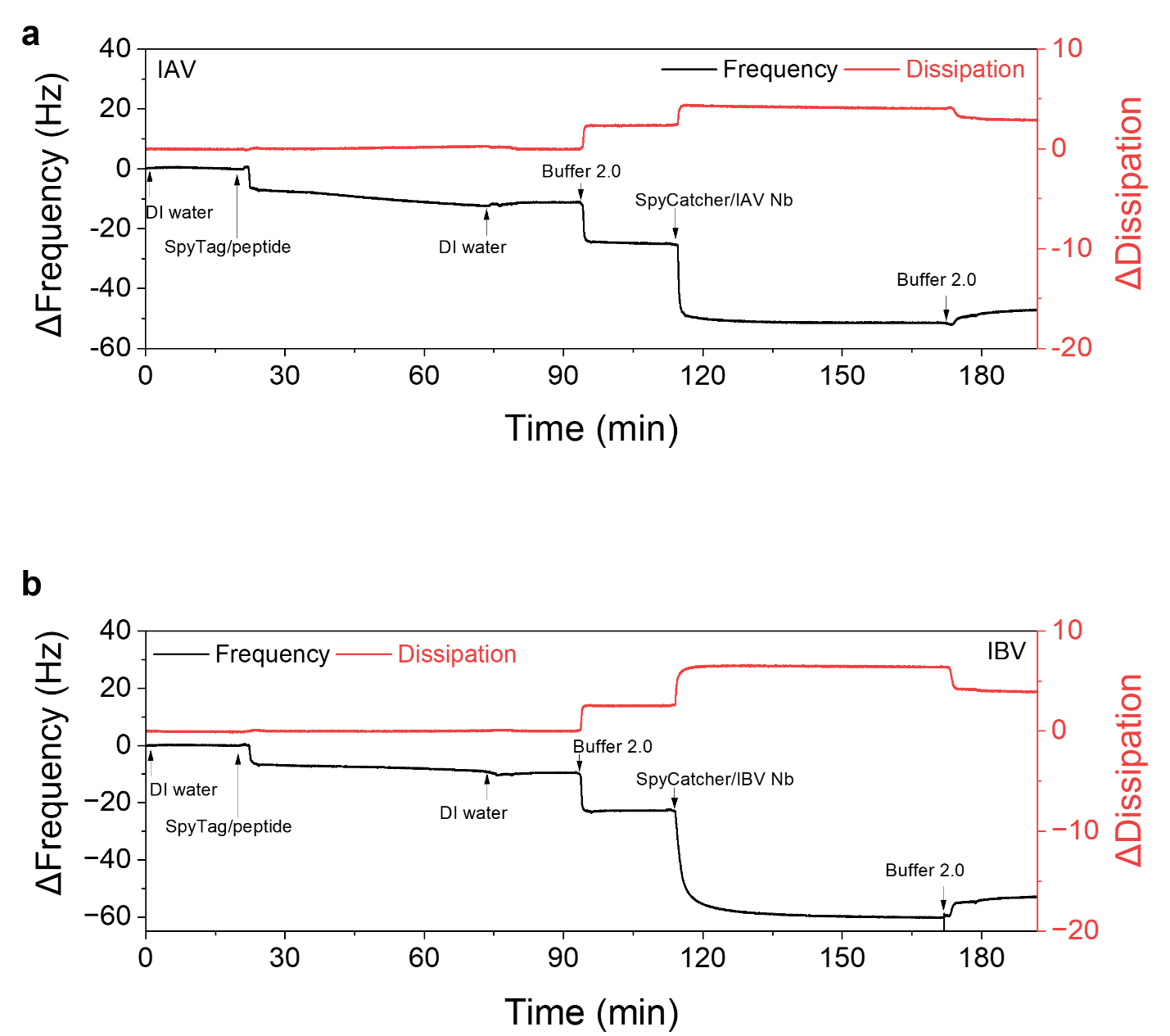
**

**Figure S6. QCM-D measurement for the characterization of nanobody immobilization.** Biofunctionalization of electrodes with **(a)** IAV Nb and **(b)** IBV Nb constructs. The raw data of the 7^th^ overtone is shown.


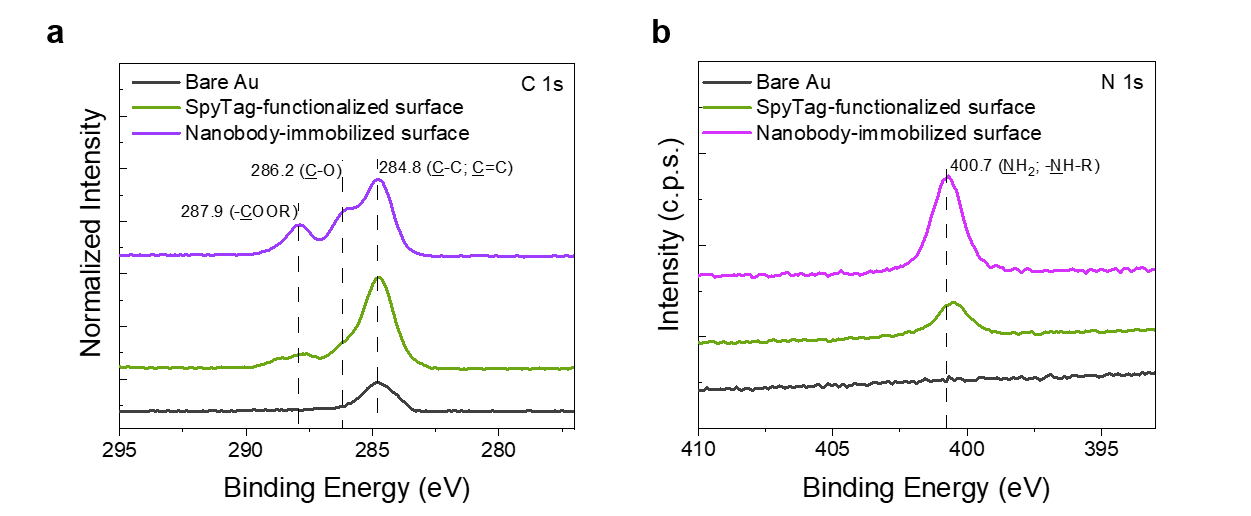


**Figure S7. High-resolution XPS spectra of (a) C 1s and (b) N 1s.** The data were recorded for the Au electrode before and after the immobilization of the peptide and the RSV nanobody construct.


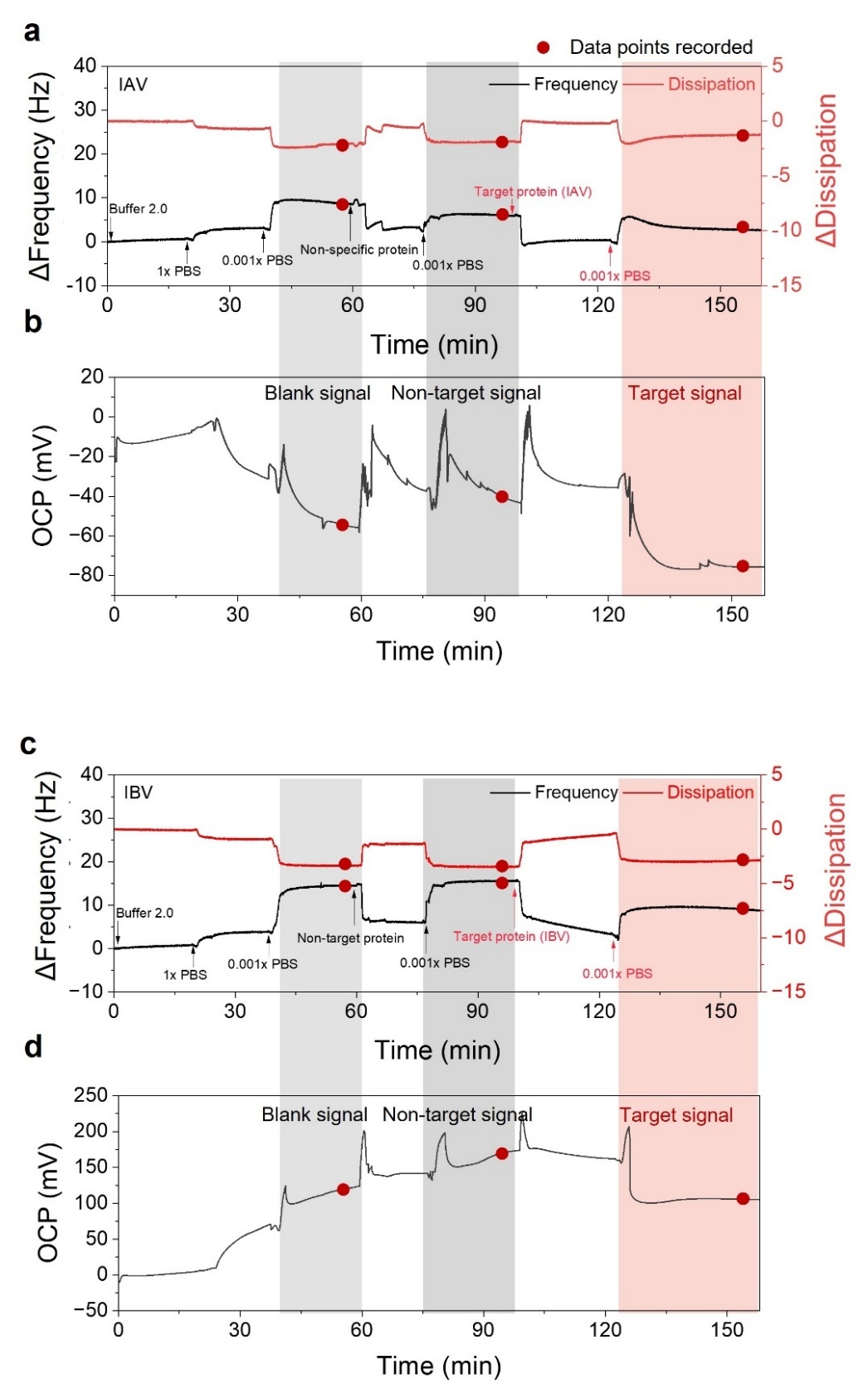


**Figure S8. QCM-D and OCP measurements for the characterization of target protein binding.** **(a-b)** IAV Nb functionalized electrode, and **(c-d)** IBV Nb functionalized electrode. Proteins introduced are dissolved in 1× PBS at a concentration of 100 nM. For **(a-b)**, the non-target protein includes 100 nM IBV & RSV proteins, and the target protein is 100 nM IAV protein. For **(c-d)**, the non-target protein includes 100 nM IAV and RSV proteins, and the target protein is 100 nM IBV.


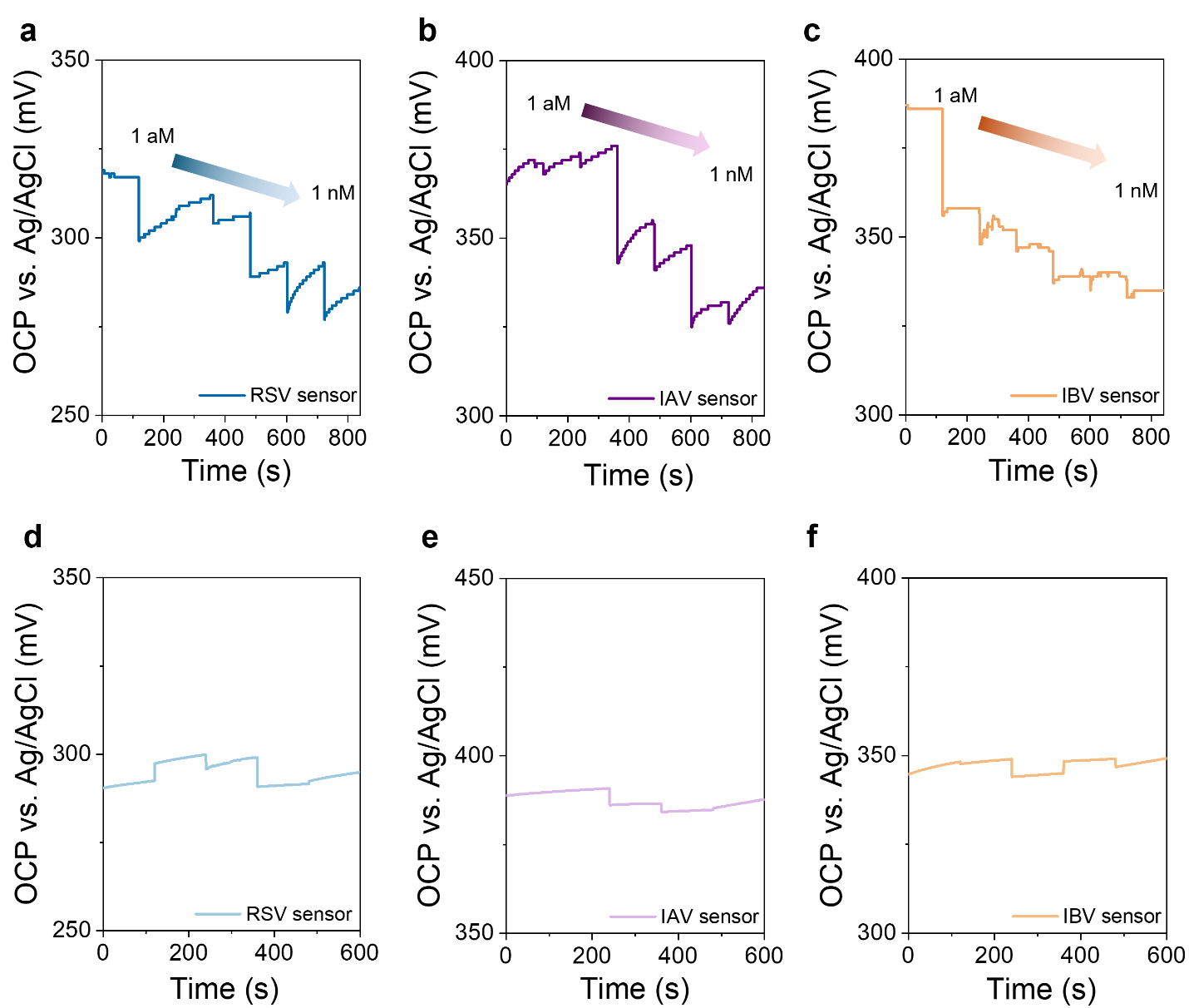


**Figure S9. OCP responses of sensors to increasing target concentrations.** OCP response of **(a)** RSV Nb, **(b)** IAV Nb, and **(c)** IBV Nb functionalized electrodes to different target concentrations (1 aM, 100 aM, 10 fM, 1 pM, 100 pM, and 1 nM). OCP response of **(d)** RSV Nb, **(e)** IAV Nb, and **(f)** IBV-Nb functionalized electrodes to multiple incubations with 1× PBS. During the first 0~120 s, we recorded the blank signal, then the measurement was paused, and the sensors were taken out for incubation with the protein (or protein-free PBS). After the incubation and rinsing steps, the sensors were placed in the measurement electrolyte for the OCP test.


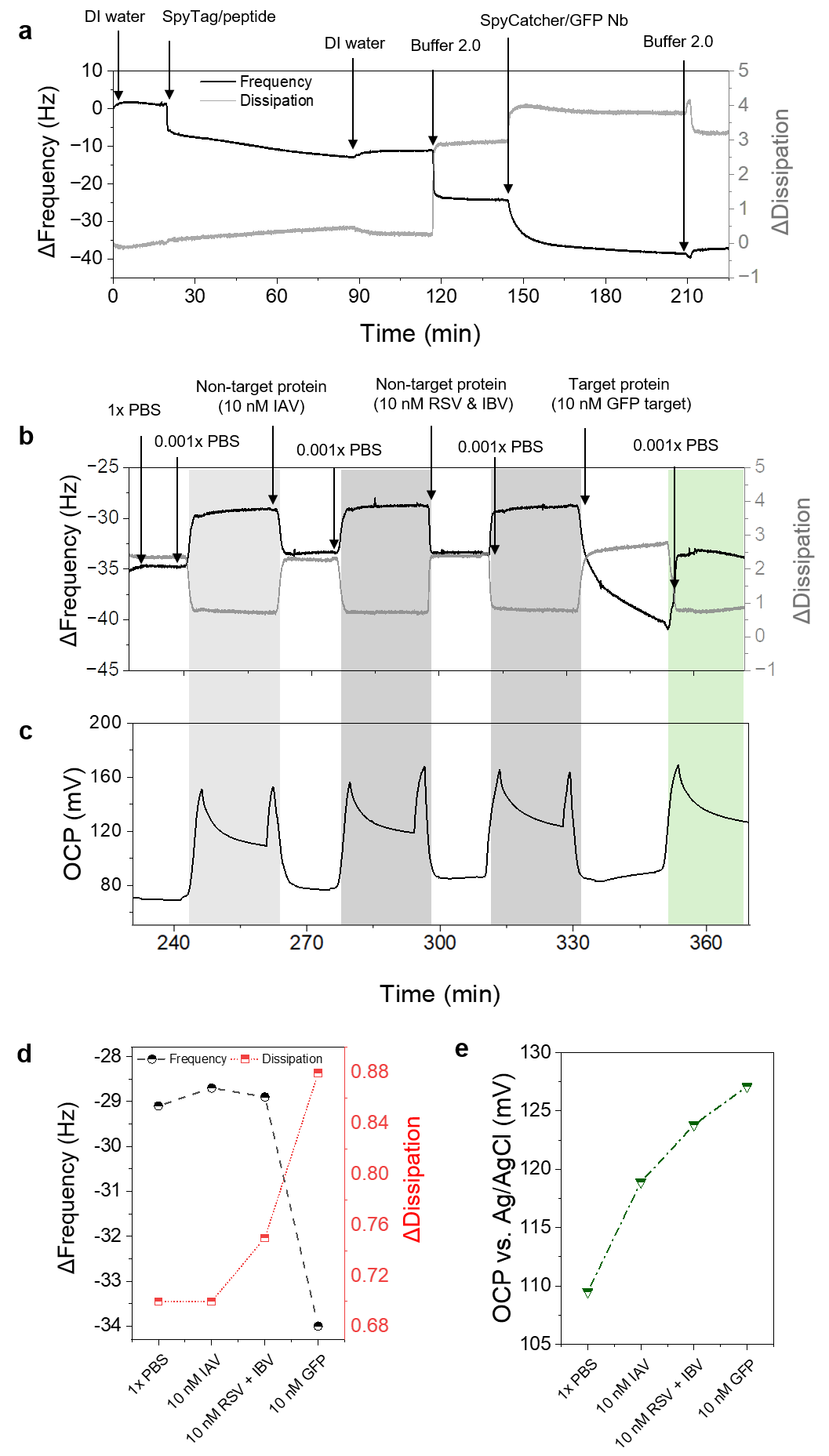


**Figure S10. QCM-D characterization of the GFP nanobody functionalized electrode.** **(a)** QCM-D data for the functionalization of the electrode surface with GFP Nb. **(b)** QCM-D measurements exploring the non-specific interactions of non-target proteins (IAV, IBV, RSV) and the specific interactions of GFP with the nanobody functionalized surface. **(c)** Real-time monitoring of the OCP signal of the GFP Nb functionalized electrode during its exposure to different proteins. The experiment was performed in the EQCM-D setup. **(d)** Frequency and dissipation changes of the GFP Nb functionalized sensor upon its exposure to different proteins. **(e)** OCP change as the GFP Nb functionalized electrode is incubated with different proteins.


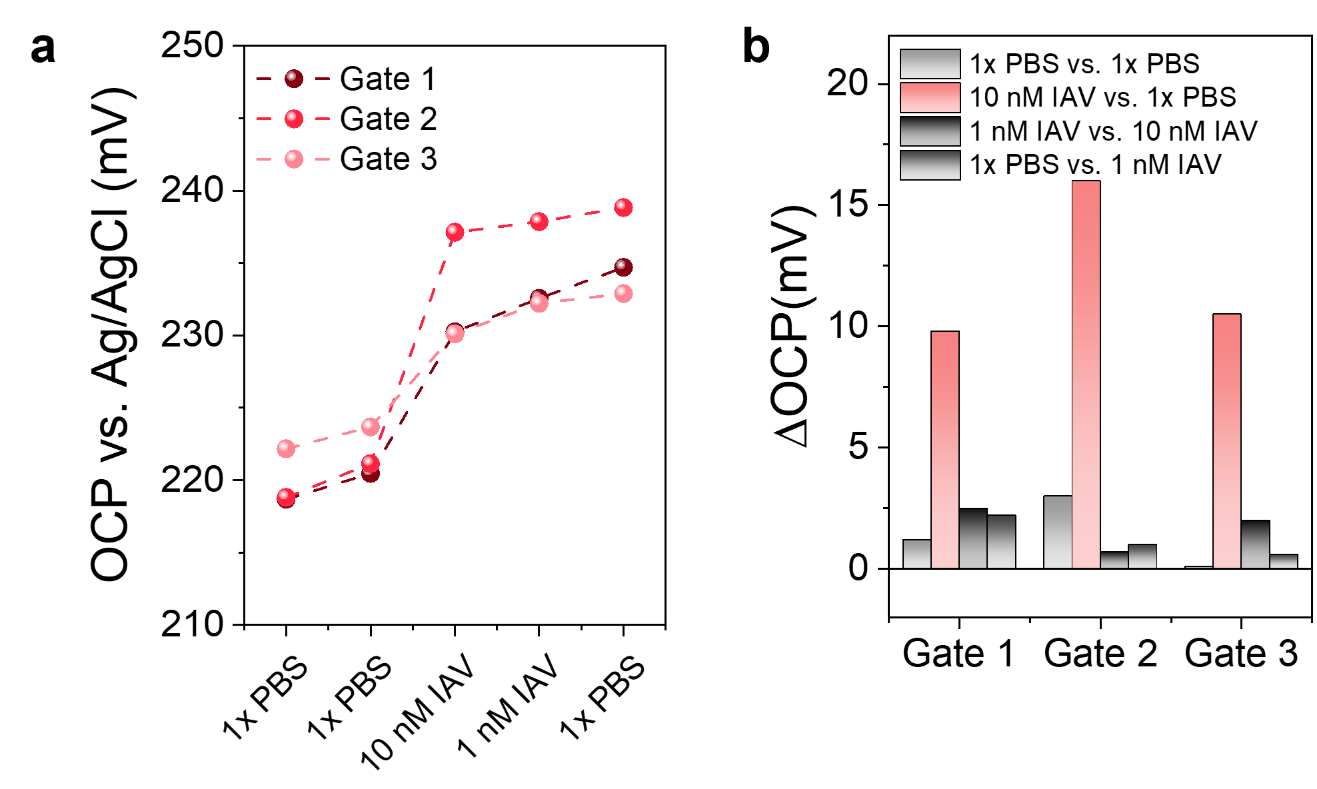


**Figure S11. OCP response of GFP electrodes to non-target proteins.** **(a)** OCP of three electrodes when they are exposed to 1× PBS (1), 1× PBS (2), 10 nM IAV, 1 nM IAV, followed by a final round of 1× PBS (3). **(b)** The changes in OCP between each step. The OCP values changed significantly in response to 10 nM IAV incubation.


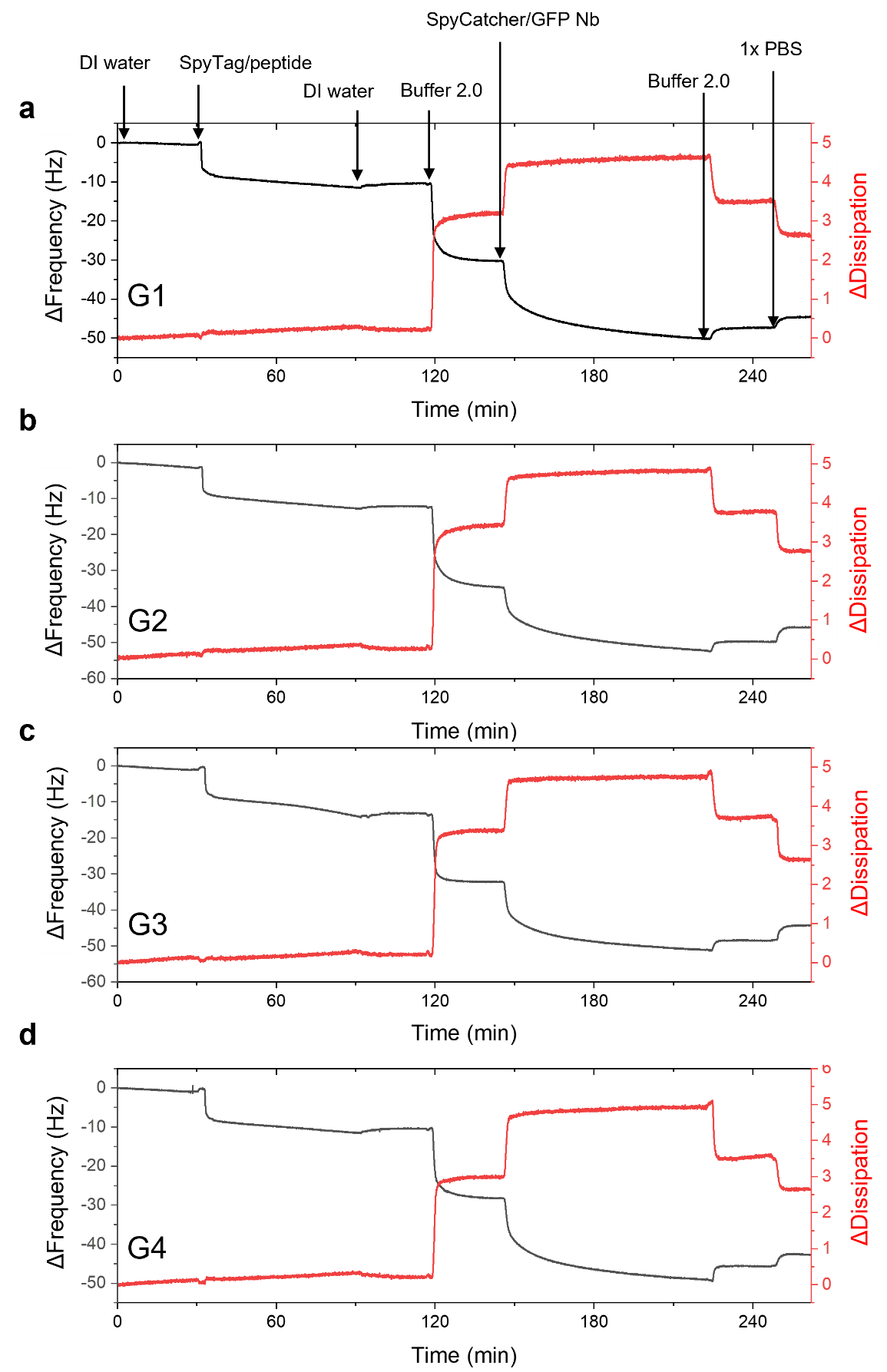


**Figure S12. Monitoring GFP nanobody functionalization of 4 gold electrodes.** These four electrodes will be treated by 1× PBS (**a**, G1), 10 nM lysozyme (**b**, G2), 10 nM casein (**c**, G3), and 10 nM BSA (**d**, G4).


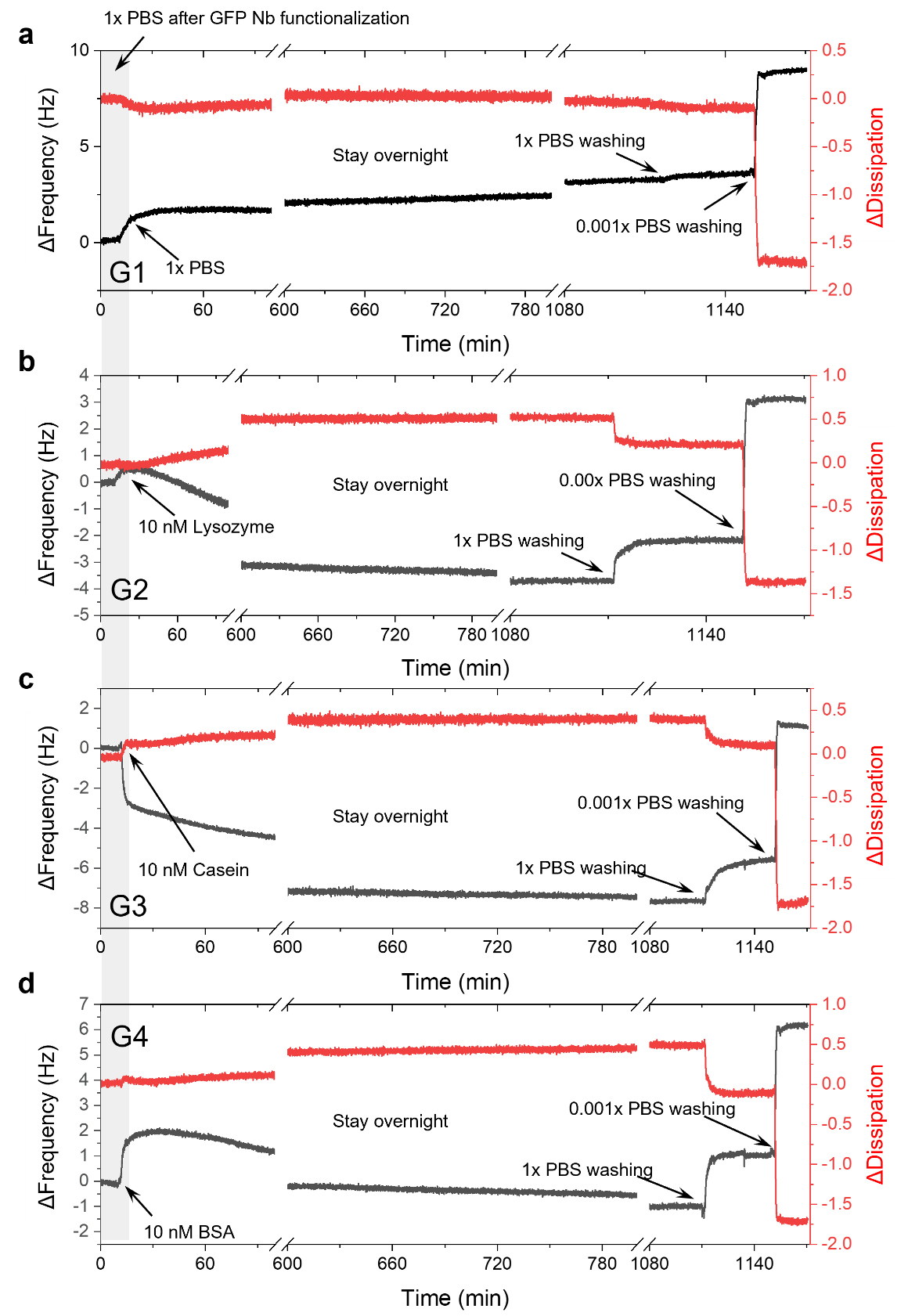


**Figure S13. The effect of pre-treatment with different blockers on the nanobody-immobilized electrodes investigated using QCM-D. (a**) G1 was treated with 1× PBS (no treatment), **(b**) G2 was treated with 10 nM lysozyme, **(c)** G3 was treated with 10 nM casein, and **(d**) G4 was treated with 10 nM BSA. After the introduction of each blocker, the electrodes were kept in the QCM-D chambers overnight and then washed with 1× PBS, followed by washing with 0.001× PBS (to mimic the real OECT operation).


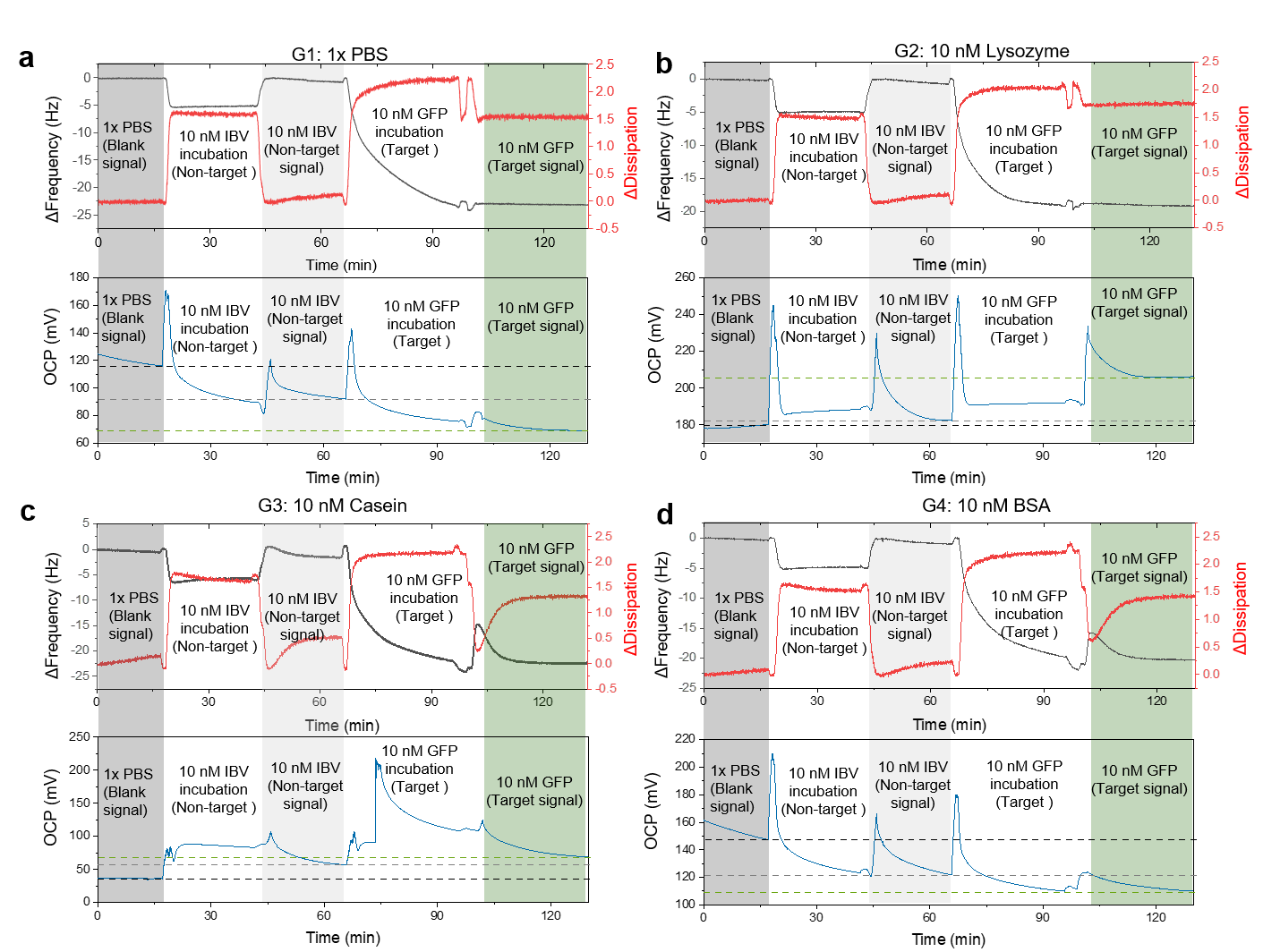


**Figure S14. The specificity of the GFP nanobody functionalized electrode before and after the treatment.** 1× PBS was first introduced to get the baseline signals, then 10 nM IBV protein (non-target) was pumped into the chambers, and finally, 10 nM GFP protein was introduced. OCP was simultaneously measured. **(a)** G1 (PBS, no treatment), **(b)** G2 (lysozyme), **(c)** G3 (casein), and **(d)** G4 (BSA). The shadowed areas show the signals after the incubation with the target GFP protein and rinsing.


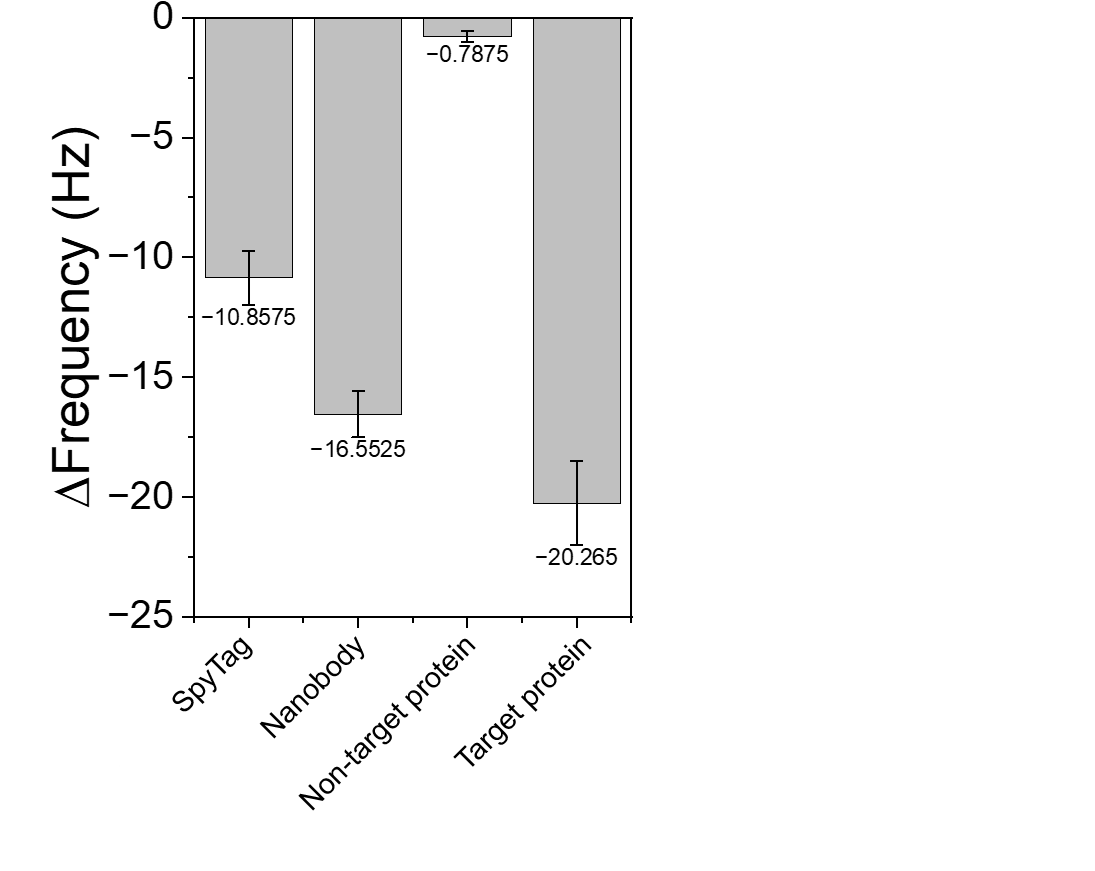


**Figure S15. Summary of the lysozyme pre-treatment experiment.** Average frequency change of four GFP-Nb electrodes after each step. Biofunctionalization shows high consistency, and the pre-treatment step does not affect the specificity of the nanobody towards its target.


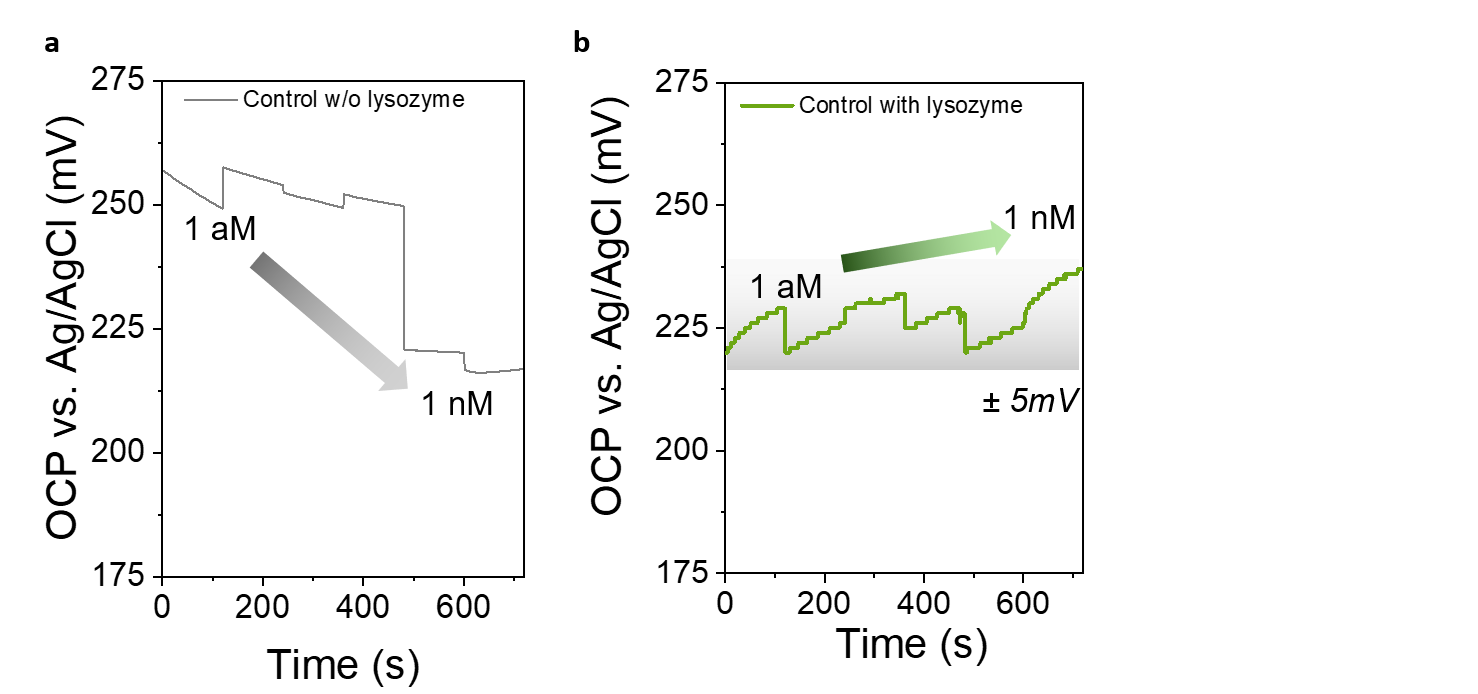


**Figure S16. OCP response of GFP nanobody functionalized electrodes to increasing concentrations of non-target proteins: (a)** without lysozyme treatment **(b)** with lysozyme treatment. During the first 0~120 s, we recorded the blank signal, then the measurement was paused, and the sensors were taken out for incubation with a mixture of non-target proteins. After the incubation and rinsing steps, the sensors were placed in the measurement electrolyte for the OCP test.


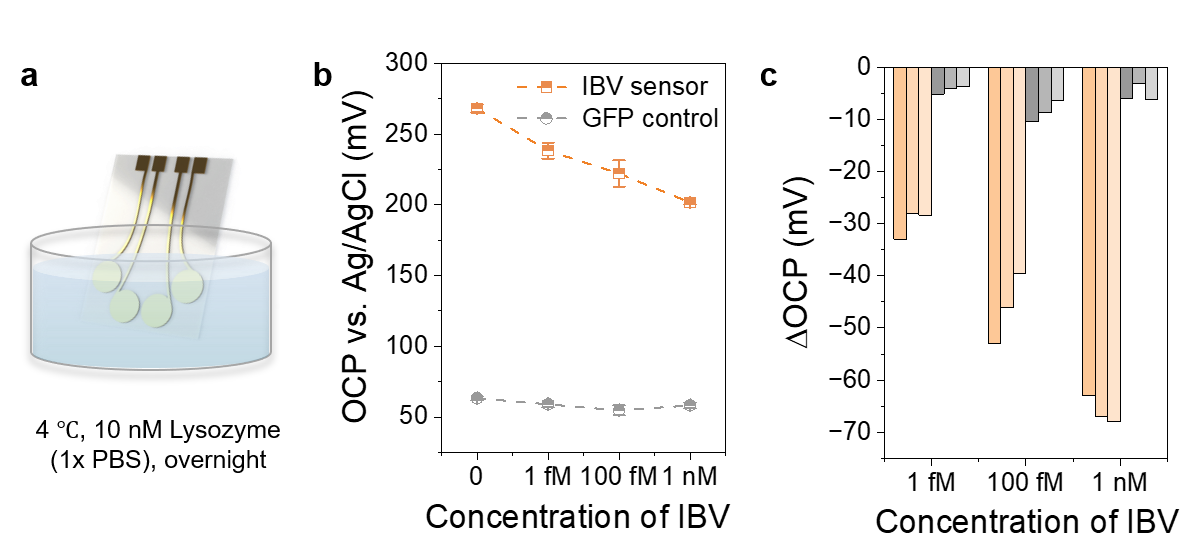


**Figure S17. OCP response of IBV sensors to the target after pre-treatment with lysozyme.** **(a)** Schematic of the lysozyme pre-treatment step: The nanobody functionalized electrodes were kept overnight at 4 °C in lysozyme solution. Electrodes were then washed with 1× PBS before use. **(b)** OCP change of IBV Nb functionalized electrodes and GFP Nb functionalized electrodes after exposure to various concentrations of IBV HA target. Three electrodes were tested for each nanobody type. **(c)** The change in OCP of three IBV Nb functionalized electrodes (orange) and three GFP Nb functionalized electrodes (gray) after incubation with IBV proteins.


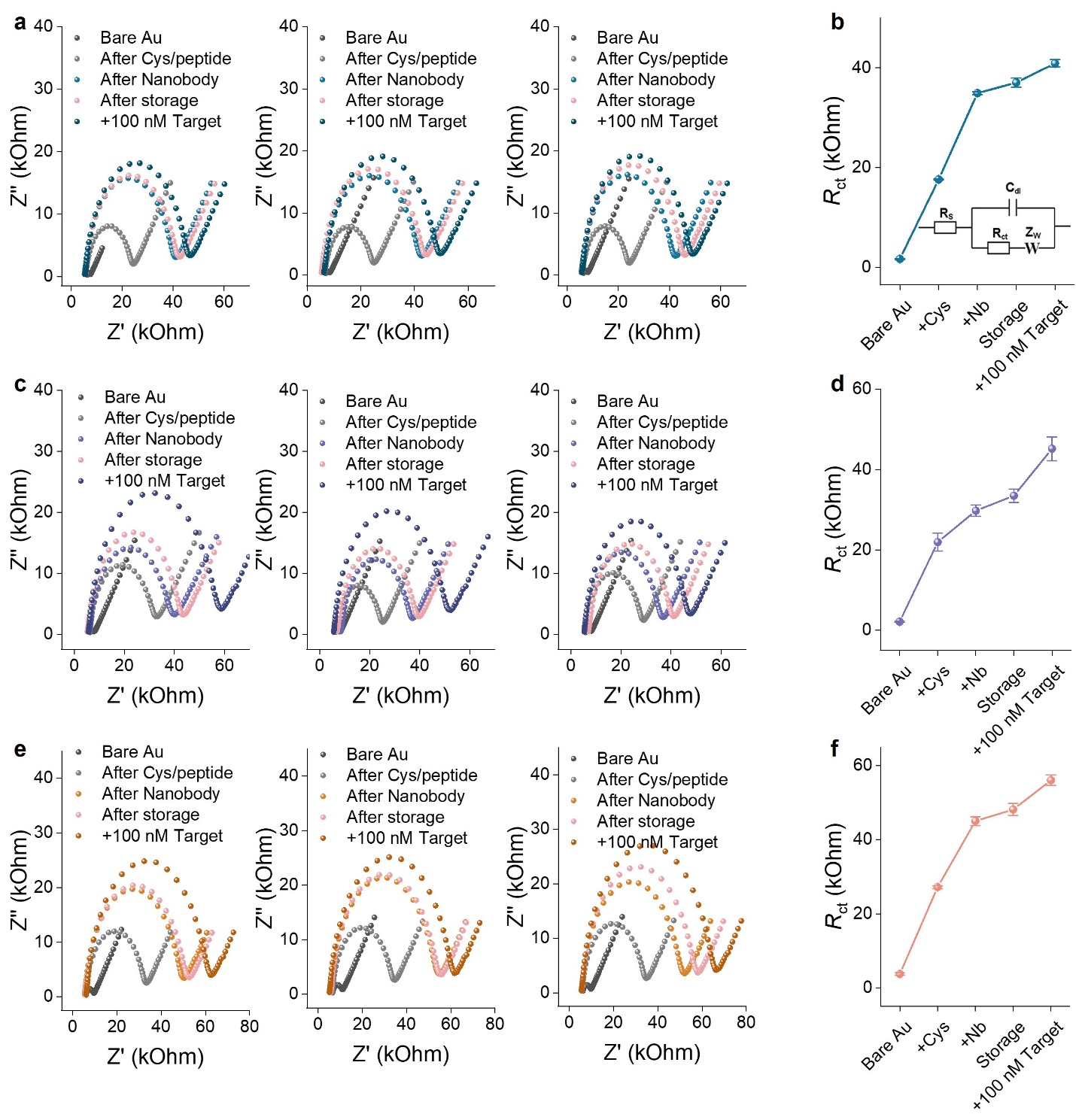


**Figure S18. Nyquist plots and the change in the charge transfer resistance (*R*_ct_) of nanobody-functionalized electrodes during surface functionalization and after target binding.** **(a, c, and e)** Nyquist plots of RSV, IAV and IBV electrodes, respectively at different stages, including bare gold, after SpyTag functionalization, after nanobody functionalization, after overnight treatment with lysozyme, and after target incubation. **(b, d and f)** The evolution of *R*_ct_ of RSV, IAV and IBV electrodes, respectively.


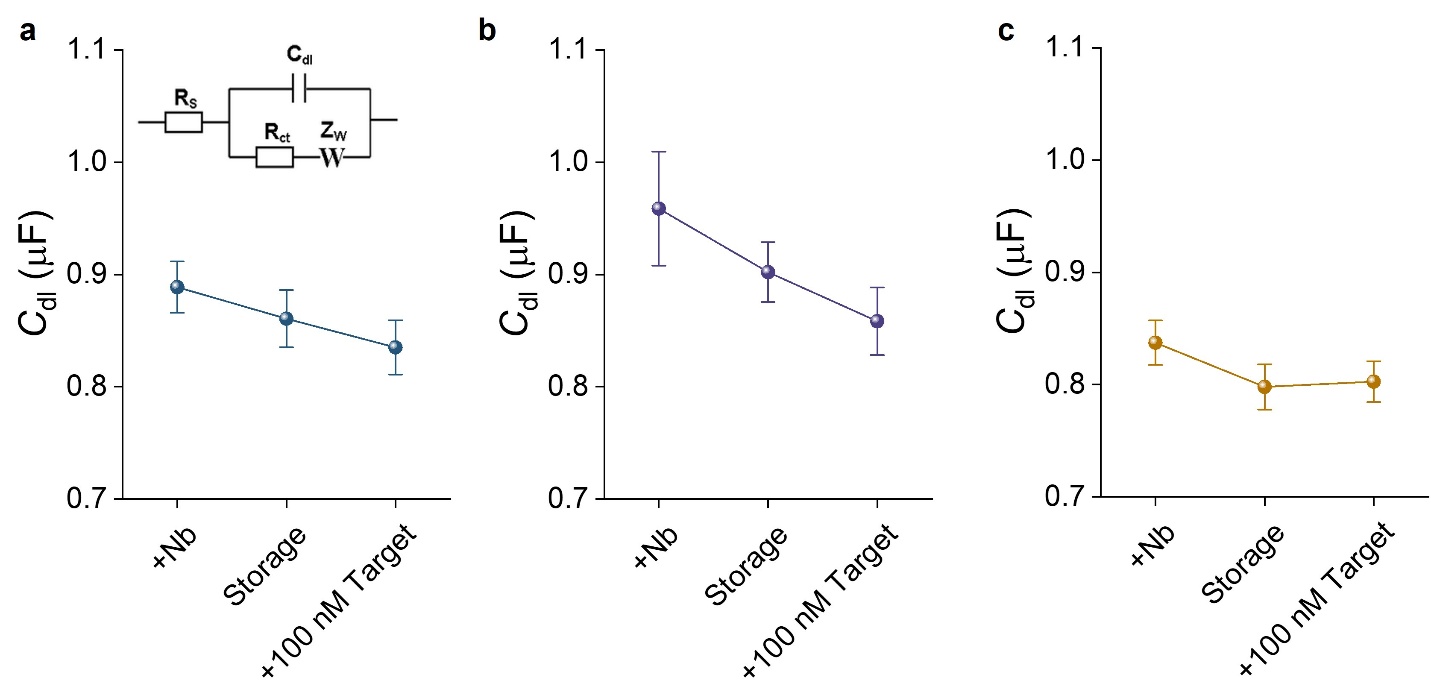


**Figure S19.** **Capacitance (*C*_dl_) values extracted from the stepwise EIS characterization of surface functionalization and target binding.** **(a-c)** The evolution of *C*_dl_ of **(a)** RSV, **(b)** IAV and **(c)** IBV electrodes after nanobody (Nb) immobolization, storage in lyzome over night, and taraget protein exposure.


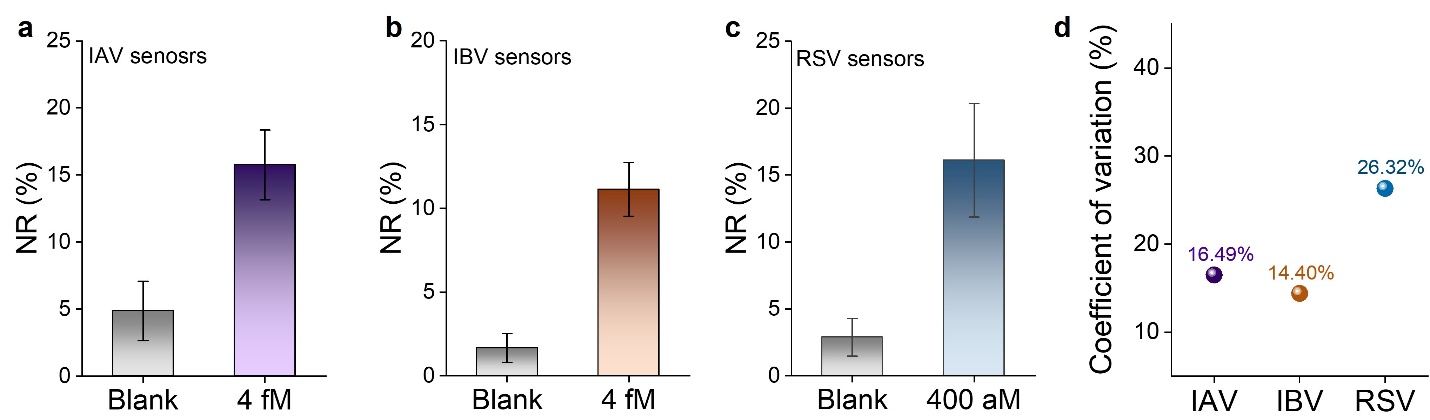


**Figure S20. Near-LOD characterization** of **(a)** IAV sensors, **(b)** IBV sensors, and **(c)** RSV sensors**. The** error bars are derived from 6 independent sensors. **(d)** The inter-chip variation of the three sensors at their near-LOD concentrations. The


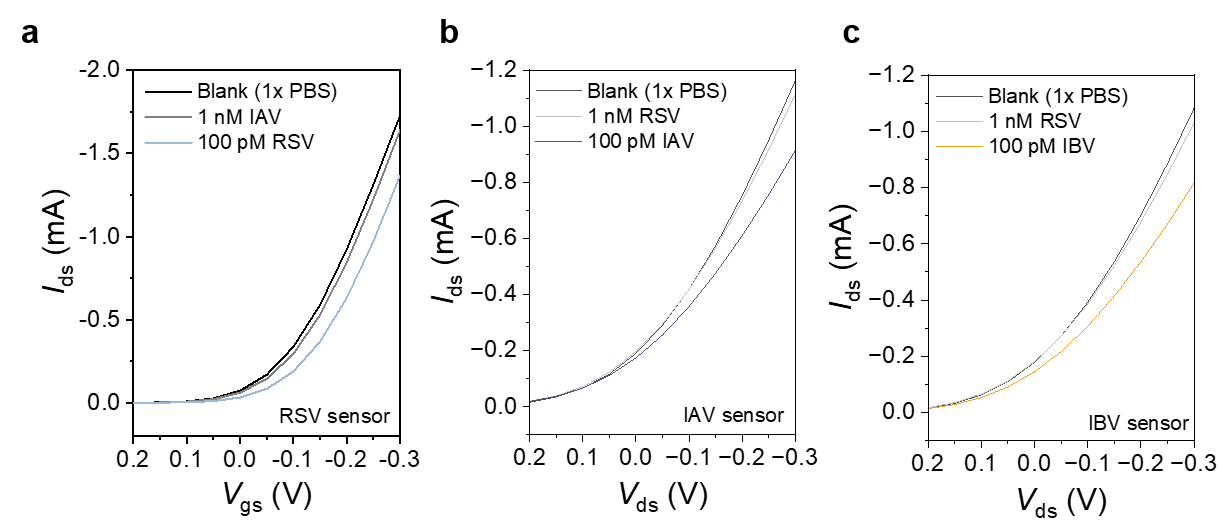


**Figure S21. Cross-reactivity tests of RSV, IAV, and IBV sensors. Cross-reactivity of the (a) RSV sensor, (b) IAV sensor, (c) IBV sensor.** After recording baseline signals, each sensor was first incubated with **1 nM of non-target protein**, and the corresponding **transfer curve** was recorded. Subsequently, the same sensor was incubated with **100 pM of its specific target protein.**


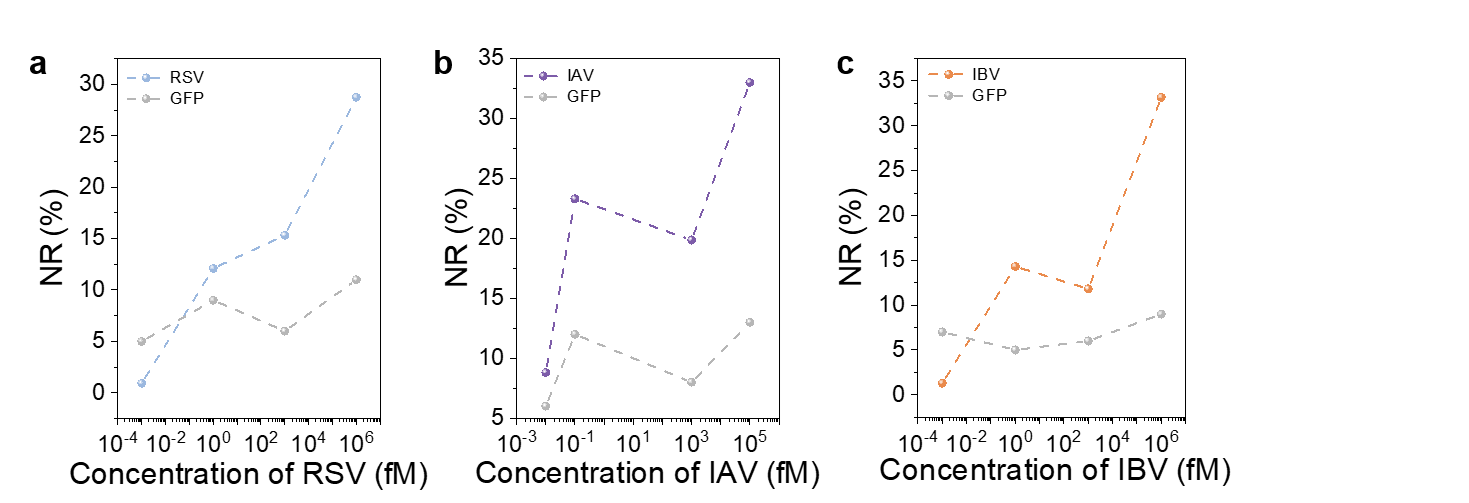


**Figure S22. Random single-point tests for the three sensors.** The NR of **(a) RSV sensor, (b) IAV sensor, (c) IBV sensor for various target concentrations.** In these tests, we prepared four pairs, i.e., 1 sensor and 1 GFP Nb control: in total; 4 RSV sensors, 4 IAV sensors, 4 IBV sensors, and 12 GFP sensors. Each pair of sensors and negative controls were incubated first with 1× PBS and washed with 0.001× PBS to get blank signals. The same sensors were then incubated with one target solution, followed by washing with 0.001× PBS.


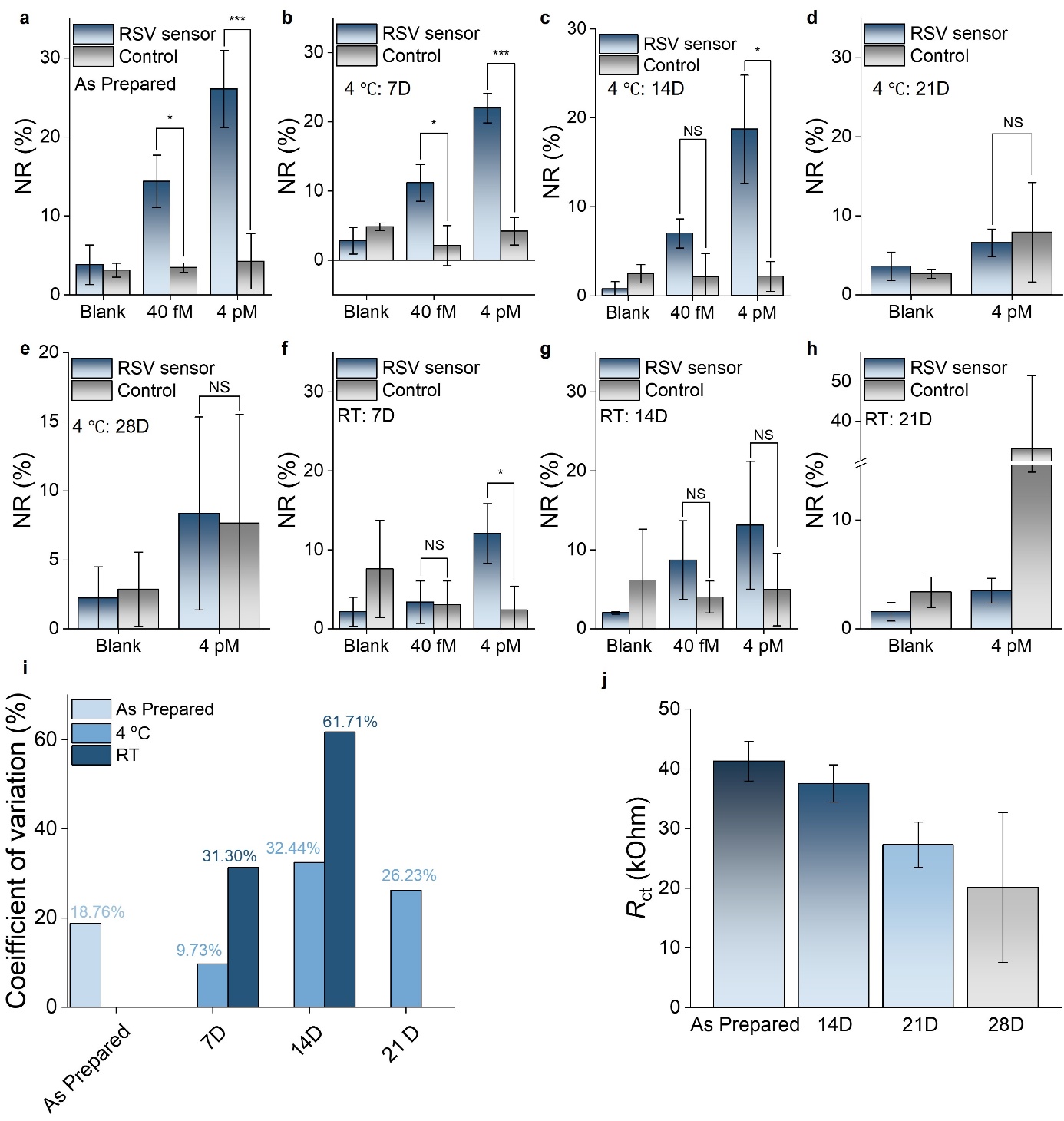


**Figure S23. Long-term stability of RSV sensors under different storage conditions. (a)** As prepared sensors normalized response (NR) with sequential exposure to 1× PBS (baseline), 40 fM and 4 pM targets. **(b–h)** Time-dependent sensing performance of devices stored in 1× PBS at **(b-e)** 4 °C, evaluated at day 7, 14, 21, and 28, and **(f-h)** room temperature, evaluated at day 7, 14, and 21. **(i)** Coefficient of variation (CV) of sensor responses at 4 pM under different storage conditions, highlighting the improved stability and reproducibility of sensors stored at 4 °C. Error bars were calculated from 3 sensors. **(j)** Charge transfer resistance (*R*_ct_) values of RSV sensors stored at 4 °C over several days.


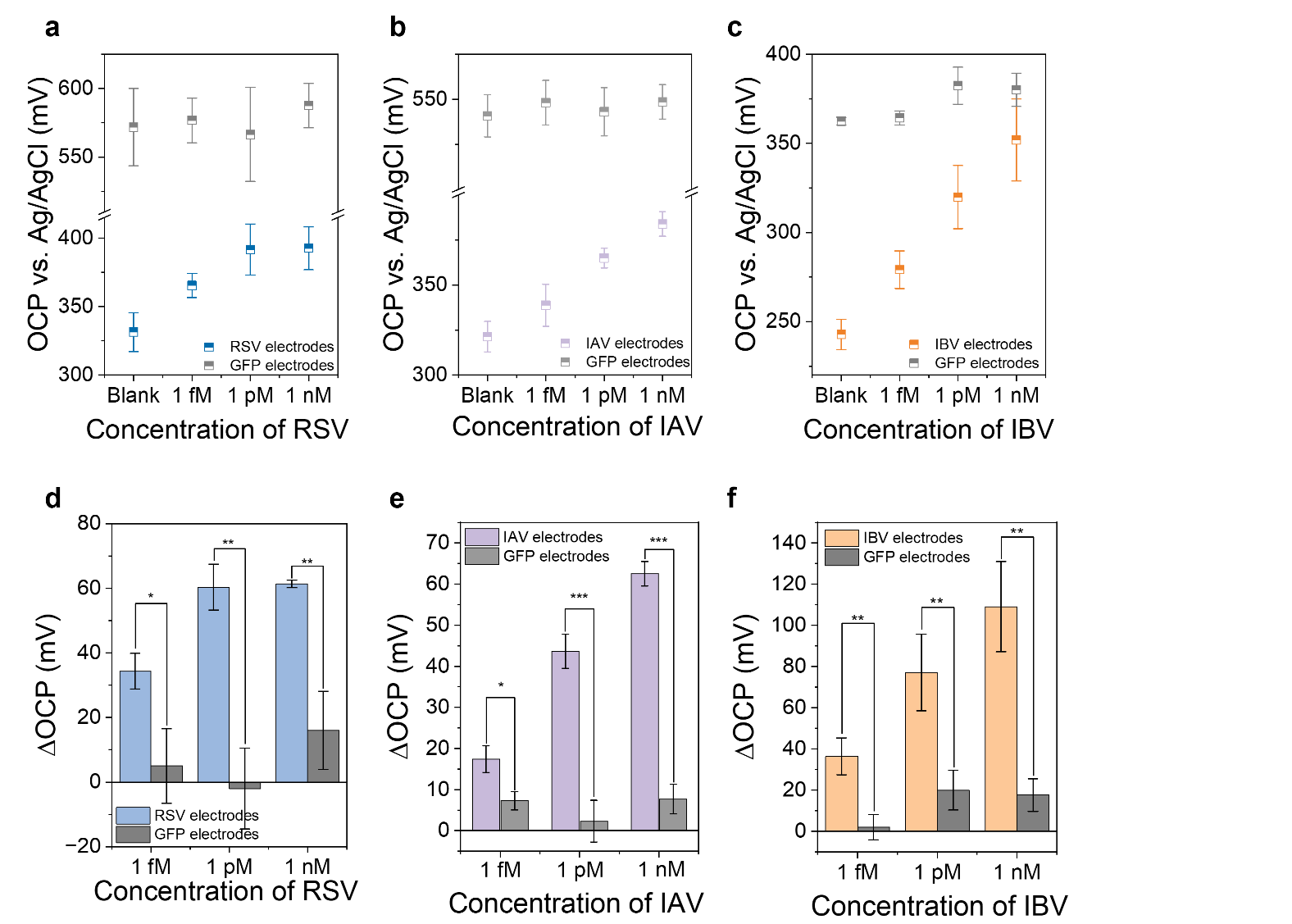


**Figure S24. Spike-protein tests of RSV, IAV, and IBV sensors.** OCP values of **(a)** GFP and RSV electrodes exposed to different concentrations of RSV, **(b)** GFP and IAV electrodes exposed to different concentrations of IAV targets, and **(c)** GFP and IBV electrodes exposed to different concentrations of IBV targets. Average OCP changes of **(d)** RSV electrodes and GFP electrodes, **(e)** IAV electrodes and GFP electrodes, and **(f)** IBV electrodes and GFP electrodes. n=3 for each electrode type. The proteins were spiked into human saliva.

**
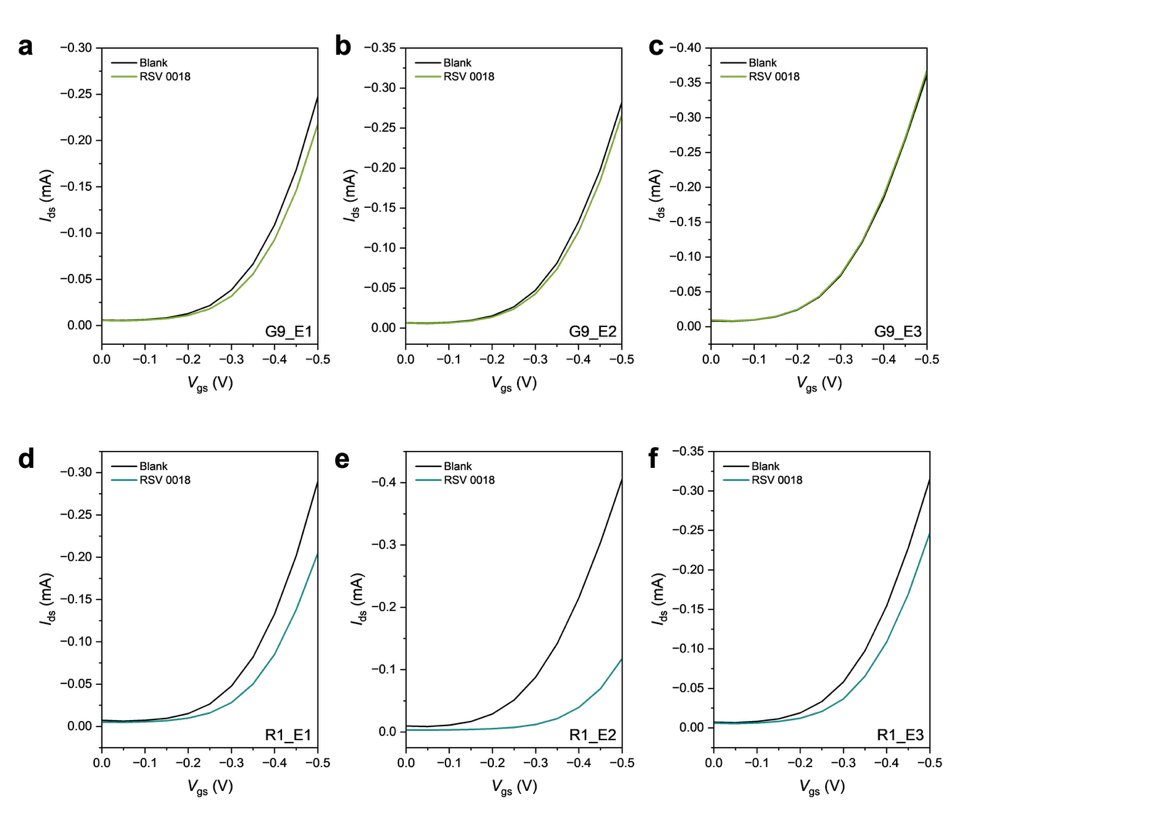
**

**Figure S25. Transfer curves obtained during RSV clinical sample analysis.** Transfer curves showing responses of controls **(a-c)** and RSV sensors **(d-f)** to sample labeled as RSV 0018.

**
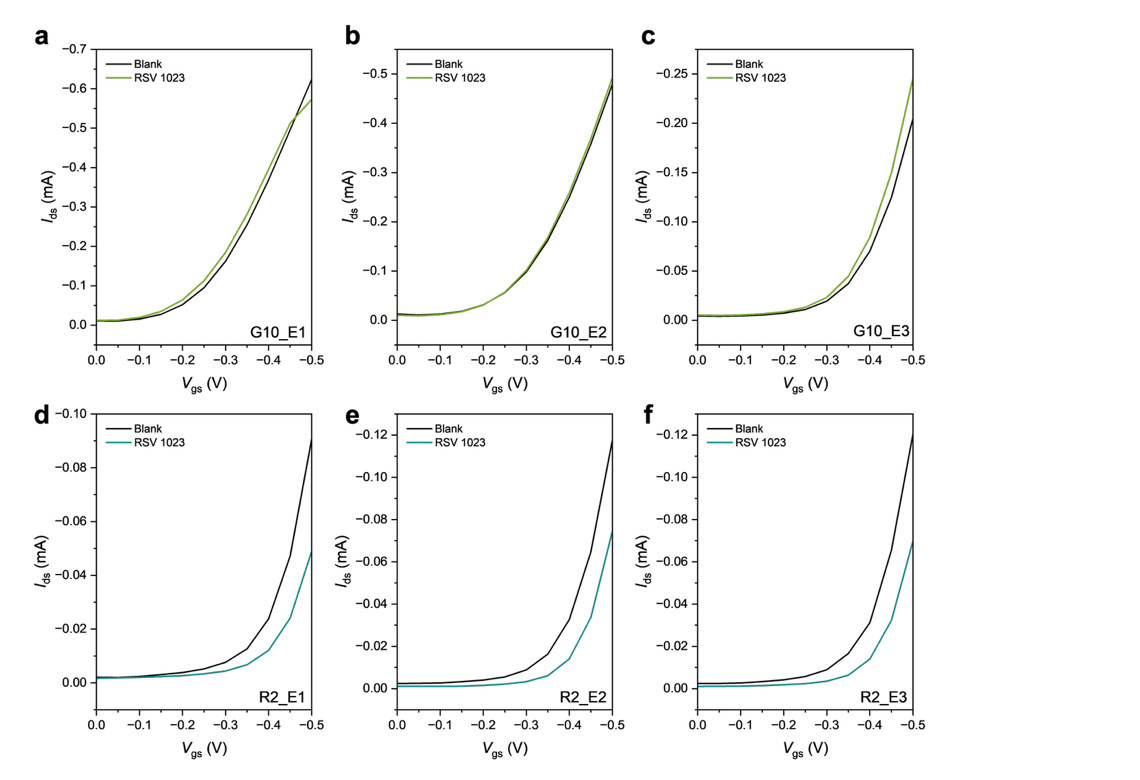
**

**Figure S26. Transfer curves obtained during RSV clinical sample analysis.** Transfer curves showing responses of controls **(a-c)** and RSV sensors **(d-f)** to sample labeled as RSV 1023.

**
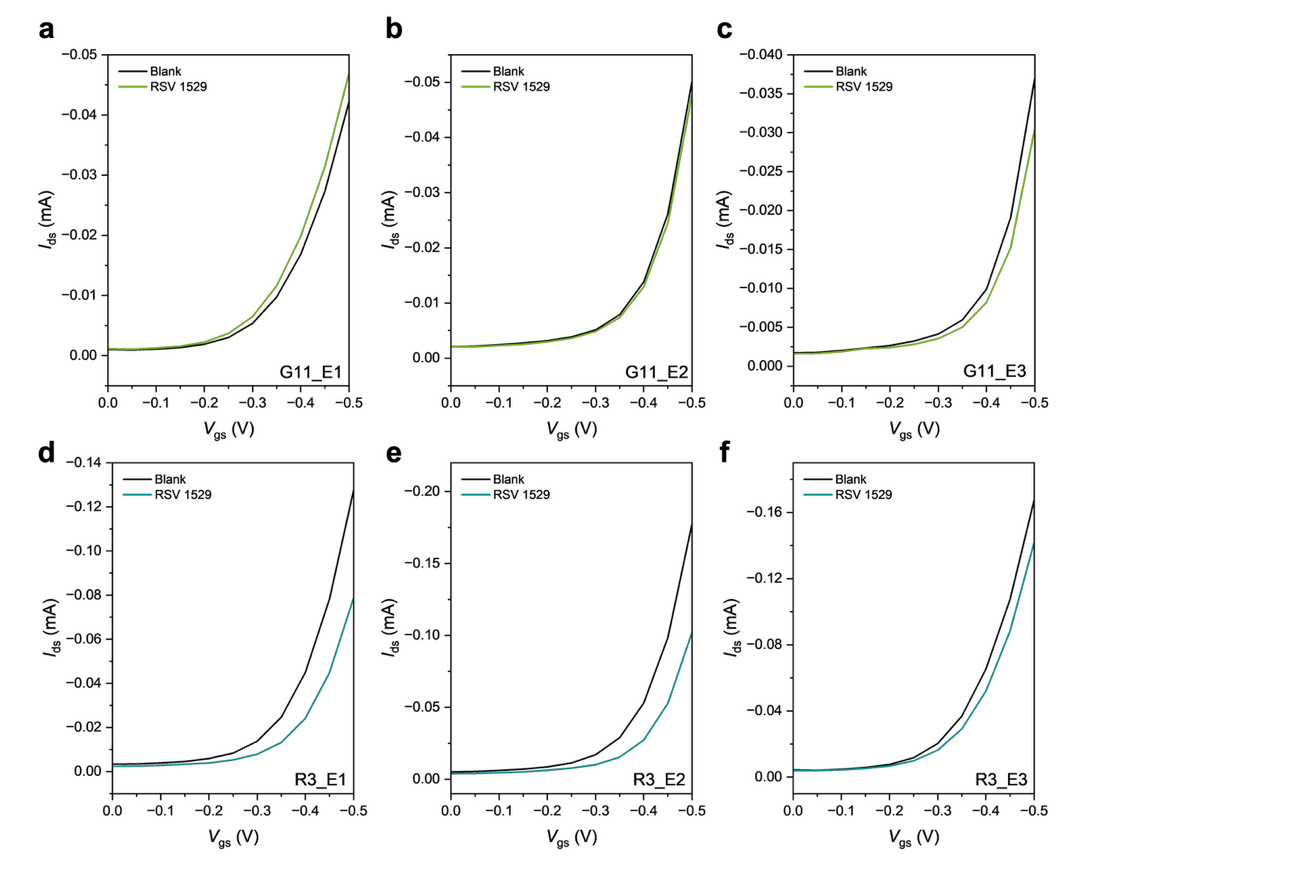
**

**Figure S27. Transfer curves obtained during RSV clinical sample analysis.** Transfer curves showing responses of controls **(a-c)** and RSV sensors **(d-f)** to sample labeled as RSV 1529.

**
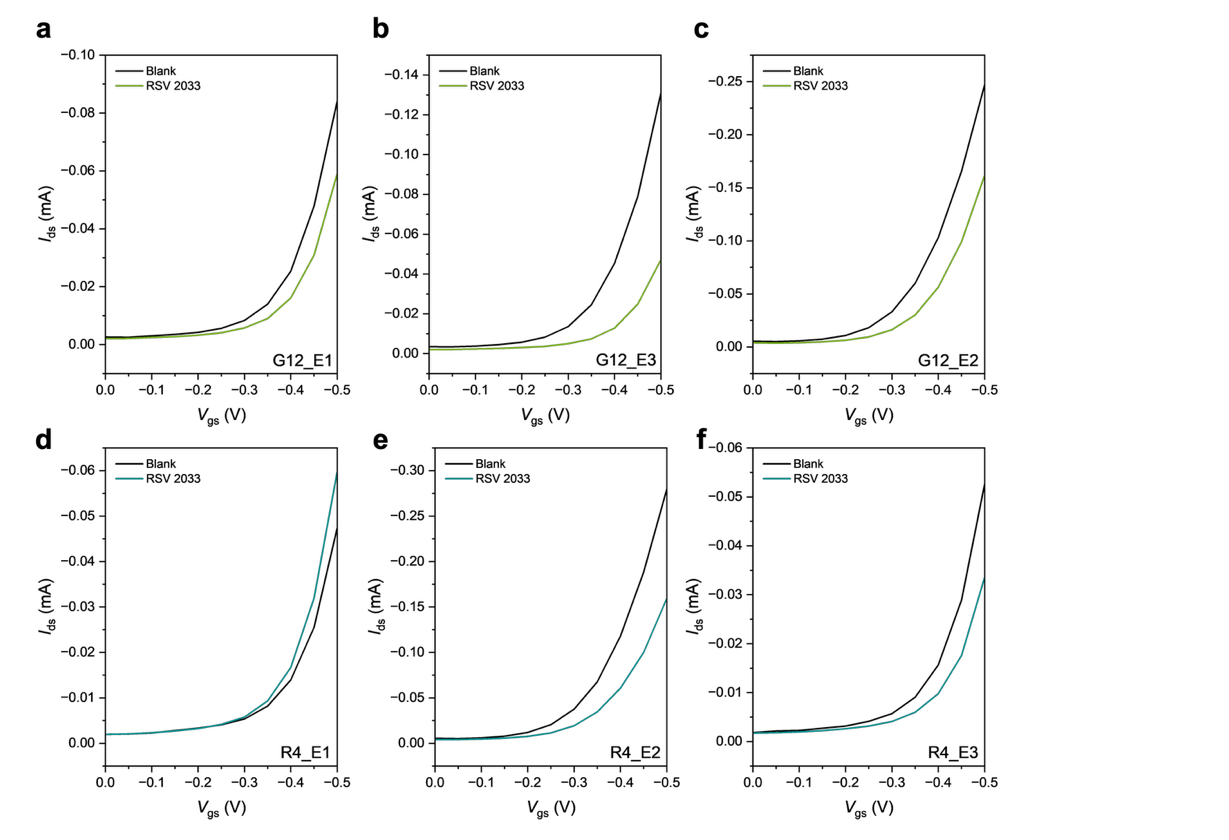
**

**Figure S28.** **Transfer curves obtained during RSV clinical sample analysis.** Transfer curves showing responses of controls **(a-c)** and RSV sensors **(d-f)** to sample labeled as RSV 2033.

**
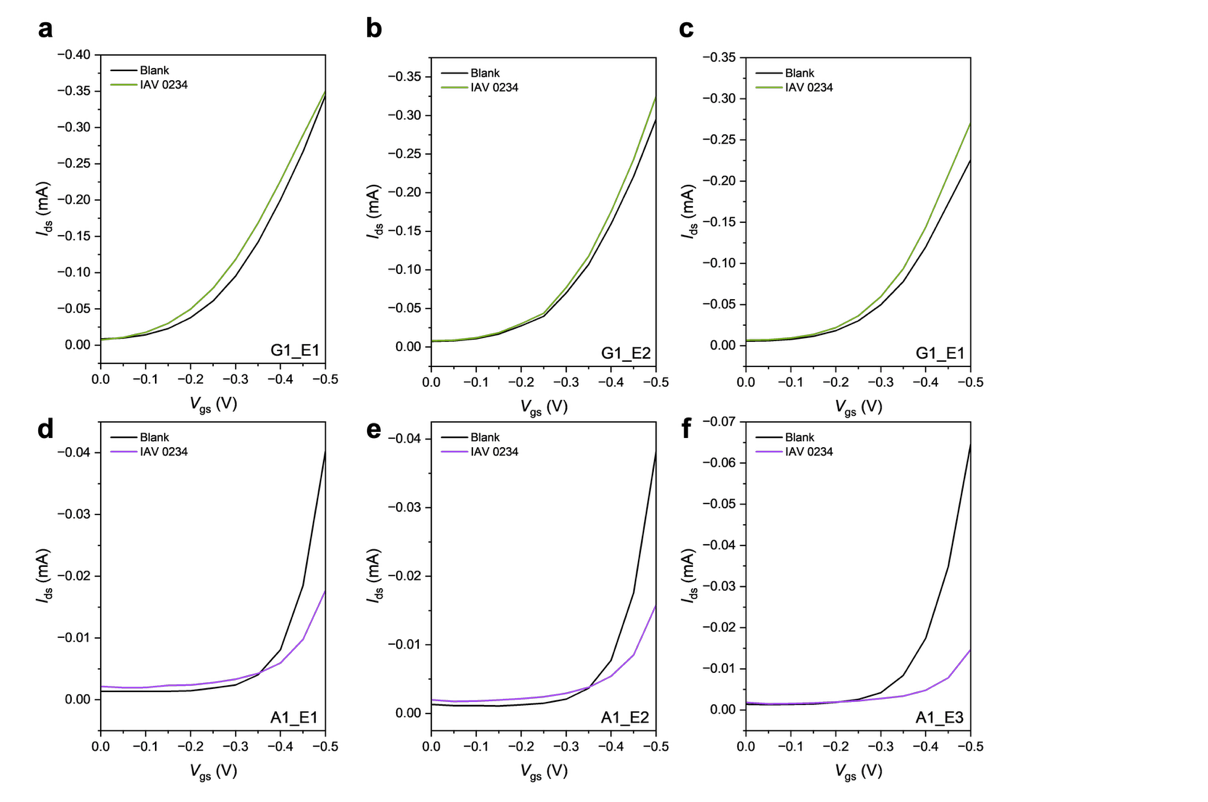
**

**Figure S29. Transfer curves obtained during IAV clinical sample analysis.** Transfer curves showing responses of controls **(a-c)** and IAV sensors **(d-f)** to sample labeled as IAV 0234.

**
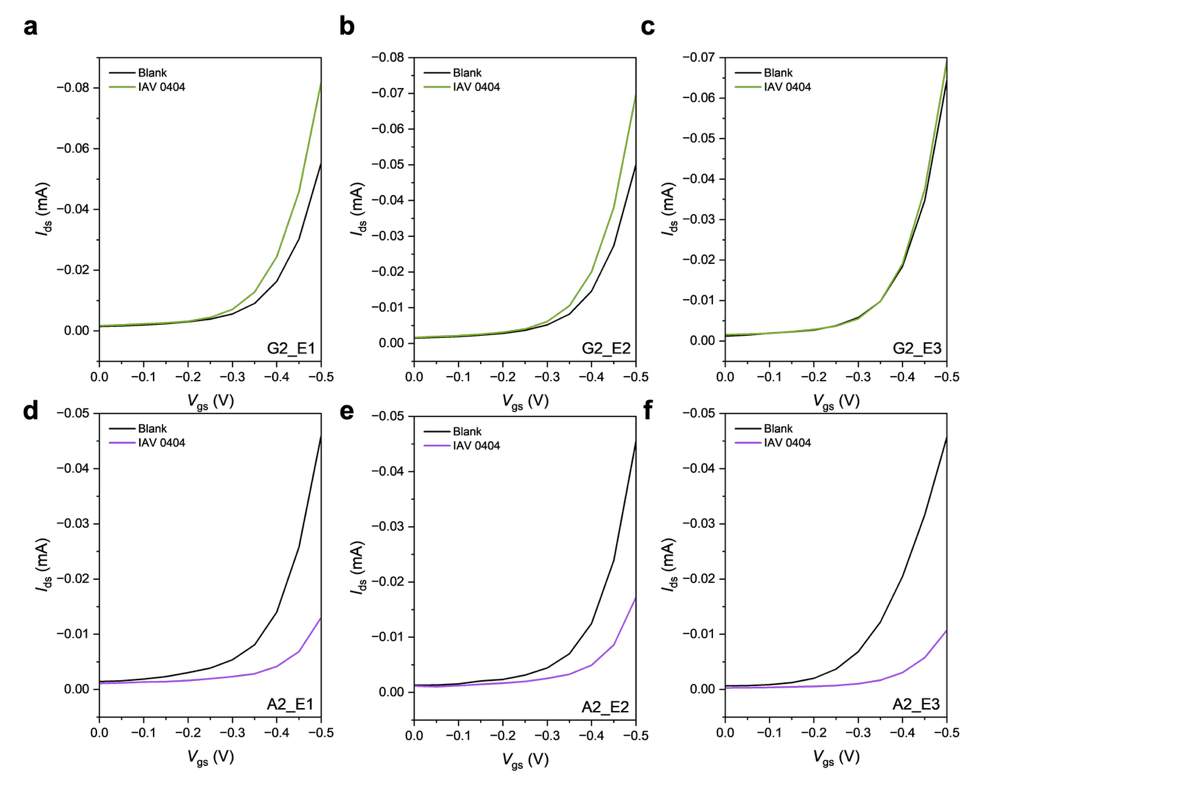
**

**Figure S30. Transfer curves obtained during IAV clinical sample analysis.** Transfer curves showing responses of controls **(a-c)** and IAV sensors **(d-f)** to sample labeled as IAV 0404.

**
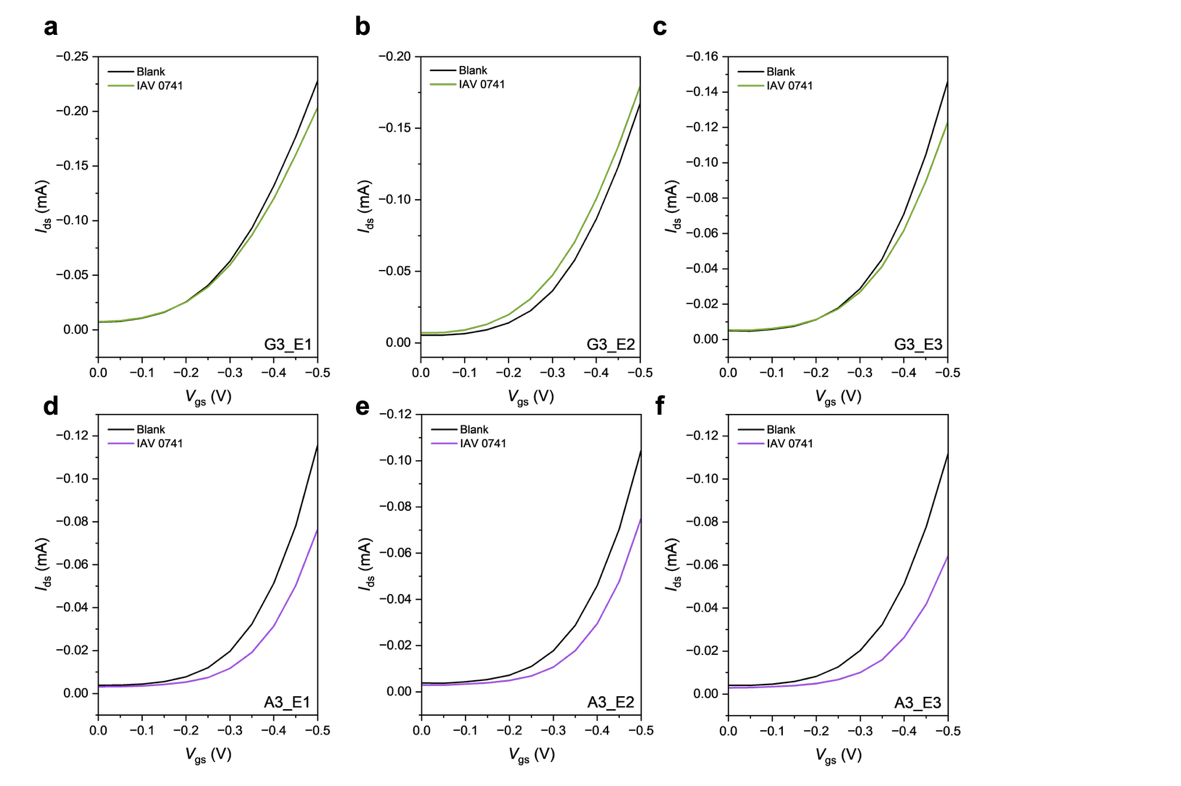
**

**Figure S31. Transfer curves obtained during IAV clinical sample analysis.** Transfer curves showing responses of controls **(a-c)** and IAV sensors **(d-f)** to sample labeled as IAV 0741.

**
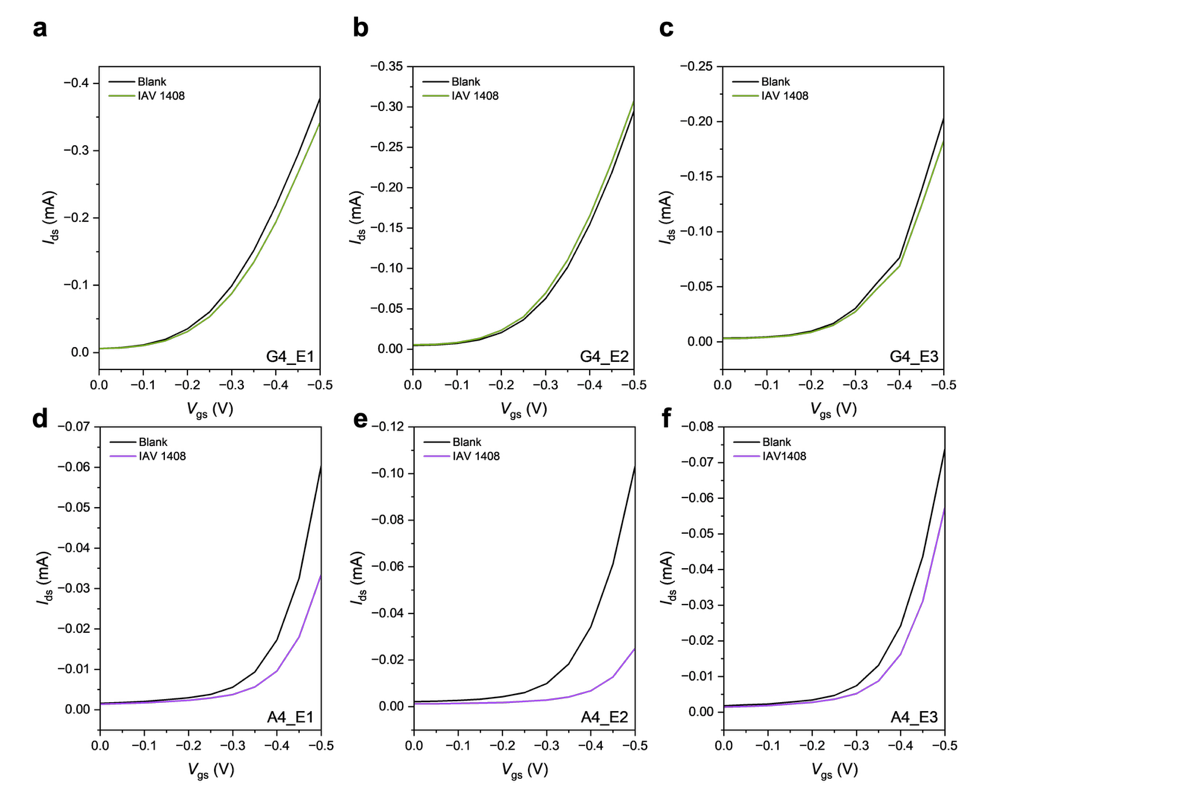
**

**Figure S32. Transfer curves obtained during IAV clinical sample analysis.** Transfer curves showing responses of controls **(a-c)** and IAV sensors **(d-f)** to sample labeled as IAV 1408.

**
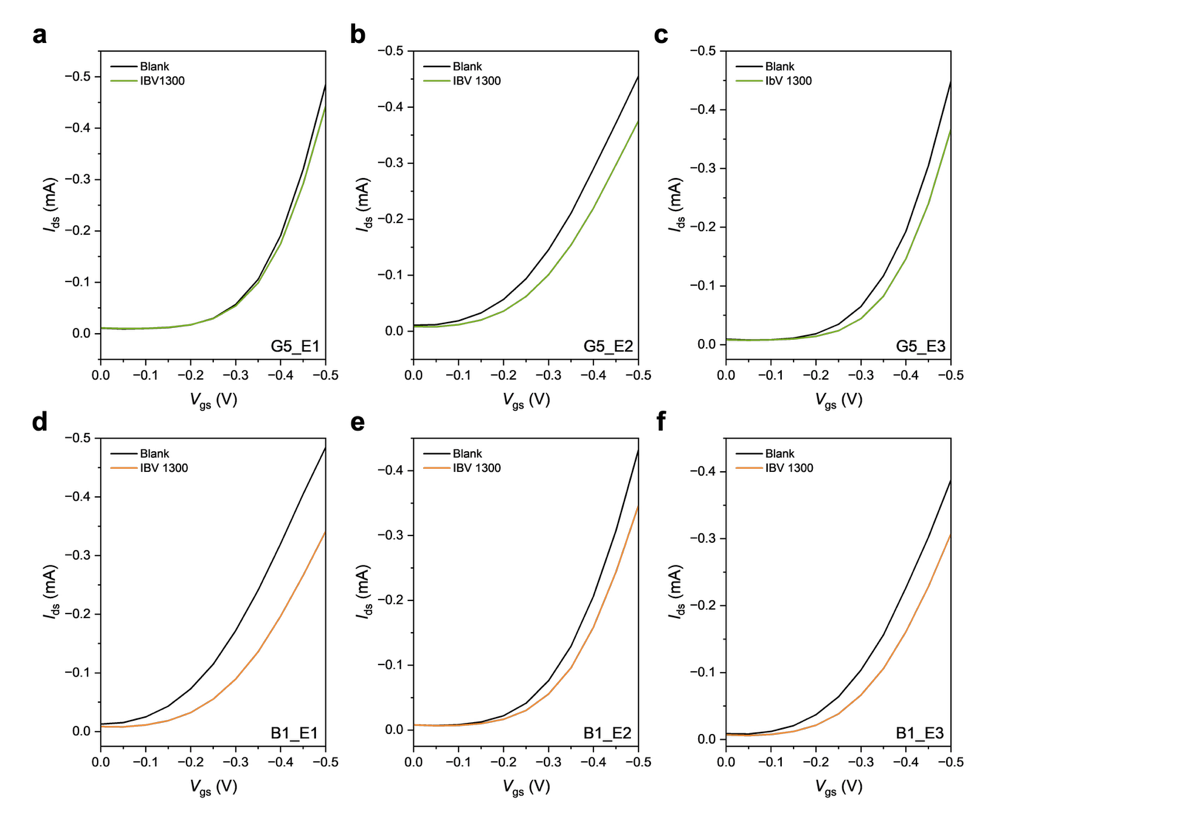
**

**Figure S33. Transfer curves obtained during IBV clinical sample analysis.** Transfer curves showing responses of controls **(a-c)** and IBV sensors **(d-f)** to sample labeled as IBV 1300.

**
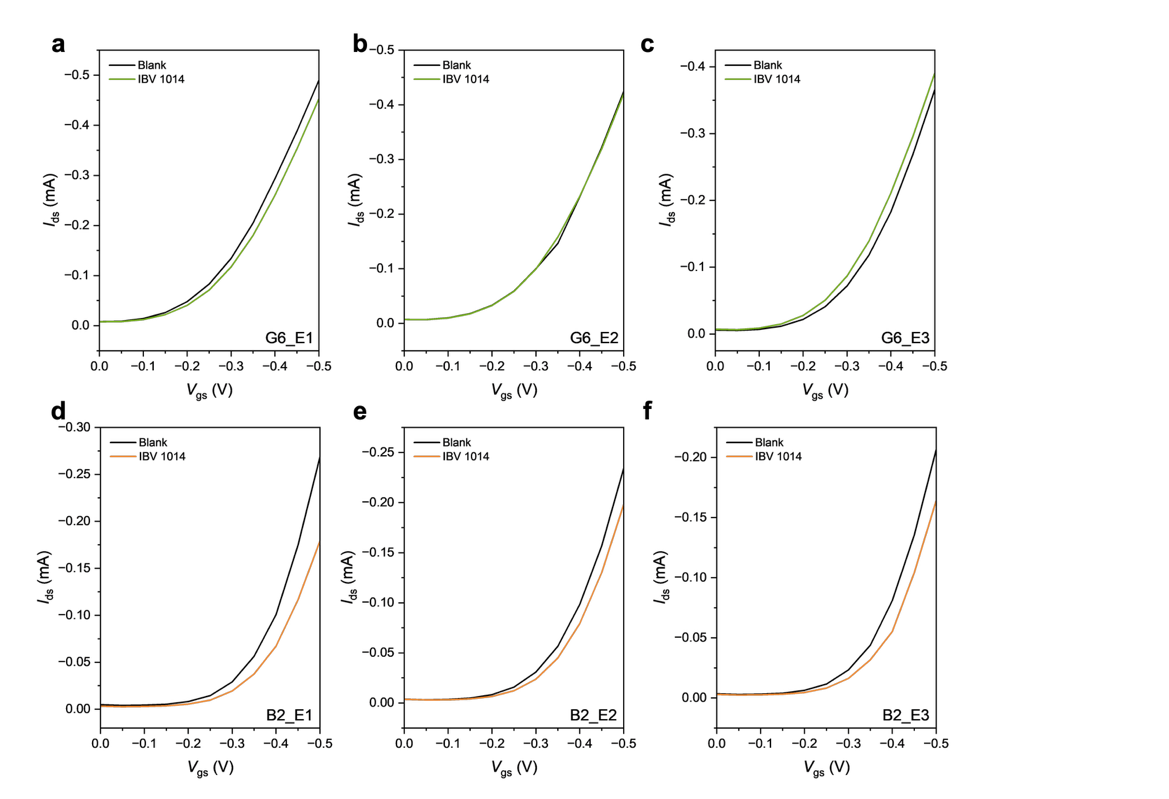
**

**Figure S34. Transfer curves obtained during IBV clinical sample analysis.** Transfer curves showing responses of controls **(a-c)** and IBV sensors **(d-f)** to sample labeled as IBV 1014.

**
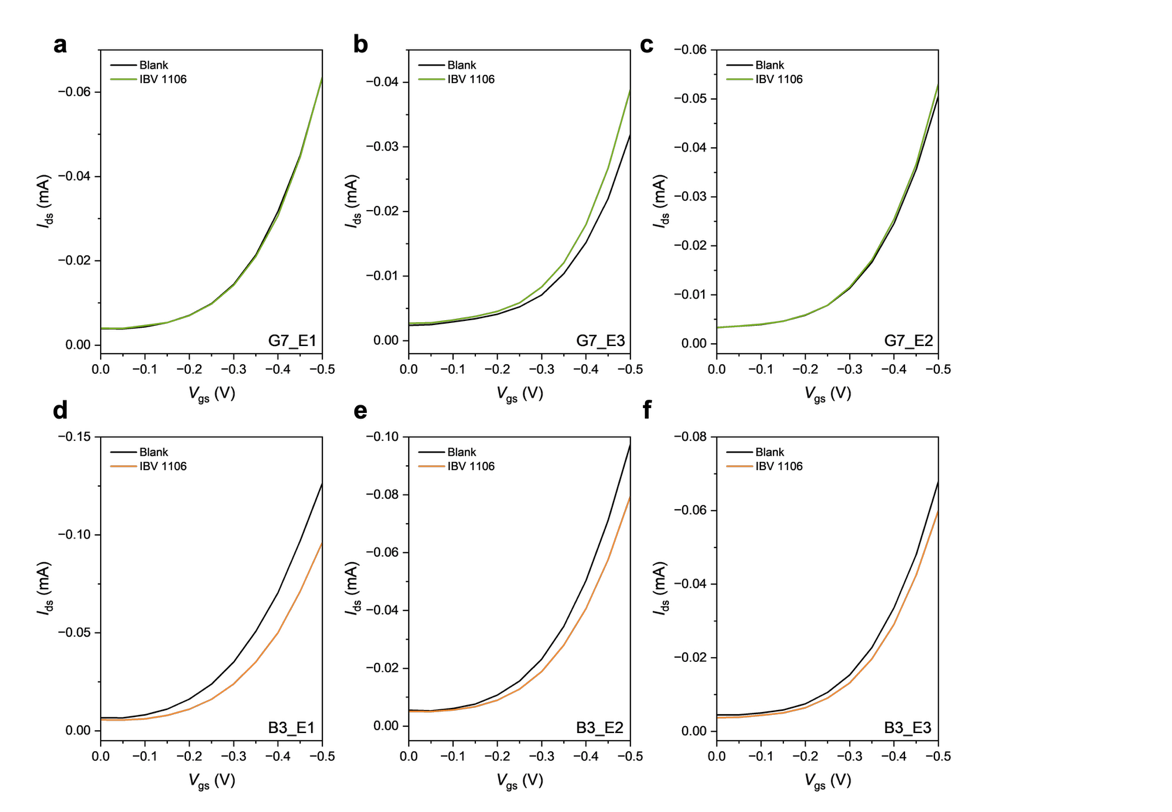
**

**Figure S35. Transfer curves obtained during IBV clinical sample analysis.** Transfer curves showing responses of controls **(a-c)** and IBV sensors **(d-f)** to sample labeled as IBV 1106.

**
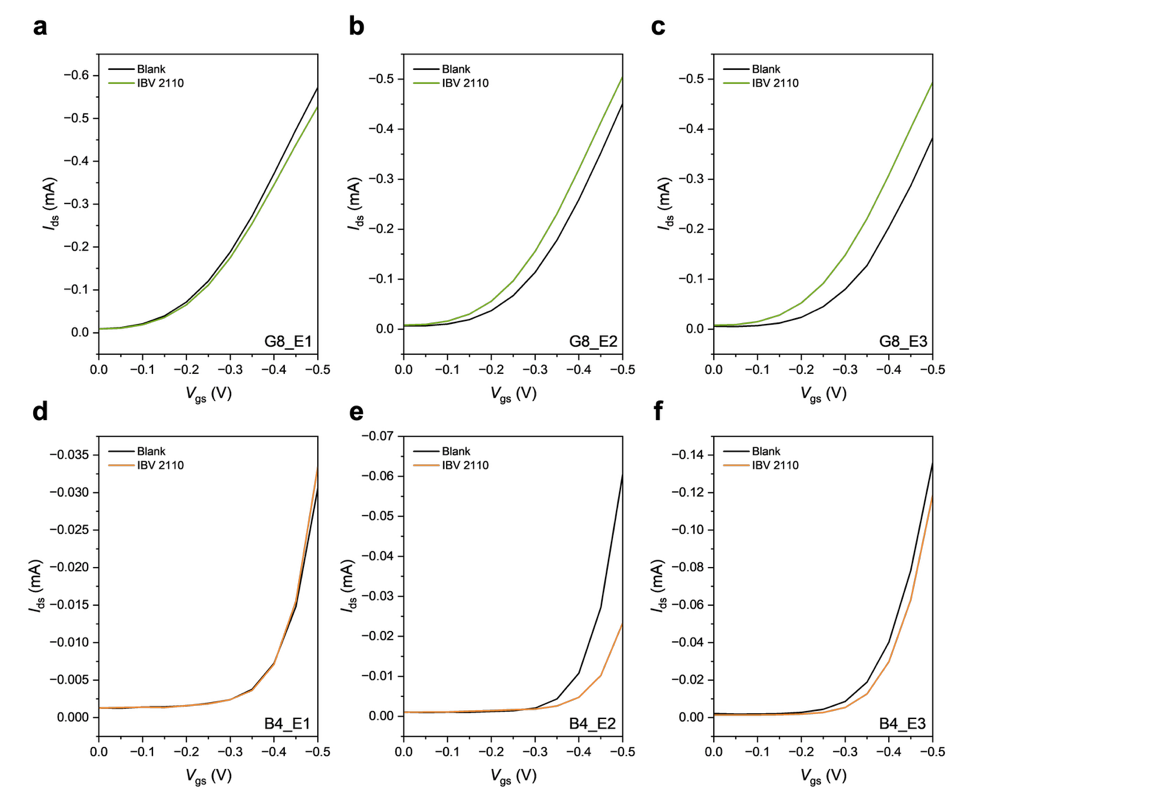
**

**Figure S36. Transfer curves obtained during IBV clinical sample analysis.** Transfer curves showing responses of controls **(a-c)** and IBV sensors **(d-f)** to sample labeled as IBV 2110.

**
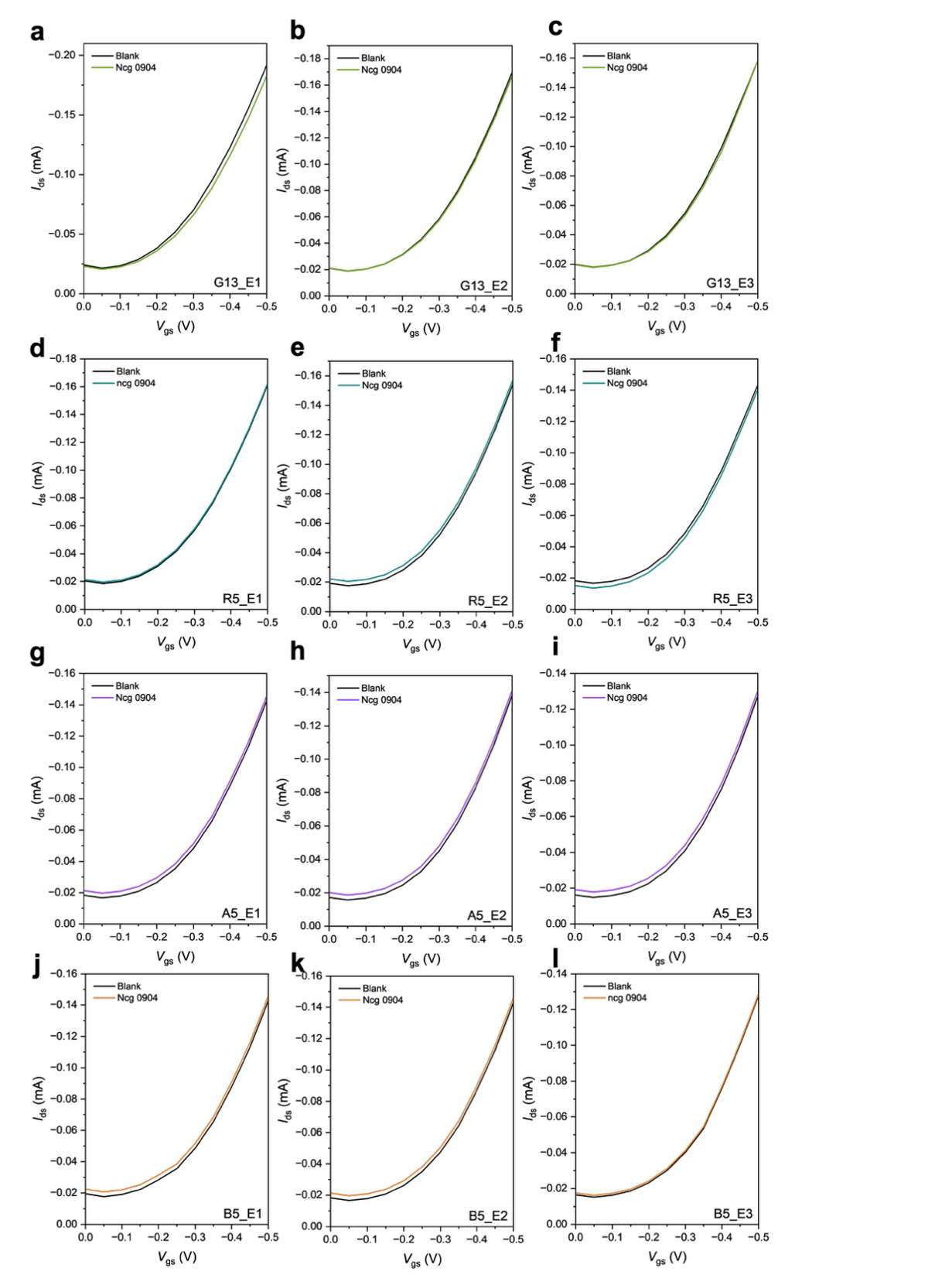
**

**Figure S37. Transfer curves obtained during virus-free clinical sample analysis.** Transfer curves showing responses of controls **(a-c)**, RSV sensors **(d-f),** IAV sensors **(g-i),** and IBV sensors **(j-l)** to sample Ncg 0904.

**
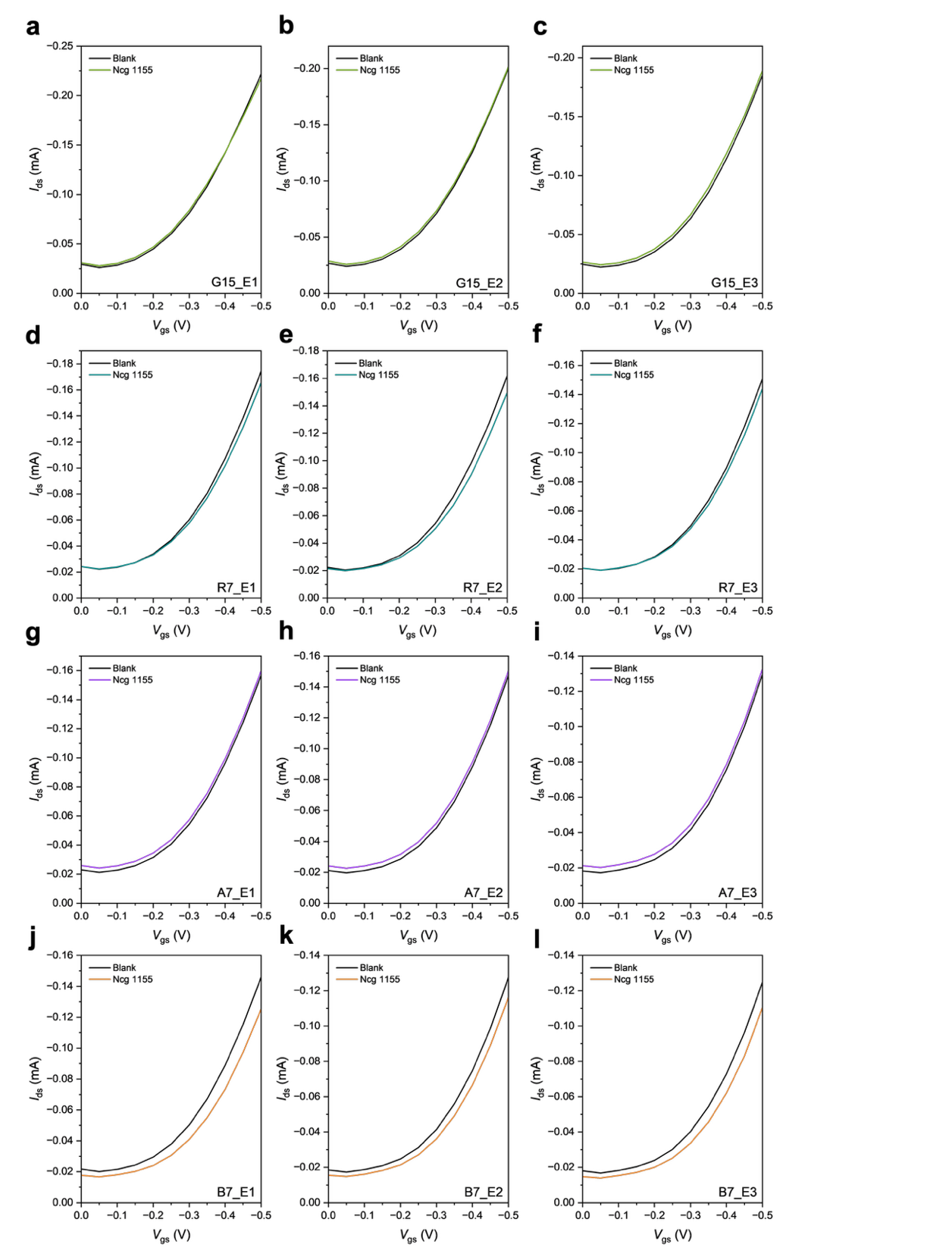
**

**Figure S38. Transfer curves obtained during virus-free clinical sample analysis.** Transfer curves showing responses of controls **(a-c)**, RSV sensors **(d-f),** IAV sensors **(g-i),** and IBV sensors **(j-l)** to sample Ncg 1155.

**
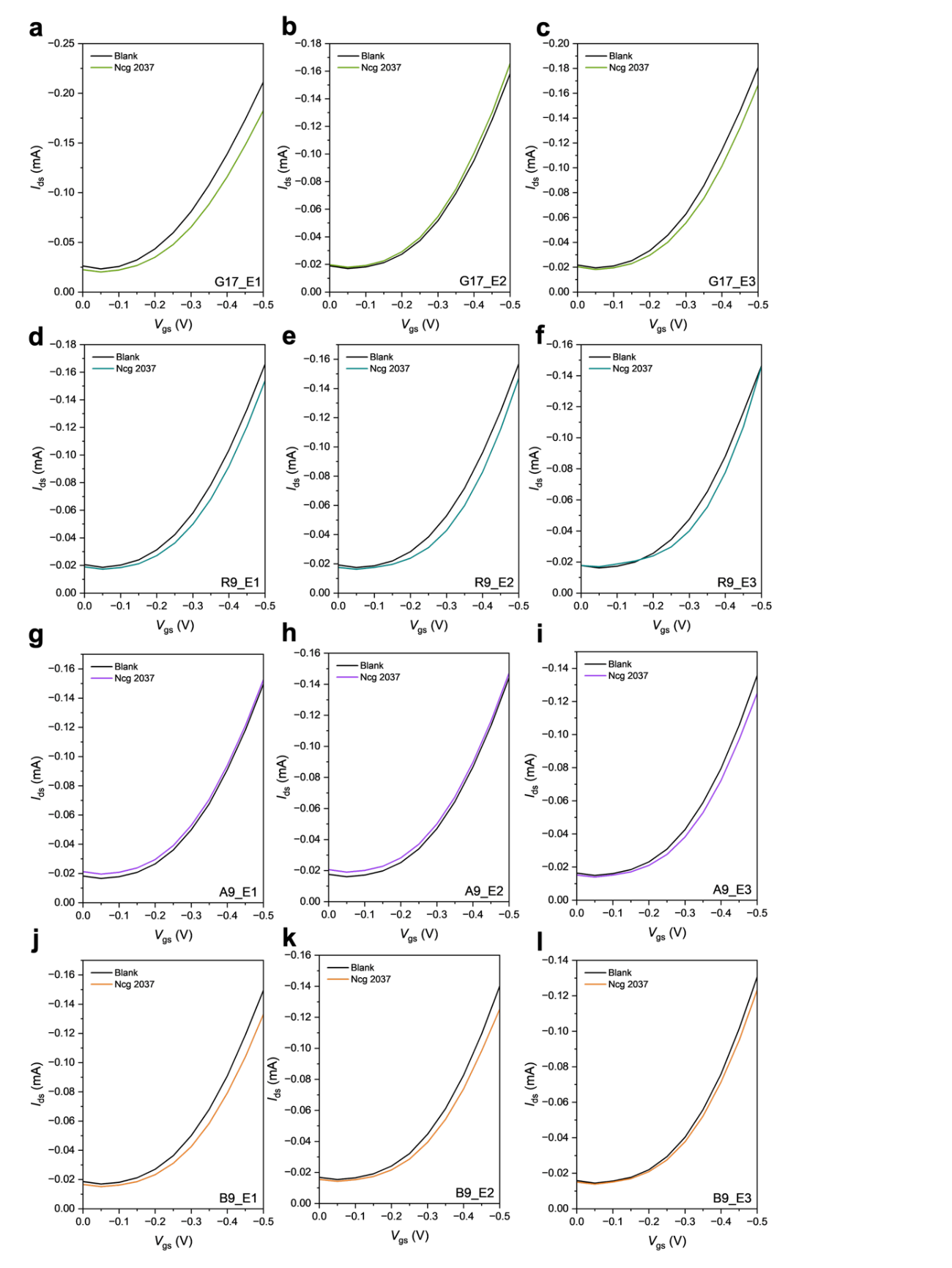
**

**Figure S39. Transfer curves obtained during virus-free clinical sample analysis.** Transfer curves showing responses of controls **(a-c)**, RSV sensors **(d-f),** IAV sensors **(g-i),** and IBV sensors **(j-l)** to sample Ncg 2037.

**
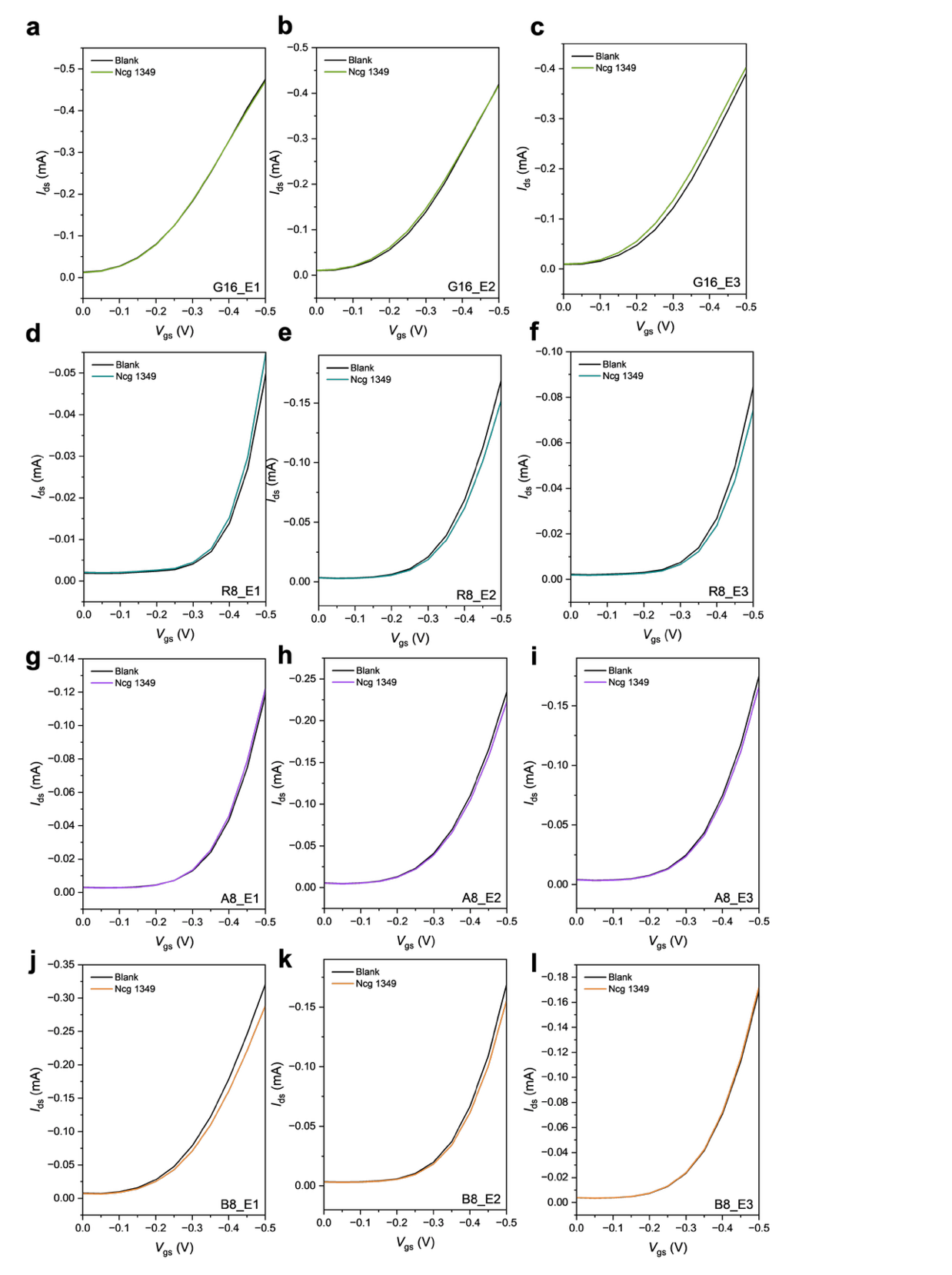
**

**Figure S40. Transfer curves obtained during virus-free clinical sample analysis.** Transfer curves showing responses of controls **(a-c)**, RSV sensors **(d-f),** IAV sensors **(g-i),** and IBV sensors **(j-l)** to sample Ncg 1349.

**
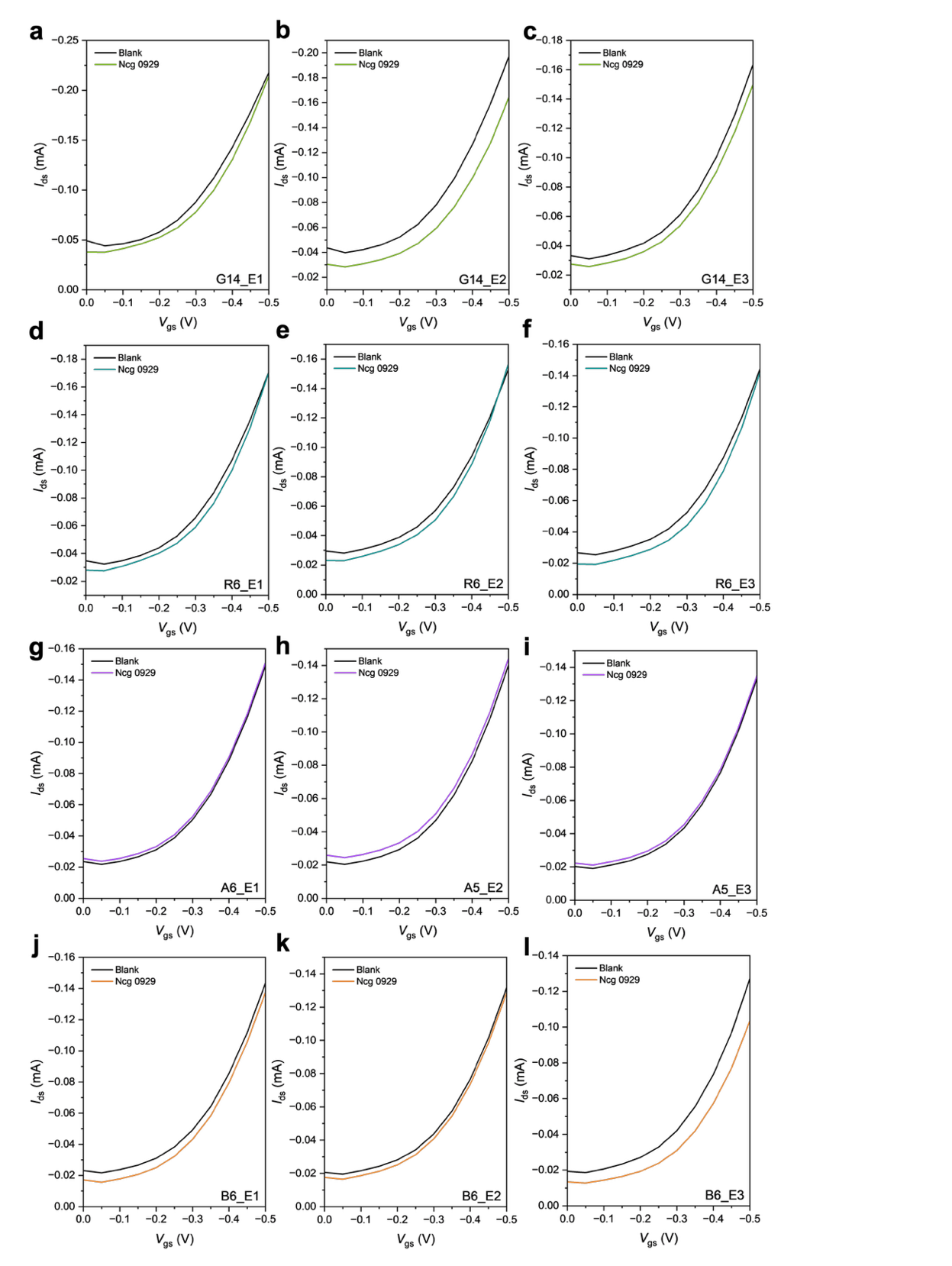
**

**Figure S41. Transfer curves obtained during virus-free clinical sample analysis.** Transfer curves showing responses of controls **(a-c)**, RSV sensors **(d-f),** IAV sensors **(g-i),** and IBV sensors **(j-l)** to sample Ncg 0929.


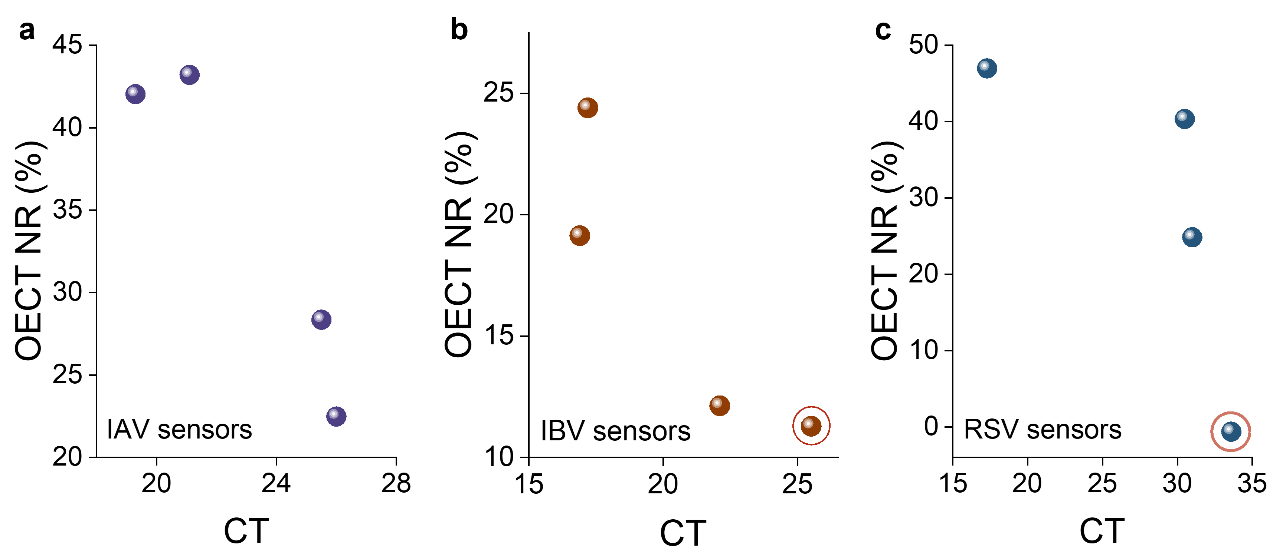


**Figure S42. Correlation between OECT sensor response and RT-qPCR CT values.** The OECT NR is calculated as the following equation: *OECT NR* = *NR*_sensor_ – *NR*_control_, where *NR*_sensor_ is the normalized response of IAV/IBV/RSV sensors, and the *NR*_control_ is the normalized response of GFP controls exposed to the same samples. Red circles in **(b)** and **(c)** correspond to samples #2110 and #2033 that we failed to diagnose.

**Table S1. Summary of binding analysis between nanobody fusion proteins and their target proteins.** The nanobodies that are selected to build the sensors are highlighted in bold.

| **Target** | **Nanobody** | **Nanobody- SpyCatcher fusion^a^** | **K_D_ (nM)** | **SNR^b^** |
| --- | --- | --- | --- | --- |
| RSV-F | **Nb017** | **sb0330p** | **236.0 ± 121.5** | **23.1** |
| NP of IAV | NP1 | sb0331p | 564.4 ± 71.3 | 14.3 |
|  | NP54 | sb0332p | 1034.6 ± 382.2 | 15.0 |
|  | NP170 | sb0333p |  |  |
| HA1 of IAV | R1a-B6 | sb0334p | 68.8 ± 2.1 | 7.4 |
|  | R1a-G6 | sb0335p | 32.2 ± 1.6 | 9.6 |
|  | **R2b-D9** | **sb0336p** | **19.2 ± 1.1** | **5.4** |
| HA of IBV | SD83 | sb0337p | - |  |
|  | **SD84h** | **sb0338p** | **21.2 ± 2.5** | **2.2** |

^a^ construct name and identification number; ^b^ SNR: MST signal-to-noise ratio.

**Table S2. △*f* and △D monitored during SpyTag peptide binding, SpyCatcher/nanobody binding and target binding to their respective sensor surfaces.**

|  | **SpyTag** | | | **Nanobody-SpyCatcher** | | | **Target protein** | | |
| --- | --- | --- | --- | --- | --- | --- | --- | --- | --- |
|  | **△*f*** | **△*D*** | **Density**  **(×10^13^ / cm^2^)** | **△*f*** | **△*D*** | **Density**  **(×10^12^ / cm^2^)** | **△*f*** | **△*D*** | **Density**  **(×10^12^ / cm^2^)** |
| **RSV** | -9.62 | +0.03 | 6.72 | -17.52 | +0.58 | 7.27 | -9.82 | +1.58 | 1.97 |
| **IAV** | -11.28 | +0.06 | 6.59 | -22.00 | +0.51 | 8.19 | -4.51 | +0.90 | 0.76 |
| **IBV** | -9.45 | +0.08 | 5.66 | -30.15 | +1.38 | 11.52 | -6.54 | +1.49 | 0.99 |

**Table S3. △*f* monitored during SpyTag peptide and SpyCatcher/GFP nanobody functionalization and the incubation with blocking molecules.**

|  | **SpyTag** | | **GFP Nb- SpyCatcher** | | **Blocker type** | **Blocker-triggered**  **△*f* (Hz)** |
| --- | --- | --- | --- | --- | --- | --- |
|  | **△*f* (Hz)** | **Density (×10^13^ / cm^2^)** | **△*f* (Hz)** | **Density**  **(×10^12^ / cm^2^)** |  |  |
| **G1** | -9.87 | 8.54 | -17.06 | 9.27 | +PBS (no blocker) | +3.51 |
| **G2** | -10.92 | 9.45 | -15.31 | 8.32 | +10 nM Lysozyme | -2.16 |
| **G3** | -12.41 | 10.73 | -16.33 | 8.88 | +10 nM Casein | -5.65 |
| **G4** | -10.23 | 8.85 | -17.51 | 9.52 | +10 nM BSA | +1.07 |

**Table S4. △*f* and △OCP of the GFP nanobody functionalized electrodes when exposed to target (GFP) and a non-target protein (IBV).** G1 has no blocker, G2 was modified with lysozyme, G3 with casein and G4 with BSA.

|  | **+10 nM IBV**  **(Non-target protein)** | | **+10 nM GFP**  **(Target protein)** | | |
| --- | --- | --- | --- | --- | --- |
|  | **△*f* (Hz)** | **△OCP (mV)** | **△*f* (Hz)** | **Density (×10^12^ / cm^2^)** | **△OCP (mV)** |
| **G1** | -0.77 | -23.8 | -22.44 | 8.33 | -47.09 |
| **G2** | -0.56 | 2.08 | -18.52 | 7.18 | 25.60 |
| **G3** | -1.10 | 14.50 | -20.87 | 8.47 | 25.67 |
| **G4** | -0.72 | -25.99 | -19.23 | 8.57 | -37.69 |

**Supplementary Note 1: The role of electrode pre-treatment in eliminating false negatives**

In this study, we used GFP Nb-functionalized electrodes as the negative control to verify the specificity of target binding. We confirmed the successful biofunctionalization of the GFP nanobody using QCM-D analysis (**Figure S10a** and **Figure S12**). The sensors reached an average SpyTag density of (9.39 ± 0.83) v 10^13^/cm^2^, and the average GFP Nb density is (9.00 ± 0.45) × 10^12^/cm^2^, showing Nb occupancy of 9.00 % ± 0.45 % (**Table S3**). This number is similar to the RSV, IAV, and IBV-Nb data, indicating the consistency of both the QCM-D technology and the surface functionalization method. As expected, GFP Nb functionalised electrodes exhibited no binding affinity toward the three investigated targets (RSV, IAV, and IBV), as supported by our QCM-D studies (**Figure S10b and d**). Conversely, the addition of 10 nM GFP protein led to a large frequency decrease and an increase in dissipation, confirming the specific affinity of GFP proteins toward their corresponding nanobodies.

However, despite the absence of specific interactions, exposure of these electrodes to other proteins caused a pronounced increase in its OCP (**Figure S10c and e**). Repeated 1× PBS incubation only resulted in minor OCP increases (within 3 mV), indicating stable baseline behavior (**Figure S11**). In contrast, incubation with IAV protein caused a pronounced OCP increase, suggesting that non-specific molecular interactions affected surface potential even though the non-target molecules did not stay on the nanobodies permanently (washed away after the rinsing). These results suggest that some other species (e.g. glycerol, salt ions) left after the dilution of the protein solution may be polarizing/depolarizing the electrode. The unstable OCP of the untreated GFP electrode rendered it unreliable as a negative control.

To address this issue and stabilize the OCP of this negative control electrode, we developed a pre-treatment strategy, which involved overnight incubation of all four electrodes with low concentration blocking molecules, such as 10 nM bovine serum albumin (BSA), 10 nM casein, and 10 nM lysozyme, before using them in protein sensing. The detailed results are presented in **Figures S12-S16, Tables S3-S4**. Specifically, we monitored whether these molecules stick to the GFP-electrode surface after incubation overnight but did not observe any mass accumulation (**Figure S13**, **Table S3**). Regardless of the blocking molecule used, nanobody specificity remained intact (**Figure S14**, see QCM-D data and the analysis results in **Figure S15**) with target GFP protein binding reaching an average of (8.13 ± 0.56) × 10^12^ molecules /cm^2^ (**Table S4**), suggesting 90.39 % ± 3.24 % occupancy. However, we again observed OCP variations from all electrodes, except the one treated with lysozyme, when the electrodes were exposed to non-target proteins (see **Figure S14** for OCP data). Only lysozyme-treated electrodes exhibited minimal OCP drift after exposure to non-target proteins (**Table S4**), identifying lysozyme as the optimal blocking agent.

To validate the performance of these lysozyme-treated GFP-functionalized electrodes as negative control, we incubated them with a mixture of RSV, IAV, and IBV proteins, from low to high concentrations, while recording OCP responses. As shown in **Figure S16**, the GFP electrode exhibited random OCP fluctuations within 5 mV, confirming no particular response to non-target proteins. We further validated the pre-treatment strategy using IBV-targeting electrodes alongside GFP Nb functionalized electrodes as controls. The pre-treated GFP electrodes remained stable under non-target incubation conditions, while the IBV electrodes responded selectively to increasing concentrations of IBV protein (**Figure S17**). These results establish lysozyme pre-treatment as essential for ensuring surface potential stability of the electrode by effectively minimizing non-target interactions and reliable measurements in multiplexed sensing.

**Supplementary Note 2: Evaluation of reusability and storage stability of the sensors**

To assess long-term stability, we systematically investigated the evolution of sensing performance under different storage conditions (4 °C and room temperature). A set of RSV nanobody-functionalized sensors (3 RSV sensors and 3 GFP controls) was first fabricated and characterized immediately after preparation (**Figure S23a**). The measurement protocol included two baseline recordings in 1× PBS, followed by rinsing in 0.001× PBS. Sensors were then exposed sequentially to 40 fM and 4 pM RSV targets, with transfer curves recorded after each step. The normalized response (NR) was extracted for each case, by considering the baseline recordings. Baseline signals remained within 5%, indicating stable device operation. RSV-functionalized sensors showed clear and reproducible responses compared to GFP controls, consistent with the calibration data (**Figure 4d**), with an inter-chip coefficient variation (CV) of 18.76% at 4 pM (**Figure S23i**).

For storage stability, eight additional sensor sets (each comprising 3 RSV sensors and 3 GFP controls) were prepared and divided into two groups: storage in 1× PBS at 4 °C or at room temperature. Performance was evaluated after 7, 14, 21, and 28 days for sensors stored at 4 °C, and after 7, 14, and 21 days for sensors stored at room temperature using the same protocol.

Sensors stored at 4 °C maintained stable responses for up to 14 days, with 4-7% signal reduction (**Figure S23b-c**). After 21 days, a marked degradation was observed, leading to near-complete loss of sensing function (**Figure S23d-e**). Sensors stored at room temperature showed faster degradation, with >10% signal loss by day 7 and increased variability by day 14 (**Figure S23f, g, i**), followed by loss of functionality after 21 days (**Figure S23h**). The CV analysis (**Figure S23i**) suggests that lower-temperature storage suppressed performance degradation and variability during the early days of storage. Overall, the sensors exhibited acceptable stability for up to two weeks when stored at 4 °C.

To elucidate the origin of performance degradation, electrochemical impedance spectroscopy (EIS) measurements were performed for sensors stored at 4 °C. The charge transfer resistance (*R*_ct_) decreased by ~11% after 14 days and by >35% after 21 days (**Figure S23j**), consistent with the loss of sensing performance. The reduced interfacial resistance is likely due to partial desorption or structural degradation of the nanobody layer. As the nanobody layer acts as a steric and dielectric barrier, its degradation exposes the electrode surface. These results suggest that the observed performance loss is primarily driven by degradation of the biofunctional layer rather than intrinsic instability of the OECT device.
